# A direct observation of up-converted room-temperature phosphorescence in an anti-Kasha dopant-matrix system

**DOI:** 10.1038/s41467-023-37662-y

**Published:** 2023-04-08

**Authors:** Jiuyang Li, Xun Li, Guangming Wang, Xuepu Wang, Minjian Wu, Jiahui Liu, Kaka Zhang

**Affiliations:** Key Laboratory of Synthetic and Self-Assembly Chemistry for Organic Functional Molecules, Shanghai Institute of Organic Chemistry, University of Chinese Academy of Sciences, Chinese Academy of Sciences, 345 Lingling Road, Shanghai, 200032 People’s Republic of China

**Keywords:** Optical materials and structures, Materials for optics

## Abstract

It is common sense that emission maxima of phosphorescence spectra (*λ*_P_) are longer than those of fluorescence spectra (*λ*_F_). Here we report a serendipitous finding of up-converted room-temperature phosphorescence (RTP) with *λ*_P_ < *λ*_F_ and phosphorescence lifetime > 0.1 s upon doping benzophenone-containing difluoroboron β**-**diketonate (BPBF_2_) into phenyl benzoate matrices. The up-converted RTP is originated from BPBF_2_’s T_n_ (*n* ≥ 2) states which show typical ^3^n-π* characters from benzophenone moieties. Detailed studies reveal that, upon intersystem crossing from BPBF_2_’s S_1_ states of charge transfer characters, the resultant T_1_ and T_n_ states build T_1_-to-T_n_ equilibrium. Because of their ^3^n-π* characters, the T_n_ states possess large phosphorescence rates that can strongly compete RTP(T_1_) to directly emit RTP(T_n_) which violates Kasha’s rule. The direct observation of up-converted RTP provides deep understanding of triplet excited state dynamics and opens an intriguing pathway to devise visible-light-excitable deep-blue afterglow emitters, as well as stimuli-responsive afterglow materials.

## Abstract (web summary)

The emission maximum of phosphorescence is normally larger than that of fluorescence. Here, authors report up-converted room-temperature phosphorescence materials that emit from higher-lying triplet states with 3n-π* characters and large phosphorescence decay rate constants, violating Kasha’s rule.

## Introduction

Manipulation of excited states represents a central topic in the fields of photofunctional materials. A deep understanding of triplet excited state property is of vital importance for devising high-performance room-temperature phosphorescence (RTP) and other luminescent materials^1–10^. Besides the manipulation of T_1_ states, control of the photophysical behaviors of higher triplet excited states (T_n_, *n* ≥ 2) is also very important because T_n_ states can mediate S_1_ to T_1_ intersystem crossing (ISC) in organic RTP systems and serve as candidates for fabrication of RTP materials with intriguing properties^11–17^. For example, it is well-known that T_2_ states of ^3^π-π* characters in benzophenone systems can strongly facilitate ISC from ^1^n-π* (S_1_ state) to ^3^n-π* (T_1_ state)^2,12^. Benzophenone systems possess very high Φ_ISC_ but relatively short phosphorescence lifetimes on the order of 0.1 to 1.0 ms; T_1_ state of ^3^n-π* characters has large phosphorescence decay rate constants (*k*_P_). Recent studies show the achievement of both high RTP quantum yields (Φ_P_) and relatively long phosphorescence lifetimes (*τ*_P_) by adjusting n-π* and π-π* compositions in S_1_ and T_1_ states, as well as in T_n_ (*n* ≥ 2) states^13–15^. Molecular aggregation has also been demonstrated to control the properties of T_n_ (*n* ≥ 2) states^11,18–20^. Upon aggregation, the electronic interactions between chromophores can cause energy splitting to give rise to multiple close-lying T_n_ states^19,20^. As a result, more S_1_-T_n_ channels with relatively large spin-orbit coupling matrix elements (SOCME) and small Δ*E*_ST_ can be generated to enhance ISC processes.

Despite of these essential roles of T_n_ (*n* ≥ 2) states to facilitate ISC, the studies, and understanding on T_n_ states are mostly restricted to computational studies and ultrafast spectroscopy^21–23^. Photophysical behaviors of T_n_ (*n* ≥ 2) states remain rarely observed and reported in conventional experimental conditions because T_n_-T_1_ internal conversion is usually much faster than T_n_-S_0_ phosphorescence decay according to Kasha’s rule^24,25^. We reason that a direct observation of RTP(T_n_) (RTP from T_n_ state, *n* ≥ 2) would be very important from at least three aspects. First, the observation of RTP(T_n_) by conventional experimental setups and even human eyes can give a straightforward understanding on excited state dynamics of T_n_ (*n* ≥ 2) states, including their population, conversion, and decay. Second, RTP(T_n_) exhibits smaller Stokes shift than RTP(T_1_), which would be useful for the fabrication of visible-light-excitable deep-blue RTP materials. To be fair, luminescent materials with large Stokes shift can minimize the interference of scattered light from excitation source; this is an advantage of conventional RTP materials. However, in the case of deep-blue RTP materials, large Stokes shift means that high-energy UV sources (which may destabilize organic materials) are required to excite the materials; for instance, in the reported studies, UV lights of short wavelengths such as 310 nm, 280 nm or even shorter are used to switch on the deep-blue RTP property^5,9^. RTP(T_n_) with small Stokes shift would provide a pathway to achieve deep-blue RTP materials that can be excited by visible light or UVA light. Because of the long-lived excited state nature of RTP materials, the interference from excitation source and background fluorescence can be eliminated by time-gated or afterglow mode. Third, the involvement of RTP(T_n_) would endow organic systems with RTP(T_1_) plus RTP(T_n_) dual phosphorescence property. Given that RTP(T_n_) and RTP(T_1_) possess different population mechanisms and very different phosphorescence decay rates, if some specific stimuli have different influence on RTP(T_n_) and RTP(T_1_) emission intensities, the organic systems would give significant RTP(T_n_)/RTP(T_1_) ratiometric response to function as stimuli-responsive RTP materials.

There are very limited examples of the experimental observations of T_n_-S_0_ (*n* ≥ 2) phosphorescence in conventional conditions^26,27^. In one circumstance, when the T_2_ states possess typical ^3^n-π* characters, the *k*_P_ values of T_2_-S_0_ transition can be increased to a large extent to counterbalance the small population of T_2_ states, leading to T_2_-S_0_ phosphorescence^28–30^. The T_2_-S_0_ phosphorescence in the dealyed emission spectra has been found to be much weaker than T_1_-S_0_ phosphorescence in the reported studies; it is challenging to achieve a major T_2_-S_0_ phosphorescence band in the delayed emission spectra due to the fast T_2_-T_1_ internal conversion. In another circumstance, the T_2_ and T_1_ states have small energy gaps so that T_2_ and T_1_ states are in fast equilibrium to exhibit dual phosphorescence behaviors^26,31,32^; the T_2_-S_0_ and T_1_-S_0_ phosphorescence bands showed large overlap and cannot be resolved very clearly. In the reported studies of both circumstances, the T_2_ energy levels are lower than S_1_ states as revealed by steady-state and delayed emission spectra^26,28–32^. In contrast, in computational studies, one may frequently find up-converted S_1_-T_n_ (*n* ≥ 2) transitions and T_1_-T_n_ reverse internal conversion to open forward and reverse ISC channels^11,15,33–35^; such up-converted processes may be not easy to be understood by non-experts because it seems to be thermodynamically unfavorable. Therefore, a direct observation of up-converted RTP(T_n_) with *λ*_P_(T_n_) <*λ*_F_(S_1_) would have significant impact on the straightforward understanding of the behaviors of higher triplet excited states and the up-converted photophysical processes. However, to the best of our knowledge, in conventional conditions, such RTP(T_n_) with *λ*_P_(T_n_) <*λ*_F_(S_1_) have been rarely observed by experimental studies; in a reported study^36^, RTP(T_n_) signals with higher energy levels than S_1_ states were collected by spectroscopic methods but the RTP(T_n_) signals showed short *τ*_P_ < 10 ms and cannot be observed by human eyes upon ceasing excitation source.

Here we report a serendipitous finding of up-converted RTP with *λ*_P_ < *λ*_F_ and *τ*_P_ > 0.1 s upon doping benzophenone-containing difluoroboron β**-**diketonate (BPBF_2_) into phenyl benzoate (PhB) matrices. The BPBF_2_-PhB materials are prepared by rational material design based on dopant-matrix strategy, while the up-converted RTP is from an unexpected observation. The up-converted RTP has been found to originate from T_n_ (*n* ≥ 2) states of BPBF_2_ which show typical ^3^n-π* characters from benzophenone functional groups. Experimental and computational studies show that the BPBF_2_-PhB systems have a strong tendency to undergo intersystem crossing. Upon intersystem crossing from BPBF_2_’s S_1_ states of charge transfer (^1^CT) characters, the formed T_n_ and T_1_ states build T_n_-T_1_ equilibrium via forward and reverse internal conversion. The T_n_ states of ^3^n-π* characters possess large *k*_P_ values that can strongly compete RTP(T_1_) to directly emit RTP(T_n_) which violates Kasha’s rule.

## Results

### Material fabrication and photophysical measurements

**Fig. 1:**
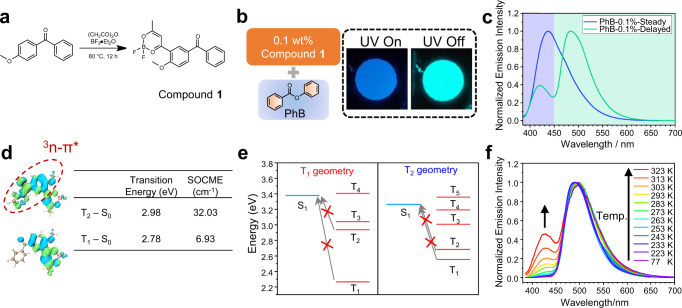
Photophysical properties of compound 1 system. **a** Cascade reaction for the synthesis of compound **1**; **b** Illustration of the material composition and photographs of **1**-PhB-0.1% under a 365 UV lamp and after removal of the UV lamp; **c** Normalized steady (blue line) and delayed (green line, 1 ms delay) emission spectra for **1**-PhB-0.1% powder at room temperature; **d** TD-DFT calculation results of T_1_ and T_2_ states of compound **1** based on optimized S_0_ geometry: the isosurface maps of electron-hole density difference (blue and green isosurfaces correspond to hole and electron distribution, respectively), transition energy values and spin-orbital coupling matrix element (SOCME). TD-DFT calculations were performed on ORCA 5.0.3 program with B3LYP/G functional and def2-TZVP(-f) basis set; **e** Calculated excited state energy levels at the optimized geometry of T_1_ and T_2_ states of compound **1** with B3LYP/G functional and def2-TZVP(-f) basis set; **f** Temperature-dependent delayed emission spectra (1 ms delay) of **1**-PhB-0.1% powder from 77 K to 323 K.

The original purpose of the present study is to fabricate efficient RTP materials in dopant-matrix systems. Since benzophenone systems exhibit strong tendency of intersystem crossing, we synthesized benzophenone-containing difluoroboron β-diketonate compound **1** to serve as luminescent dopants (Fig. 1a). Compound **1** received thorough structural characterization (See Supplementary Information) and photophysical measurements (Supplementary Fig. 1 and Supplementary Table 1). Unlike the reported benzophenone derivatives^12^, compound **1** show insignificant room-temperature organic afterglow in its crystal states (Supplementary Fig. 2).

We use dopant-matrix design strategy to construct organic afterglow materials, where the selection of organic matrix is very important. The selection guideline of organic matrix is based on its role in BF_2_bdk-matrix afterglow system^37^, where BF_2_bdk represents difluoroboron β**-**diketonate compound. (a) Organic matrix should suppress nonradiative decay and oxygen quenching of BF_2_bdk’s T_1_ states, so that crystalline matrix is preferred. (b) In BF_2_bdk-matrix system, organic matrices with carbonyl or ester groups interact with BF_2_bdk’s S_1_ states via dipole-dipole interactions, lower BF_2_bdk’s S_1_ levels (BF_2_bdk’s T_1_ levels are less influenced by matrix’s environment), and thus reduce Δ*E*_ST_ and facilitate intersystem crossing^38^. This dipole effect in enhancing intersystem crossing has also been proved in a recent reported study^39^. Here phenyl benzoate (PhB) and benzophenone (BP) are used to accommodate BPBF_2_ because of their crystalline natures and relatively large dipole moments in the ground states; PhB and BP are two of the most frequently used matrices developed in our lab. By doping 0.1 wt% BPBF_2_ into BP (BP has ground-state dipole moments of 2.96 D as estimated by TD-B3LYP/6-31G(d,p)), the resultant dopant-matrix samples have been found to show insignificant afterglow at room temperature (Supplementary Fig. 3); BP matrix has relatively low T_1_ level (2.76 eV, estimated from phosphorescence maxima) to receive excited state energy from BPBF_2_’s T_1_ states, causing the quenching of organic afterglow in BPBF_2_-BP samples^40,41^. Cyclo olefin polymer (COP) with high T_1_ level but insignificant dipole moment has also been test as organic matrix. The BPBF_2_-COP samples show insignificant room-temperature afterglow (Supplementary Fig. 4).

PhB has ground-state dipole moments of 1.94 D and a high T_1_ level (3.53 eV and 3.46 eV as calculated by TD-B3LYP/6-31G(d,p) and TD-B3LYP/def2-TZVP(-f), respectively). Upon doping 0.1 wt% **1** into PhB matrices, the obtained **1**-PhB-0.1% materials show blue emission under 365 nm UV lamp and exhibit green afterglow with duration of 3 s after switching off UV lamp. Their steady-state emission spectra show 400-600 nm emission bands with *λ*_F_ of 437 nm (Fig. 1c). The delayed emission spectra (1 ms delay) show phosphorescence bands ranging from 450-600 nm with *λ*_P_ of 483 nm and *τ*_P_ of 329 ms, as well as a relatively weak delayed emission band in the higher-energy region with emission lifetime of 303 ms. Surprisingly, this weak delayed emission band has a maximum at 421 nm, which is shorter than the fluorescence maximum (*λ*_F_ = 437 nm). These results are well reproducible (Supplementary Fig. 5). PhB matrices show insignificant phosphorescence upon 365 nm excitation and don’t contribute to the 421 nm delayed emission band (Supplementary Fig. 6). Compound **1** was carefully purified by column chromatography followed by recrystallization in spectroscopic grade n-hexane/dichloromethane for three times. Its high purity was confirmed by HPLC measurement (Supplementary Fig. 7). This can rule out the possibility that the higher-energy delayed emission band of **1**-PhB materials originates from some impurity^17^. In our previous studies^34,35^, the wavelength of delayed emission maxima of TADF-type organic afterglow may be slightly shorter than those in the steady-state emission spectra, which is caused by the aggregation of luminescent dopants; the aggregates cause red shift of steady-state emission spectra but have a less contribution to TADF-type afterglow than monomeric dopants. However, this is not the case in the present study. When the doping concentration is reduced to 0.01% to eliminate the aggregation of luminescent dopants, the higher-energy delayed emission band in the range from 400 to 450 nm with maxima shorter than fluorescence band still exist (Supplementary Fig. 5). TD-DFT calculations of excited state energy levels at the optimized geometry of T_1_ and T_2_ states show that the S_1_ level of compound **1** is much higher than both T_1_ and T_2_ states (Fig. 1e). Given that reverse ISC starts from triplet excited states, these results suggest that reverse ISC and TADF would be insignificant in **1**-PhB system. More discussion to rule out the possibility that the 421 nm delayed emission originate from TADF is attached in Supplementary Discussion. In the reported studies^42^, the axial and equatorial conformation of the T_1_ state of phenothiazine-containing compound at room temperature have been found to exhibit higher-energy (local minimum) and lower-energy (global minimum) phosphorescence bands, respectively. At low temperature, the lower-energy bands decrease while the higher-energy bands still exist^42^. One may reason that the 421 nm and 483 nm bands in the present study originate from local minimum and global minimum of **1**’s T_1_ states, respectively. If this is true, the 421 nm higher-energy band should still exist at low temperature, but here the variable temperature delayed emission spectra of **1**-PhB materials show the decrease and absence of 421 nm band upon lowering temperature (Fig. 1f). Therefore, the conformation-dependent or twist-induced T_1_ level change is not likely to be the case in the present system with dual RTP property. Besides, in a very recent study of BF_2_bdk-matrix system reported by our group^43^, the twisted BF_2_bdk compound showed RTP spectral shift upon conformation change, whereas dual RTP has not been observed. In addition, compound **1** has only one conformation in single crystal structure (Supplementary Fig. 40 and Supplementary Data 1). Moreover, TD-DFT calculations have also been performed to investigate the dependence of T_1_ levels on **1**’s conformation (Supplementary Fig. 8); the conformation is defined by the twisted angle between aromatic donor and dioxaborine acceptor. The excitation energy of **1**’s T_1_ state as a function of the twisted angle shows only one energy minimum (Supplementary Fig. 8). These results and analyses exclude the possibility of twist-induced dual RTP in the present system. We realize that here the higher-energy band at 421 nm may originate from T_n_ (*n* ≥ 2) state of ^3^n-π* characters because of the involvement of benzophenone functional groups. This receives support from TD-DFT calculation (Supplementary Fig. 9). Figure 1d shows the isosurface maps of electron-hole density difference between triplet excited states and ground states of compound **1**. It is found that T_2_ states show typical ^3^n-π* character localized on benzophenone moiety (Fig. 1d). The spin-orbital coupling matrix element (SOCME) value of T_2_ to S_0_ transition has been calculated to be as large as 32.03 cm^−1^. With such a large SOCME, it is understandable that the phosphorescence decay from this T_2_ state should be fast (*k*_P_(T_2_)/*k*_P_(T_1_) on the order of 10^2^~10^3^, *vide infra*), which makes the direct observation of RTP(T_2_) possible.

**Fig. 2:**
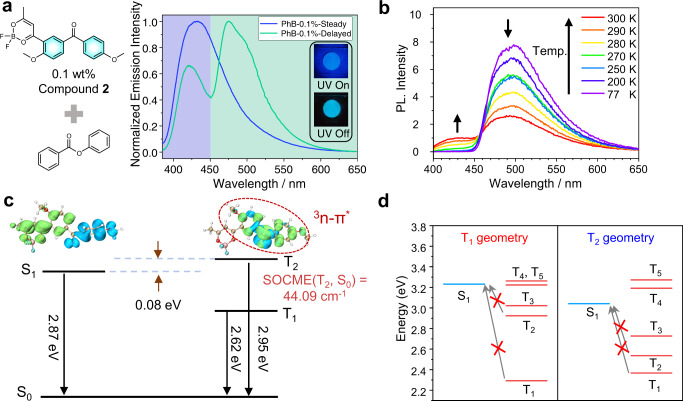
Photophysical properties of compound 2 system. **a** Molecular structure of compound **2**, steady-state and delayed emission spectra (1 ms delay) of **2**-PhB-0.1% powder at room temperature, and fluorescence and afterglow photographs of **2**-PhB-0.1% powder; **b** Temperature-dependent delayed emission spectra (1 ms delay) of **2**-PhB-0.1% powder from 77 K to 300 K; **c** Experimental and TD-DFT calculation results of S_1_, T_1_, T_2_ states of compound **2**: the isosurface maps of electron-hole density difference, SOCME values (at optimized T_2_ geometry) and energy levels estimated from emission maxima. TD-DFT calculations were performed on ORCA 5.0.3 program with B3LYP/G functional and def2-TZVP(-f) basis set; **d** Excited state energy levels at the optimized geometry of T_1_ and T_2_ states of compound **2**, respectively.

To further study the unusual photophysical behaviors of the BPBF_2_-PhB systems, two more BPBF_2_ compounds, **2** and **3**, are synthesized; their structural characterization results and photophysical data are attached in Supplementary Information. Upon doping into PhB matrices, **2**-PhB-0.1% samples show fluorescence band in the range of 400 to 600 nm with *λ*_F_ of 432 nm (2.87 eV) in their steady-state emission spectra (Fig. 2a and Supplementary Fig. 10). The delayed emission spectra of **2**-PhB-0.1% samples at room temperature exhibit two clearly resolved phosphorescence bands with maxima at 421 nm (2.95 eV) and 474 nm (2.62 eV), respectively; again, the wavelength of the emission maxima of the higher-energy bands (421 nm) is shorter than that of the fluorescence bands (*λ*_F_ = 432 nm). TD-DFT calculation reveals that the T_2_ state of compound **2** at optimized T_2_ geometry possesses typical ^3^n-π* character from benzophenone moieties (Fig. 2c). The T_2_ to S_0_ phosphorescence decay at optimized T_2_ geometry has a large SOCME of 44.09 cm^−1^ (Fig. 2c and Supplementary Fig. 11) (*k*_P_(T_2_)/*k*_P_(T_1_) on the order of 10^3^, *vide infra*), which supports the existence of dual RTP behaviors in **2**-PhB systems. Unlike the reported dual RTP systems^26–32^, the energy levels of the emissive T_2_ states (2.95 eV, estimated from the emission maxima) are higher than those of the S_1_ states (2.87 eV, estimated from the fluorescence maxima). Here experimental Δ*E*(S_1_-T_2_) (−0.08 eV) and Δ*E*(S_1_-T_1_) (0.25 eV) for forward ISC are relatively small. The TD-DFT calculated Δ*E*(S_1_-T_2_) (−0.23 eV) and Δ*E*(S_1_-T_1_) (0.02 eV) for forward ISC at the optimized geometry of S_1_ state are also relatively small (Supplementary Table 3). Besides, the S_1_ states have different symmetry from both T_1_ and T_2_ states (Supplementary Fig. 11). According to the energy gap law and the El-Sayed rule, the **2**-PhB system should possess strong tendency to undergo forward ISC. It is noteworthy that, for the reverse ISC where the excited state energy levels are calculated at the optimized geometry of T_1_ and T_2_ states, both T_1_ and T_2_ levels of compound **2** are calculated by TD-DFT to be much lower than the S_1_ state (Fig. 2d). Given that reverse ISC starts from triplet excited states, these suggest that the reverse ISC and subsequent TADF would be unlikely to occur in **2**-PhB system; these theoretical analyses agree with the experimental results where the room-temperature delayed emission spectra of **2**-PhB materials show the absence of TADF signals that can coincide with the 432 nm fluorescence band (Fig. 2a). Variable temperature phosphorescence measurements (1 ms delay) have been performed. Figure 2b displays that T_1_ phosphorescence band in the lower-energy region dominates at 77 K; T_2_ phosphorescence signal in the higher-energy region has not been observed at 77 K. Upon increasing temperature, the T_2_ phosphorescence bands in the range of 400 to 450 nm appear at 270 K and are found to increase with temperature (Fig. 2b). Figure 1f also exhibits the emergence of T_2_ phosphorescence upon increasing temperature in **1**-PhB system. In both of **1**-PhB (Fig. 1f) and 2-PhB systems, the S_1_ to T_2_ ISC and T_1_ to T_2_ reverse internal conversion are up-conversion processes whose speed increase with temperature. Therefore, the variable temperature delayed emission studies suggest that the T_2_ states of ^3^n-π* characters in both **1**-PhB and **2**-PhB systems are populated by thermally activated ISC process from S_1_ states of ^1^CT characters and T_1_ to T_2_ reverse internal conversion.

**Fig. 3:**
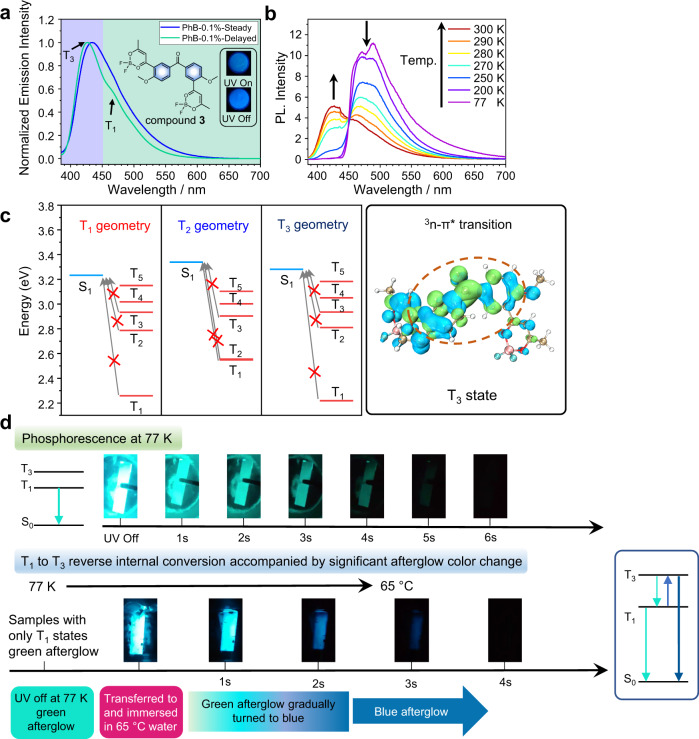
Photophysical properties of compound 3 system. **a** Molecular structure of compound **3**, steady-state and delayed emission spectra (1 ms delay) of **3**-PhB-0.1% powder at room temperature, and fluorescence and afterglow photographs of **3**-PhB-0.1% powder at room temperature; **b** Temperature-dependent delayed emission spectra (1 ms delay) of **3**-PhB-0.1% powder from 77 K to 300 K; **c** Excited state energy levels at the optimized geometry of T_1_, T_2_ and T_3_ states of compound **3**, respectively. The right picture is the isosurface maps of electron-hole density difference of **3**’s T_3_ state calculated at the S_0_ geometry with B3LYP/G functional and def2-TZVP(-f) basis set; **d** Phosphorescence photographs of melt-cast film of **3**-PhB-0.1% at 77 K, photographs showing the phenomenon of afterglow color change when transferring **3**-PhB-0.1% melt-cast film from liquid nitrogen (77 K) to hot water (65 °C) and the proposed mechanism.

In the case of **3**-PhB systems, the up-converted RTP bands at 424 nm (2.92 eV) become the main emission signals in the delayed emission spectra (1 ms delay); the steady-state emission spectra show fluorescence bands at 434 nm (2.86 eV) (Fig. 3a and Supplementary Fig. 12). The **3**-PhB samples at ambient conditions show blue emission under 365 nm UV lamp, and exhibit deep-blue afterglow upon ceasing the 365 nm UV lamp (Fig. 3a). At 77 K, the higher-energy phosphorescence bands at 424 nm (2.92 eV) disappear in the delayed emission spectra, while the lower-energy phosphorescence bands observed at 465 nm (2.67 eV) are assigned as radiative decay of T_1_ states. Variable temperature phosphorescence measurements (1 ms delay) show the enhancement of higher-energy phosphorescence bands at 424 nm upon temperature increase (Fig. 3b). TD-DFT calculations of excited state energy levels at the optimized geometry of T_1_, T_2_, and T_3_ states show that **3**’s S_1_ level is much higher than T_1_, T_2_ and T_3_ states (Fig. 3c), which suggest reverse ISC and TADF should be insignificant in **3**-PhB system. From TD-DFT calculation, it has been found that the T_3_ state of compound **3** has significant n-π* transition character from benzophenone group (Fig. 3c), exhibiting T_3_-S_0_ SOCME of 24.44 cm^−1^ (Supplementary Fig. 13). Besides, *k*_P_(T_3_) has been obtained by theoretical calculation to be on the order of 10^3^~10^4^ s^−1^, much larger than *k*_P_(T_2_) and *k*_P_(T_1_) (*vide infra*). These suggest that the up-converted deep-blue RTP band at 424 nm originates from T_3_ to S_0_ phosphorescence decay; the T_3_ states should be populated from S_1_ to T_3_ ISC and T_1_ to T_3_ reverse internal conversion.

Table 1 summarizes the photophysical data of BPBF_2_-PhB materials under ambient conditions. It is noteworthy that the phosphorescence lifetimes of both T_1_-S_0_ and T_n_-S_0_ are longer than 0.1 s (Table 1 and Supplementary Fig. 14). From *τ*_avg_(T_1_) of several hundred milliseconds, *k*_P_(T_1_) can be estimated to be on the order of 10^0^~10^1^ s^−1^. Unlike the slow T_1_-S_0_ phosphorescence decay, T_n_ to S_0_ phosphorescence process should have much large *k*_P_(T_n_) of around 10^3^ s^−1^ (T_n_ states have significant n-π* transition characters). Therefore, the photophysical pathway of S_1_ to T_n_ ISC and subsequent T_n_ to S_0_ phosphorescence decay cannot directly explain the long *τ*_avg_(T_n_) of several hundred milliseconds. We propose the existence of thermally activated T_1_ to T_n_ reverse internal conversion in BPBF_2_-PhB systems under ambient conditions^26,31,32^. The T_1_-T_n_ equilibrium under ambient conditions can explain the observed long *τ*_avg_(T_n_) of several hundred milliseconds, since the long-lived T_1_ states can serve as reservoir for RTP(T_n_). To verify this, we first prepare a **3**-PhB sample which emit green afterglow at 77 K upon ceasing UV excitation source; in this sample, only T_1_ states exist upon switching off UV excitation (Fig. 3d). After being immediately transferred to and immersed in a 65 °C water bath, the **3**-PhB sample show afterglow color change from green to blue (Fig. 3d). The blue afterglow emission of this sample can be exclusively attributed to T_n_-S_0_ phosphorescence, rather than TADF; in **3**-PhB systems, TADF is insignificant as discussed above (Fig. 3a, b). These observations in Fig. 3d provide very strong evidence on the presence of thermally activated T_1_ to T_n_ reverse internal conversion in the present systems. Such T_1_ to T_n_ reverse internal conversion accompanied by significant afterglow color change visible by naked eyes, which have not been reported in the literature^26,31,32^, can provide straightforward understanding on the triplet excited state dynamics in organic systems. From Table 1, T_n_-T_1_ energy gap can be estimated from phosphorescence maxima to be 0.38 eV, 0.33 eV, and 0.26 eV for **1**-PhB-0.1% (*n* = 2), **2**-PhB-0.1% (*n* = 2) and **3**-PhB-0.1% (*n* = 3) powders, respectively. The decrease of T_n_-T_1_ energy gap can give rise to the increase of the population of T_n_ states, which is in line with the increase of RTP(T_n_)/RTP(T_1_) intensity ratios in these BPBF_2_-PhB-0.1% powder samples under ambient conditions (Fig. 1c,  2a and  3a).

**Table 1:** Photophysical data of BPBF_2_-PhB materials under ambient conditions

Entry	*λ*_F_(S_1_) /nm	*λ*_P_(T_n_) ^a^ /nm	*τ*_avg_(T_n_) ^a^ /ms	*λ*_P_(T_1_) /nm	*τ*_avg_(T_1_) /ms	PLQY
**1**-PhB-0.1% powder	437	421	302.6	483	328.6	16.5%
**2**-PhB-0.1% powder	432	421	278.2	474	297.9	9.4%
**3**-PhB-0.1% powder	434	424	180.1	465	286.2	12.4%

^a^*n* = 2 for **1**-PhB and **2**-PhB materials, *n* = 3 for **3**-PhB material.

### Theoretical investigations

Theoretical calculations on the energy level structures, intersystem crossing, internal conversion, and radiative decay have been performed to further study the intriguing photophysical behaviors in the present system. Table 2 summarizes the excited state energy levels calculated by TD-B3LYP/def2-tzvp(-f) method for the S_0_, S_1_, T_1_, and T_n_ geometries of compounds **1** to **3**; optimization of these geometry was performed at TD-B3LYP/G/6-31G(d,p) level of theory (Supplementary Tables 2–4, Supplementary Tables 12–14). It has been found that, at the optimized geometry of S_1_, the energy levels of S_1_ states are sandwiched between T_1_ and T_n_ states (*n* = 2 for compounds **1** and **2**, *n* = 3 for compound **3**), which agree with experimental observations (Fig. 1c, Fig. 2a, Fig. 3a and Table 1). It is known that, in TD-DFT calculation, the use of hybrid functionals such as B3LYP can reduce self-interaction error but does not eliminate it^44,45^. Range-separated hybrid functionals have been reported to mitigate the systematic error^46,47^, and recent studies showed that the range-separated ωB97X-D functional^48^ exhibits better overall performance on modeling electronically excited states compared to B3LYP functional^49,50^. Here advanced method of TD-ωB97X-D3/def2-tzvp(-f) that may rule out the systematic error coming from B3LYP have also been used to calculate excited state energy levels, which has also been summarized in Table 2, Supplementary Tables 8–10, and Supplementary Tables 15–17; geometry optimization for compound **1–3** was performed at ωB97XD/6-31G(d,p) level of theory. It is found that the energy levels obtained by TD-ωB97X-D3/def2-TZVP(-f) calculation are relatively close to those by experimental observations (Supplementary Tables 2–10 and  11), so we use the results obtained by TD-ωB97X-D3/def2-TZVP(-f) method for the quantum mechanical Fermi’s golden rule (FGR) rate calculation in the present study.

**Table 2:** S_1_, T_1_ and T_n_ excitation energies (eV) of BPBF_2_ compounds calculated at respective optimized geometry (*n* = 2 for compound **1** and **2**, *n* = 3 for compound **3**)

Method	State	S_1_ geometry	T_1_ geometry	T_n_ geometry
TD-B3LYP/G/def2-TZVP(-f)	**Compound 1**			
	**S**_**1**_	2.368	3.379	3.264
	**T**_**1**_	2.297	2.266	2.554
	**T**_**n**_	2.711	2.938	2.683
	**Compound 2**			
	**S**_**1**_	1.981	3.231	3.042
	**T**_**1**_	1.963	2.293	2.367
	**T**_**n**_	2.213	2.924	2.536
	**Compound 3**			
	**S**_**1**_	2.192	3.233	3.281
	**T**_**1**_	2.155	2.258	2.218
	**T**_**n**_	2.517	2.934	2.936
TD-ωB97X-D3/def2-TZVP(-f)	**Compound 1**			
	**S**_**1**_	3.135	3.578	3.480
	**T**_**1**_	2.472	2.307	2.732
	**T**_**n**_	2.898	3.121	2.847
	**Compound 2**			
	**S**_**1**_	3.180	3.856	3.111
	**T**_**1**_	2.612	2.448	2.494
	**T**_**n**_	2.966	3.273	2.969
	**Compound 3**			
	**S**_**1**_	3.167	3.762	3.591
	**T**_**1**_	2.600	2.392	2.897
	**T**_**n**_	3.071	3.258	3.002

For the forward ISC, SOCME values of S_1_ to T_n_ for different geometries have been calculated on ORCA 5.0.3 program with spin-orbit mean-field (SOMF) methods at ωB97X-D3/def2-tzvp(-f) level. Table 3 and Supplementary Tables 15–17 show that most ISC channels have SOCME above 1 cm^−1^ and some ISC channels possess SOCME larger than 10 cm^−1^, which suggest the strong tendency of forward ISC in the system. In the literature, Kaji and coworkers reported the theoretical calculation of quantitative rates of the photophysical processes in benzophenone systems^51,52^. Here the luminescent compounds contain benzophenone functional groups, so we use Kaji’s method to calculate ISC rate constants based on the FGR rate theory (computational details in Supplementary Methods). The calculated ISC rate constants have been summarized in Table 3 and Supplementary Tables 24–26. Besides, the calculations of ISC rate constants via Marcus theory have also been performed^53,54^ (Table 3 and Supplementary Tables 27–29). Both FGR and Marcus theory show that the ISC rate constants of S_1_-to-T_1_ and S_1_-to-T_n_ (*n* = 2 for compounds **1** and **2**, *n* = 3 for compound **3**) are above 10^7^ s^−1^ (Table 3). Given the fluorescence decay of S_1_ states of intramolecular charge transfer nature has rate constants of 10^7^ to 10^8^ s^−1^ (Table 3), such large ISC rate constants would result in relatively high ISC quantum yields in the system. Actually, in the experimental studies, the steady-state emission spectra of **1**-**3** solutions in dichloromethane at 77 K exhibit significant components of phosphorescence signals (Supplementary Fig. 15), which also support the strong tendency of ISC in the present system. For the reverse ISC, from the results obtained by both B3LYP and ωB97X-D3 methods, it is found that, at the optimized geometry of either T_1_ or T_n_ (*n* = 2 for compounds **1** and **2**, *n* = 3 for compound **3**), the T_1_ and T_n_ levels are much lower than S_1_ levels (Table 2). Given that reverse ISC starts from triplet excited states, these results suggest that reverse ISC is not likely to occur. The corresponding rate constants of reverse ISC have also been calculated to show small values (Supplementary Tables 24–29), which can explain the absence of TADF afterglow in the experimental observations.

**Table 3:** Calculated SOCME (cm^−1^), ISC rate (*k*_ISC_, s^−1^), and fluorescence emission rate (*k*_F_, s^−1^) at the optimized S_1_ geometry of **1–3** with ωB97X-D3 functional and def2-TZVP(-f) basis set

	SOCME (cm^−1^)		*k*_ISC_ (s^−1^)		*k*_F_ (s^-1^)
	S_1_ → T_1_	S_1_ → T_n_^a^	S_1_ → T_1_	S_1_ → T_n_^a^	S_1_ → S_0_
1	21.92	26.18	3.70*10^9^ (1.86*10^9^) ^b^	1.50*10^10^ (2.70*10^7^) ^b^	2.19*10^7^
2	11.44	6.68	1.55*10^9^ (3.92*10^10^) ^b^	2.51*10^9^ (5.89*10^9^) ^b^	5.95*10^7^
3	12.85	9.59	1.83*10^9^ (5.04*10^10^) ^b^	2.63*10^9^ (4.84*10^8^) ^b^	7.47*10^7^

^a^*n* = 2 for compound **1** and **2**, *n* = 3 for compound **3**.

^b^Intersystem crossing rate at 300 K calculated by Marcus theory.

Upon forward ISC, we propose that, based on the experimental observations summarized in Table 1 and shown in Fig. 3d, the resultant T_1_ and T_n_ states would build T_1_-T_n_ equilibrium via forward and reverse internal conversion. Kaji’s method^51,52^ has been used to calculate the rate constants of forward internal conversion; frequency analyses are performed at ωB97XD/6-31G(d,p) level of theory (detailed in Supplementary Methods). It is found that forward internal conversion is very fast with rate constant of *k*(T_n_-T_1_) on the order of 10^9^~10^13^ s^−1^ (Table 4). For the reverse internal conversion, the rate constants *k*(T_1_-T_n_) calculated by the Arrhenius-type expression (See Supplementary Equation (14)) are found to be largely underestimated (Table 4). Recent studies of anti-Kasha systems^55–57^ showed that electron-vibrational coupling should be taken into account for the calculation of both *k*(T_n_-T_1_) and *k*(T_1_-T_n_). Accordingly, based on the FCclasses software^58^, *k*(T_n_-T_1_) and *k*(T_1_-T_n_) have been calculated to be 10^10^~10^11^ s^−1^ and 10^8^~10^9^ s^−1^, respectively (Table 4 and Supplementary Table 30). The *k*(T_1_-T_n_)/*k*(T_n_-T_1_) ratios have been calculated to be on the order of 10^−3^~10^−2^ at 300 K (Table 4), much larger than those estimated by the Arrhenius-type expression (Table 4). To investigate the phosphorescence decay of T_1_ and T_n_ states, the corresponding SOCME values, transition dipole moments, and phosphorescence rate constants have been calculated and summarized in Table 5. Calculated at TD-ωB97X-D3/def2-tzvp(-f) level of theory, the SOCME values and transition dipole moments of T_n_-S_0_ phosphorescence decay at T_n_ geometries (*n* = 2 for compounds **1** and **2**, *n* = 3 for compound **3**) have been found to be much larger than those of T_1_-S_0_ phosphorescence decay at T_1_ geometries (Table 5). Phosphorescence rate constants, *k*_P_(T_1_) and *k*_P_(T_n_), have been obtained by FGR rate theory (Table 5). The *k*_P_(T_n_) values of 10^3^~10^4^ s^−1^ at T_n_ geometries (*n* = 2 for compounds **1** and **2**, *n* = 3 for compound **3**) are found to be much larger than *k*_P_(T_1_) values of 10^0^~10^1^ s^−1^ at T_1_ geometries, exhibiting *k*_P_(T_n_)/*k*_P_(T_1_) ratios of 10^2^~10^3^. Given that the relative emission intensity of RTP(T_n_)/RTP(T_1_) is proportional to *k*(T_1_-T_n_)/*k*(T_n_-T_1_) × *k*_P_(T_n_)/*k*_P_(T_1_), the above theoretical calculations support the experimental observation of RTP(T_n_)/RTP(T_1_) dual emission in the delayed spectra.

**Table 4:** Internal conversion rates (*k*_IC_, s^−1^) between T_1_ and T_n_ of **1–3** calculated by different methods (*n* = 2 for compound **1** and **2**, *n* = 3 for compound **3**). Geometries and frequencies are calculated at TD-ωB97XD/6-31G(d,p) level of theory, and energy differences are calculated at TD-ωB97X-D3/def2-TZVP(-f) level of theory

	S_0_ geometry	S_1_ geometry	T_1_ geometry	T_n_ geometry	FCclasses^a^
**Compound 1**					
***k***_**IC**_**(T**_**n**_^**a**^**→T**_**1**_**)**	1.43*10^12^	3.93*10^11^	1.08*10^11^	5.39*10^12^	3.02*10^10^
***k***_**IC**_**(T**_**1**_**→T**_**n**_^**a**^**)**	2.57*10^8^	1.00*10^5^	2.28*10^-3^	6.31*10^10^	2.02*10^8^
**Compound 2**					
***k***_**IC**_**(T**_**n**_^**a**^**→T**_**1**_**)**	2.20*10^10^	1.83*10^10^	3.37*10^9^	1.02*10^10^	2.67*10^11^
***k***_**IC**_**(T**_**1**_**→T**_**n**_^**a**^**)**	8.25*10^4^	2.49*10^4^	4.66*10^-5^	1.07*10^2^	7.30*10^8^
**Compound 3**					
***k***_**IC**_**(T**_**n**_^**a**^**→T**_**1**_**)**	2.61*10^12^	1.18*10^12^	3.48*10^11^	2.37*10^13^	6.50*10^10^
***k***_**IC**_**(T**_**1**_**→T**_**n**_^**a**^**)**	1.28*10^7^	1.44*10^4^	9.85*10^-4^	4.08*10^11^	1.20*10^8^

^a^The rate calculations were performed with FCclasses3^58^. Detailed information can be found in Supplementary Information.

Temperature was set to 300 K

**Table 5:** Calculated SOCME (cm^−1^), transition dipole moments (TDM, a.u.), and phosphorescence emission rate (*k*_p_, s^−1^) of **1–3** based on optimized triplet excited states geometries at the ωB97X-D3/def2-TZVP(-f) level of theory

	SOCME (cm^−1^)		TDM (a. u.)		*k*_P_ (s^−1^)	
	T_1_ → S_0_	T_n_^a^ → S_0_	T_1_ → S_0_	T_n_^a^ → S_0_	T_1_ → S_0_	T_n_^a^ → S_0_
1	0.94	32.61	1.8439*10^−4^	2.8084*10^−3^	2.86	9.69 *10^2^
2	1.20	26.09	3.1097*10^−4^	7.6159*10^−3^	1.06 *10^1^	9.28 *10^3^
3	0.52	36.05	4.1461*10^−4^	3.1489*10^−3^	1.76 *10^1^	1.35 *10^3^

^a^*n* = 2 for compound **1** and **2**, and *n* = 3 for compound **3**.

**Fig. 4:**
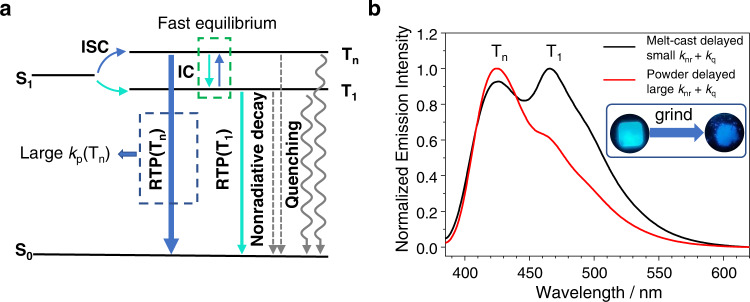
The proposed dual RTP mechanism and RTP(T_n_)/RTP(T_1_) ratiometric response towards mechanical grinding. **a** Jablonski diagram of BPBF_2_-PhB afterglow system; **b** Delayed emission spectra (1 ms delay) of **3**-PhB-0.1% melt-cast sample (black line, with small *k*_nr_ + *k*_q_ value) and powder sample (red line, with large *k*_nr_ + *k*_q_ value) and the phosphorescence photographs after ceasing excitation light source (UV off, 0.1 s) under ambient conditions.

Figure 4a illustrates the photophysical mechanism of the dual RTP systems. Upon excitation, S_1_ states of intramolecular charge transfer character form. Because of the involvement of benzophenone functional groups, different symmetry between S_1_ states and triplet excited states, and relatively small singlet-triplet splitting energies, the system shows strong tendency to undergo intersystem crossing to form T_1_ and T_n_ states. Upon intersystem crossing, the T_1_ and T_n_ states build equilibrium under ambient conditions due to the fast internal conversion and reverse internal conversion facilitated by electron-vibrational coupling. The T_n_ states of n-π* characters from benzophenone groups (*n* = 2 for compounds **1** and **2**, *n* = 3 for compound **3**) have large phosphorescence decay rates and counterbalance the small population of T_n_ states, leading to RTP(T_n_) emission that violate Kasha’s rule. The PhB matrices can provide rigid microenvironment to suppress nonradiative decay (*k*_nr_) and oxygen quenching (*k*_q_) of BPBF_2_’s triplet excited states. The T_1_ states have small phosphorescence rates of 10^0^~10^1^ s^−1^ to show afterglow property with lifetimes of several hundred milliseconds under ambient conditions. Because of the long-lived excited state nature, the T_1_ states also serve as store for the population of T_n_ states via T_1_-T_n_ equilibrium, which is the reason that RTP(T_n_) also possess lifetimes of several hundred milliseconds.

### Material functions

In view of the large difference between *k*_P_(T_1_) and *k*_P_(T_n_), we conceive that the dual RTP materials would give RTP(T_n_)/RTP(T_1_) ratiometric response towards the change of microenvironment of triplet excited states. In the system of **3**-PhB-0.1% under ambient conditions, it is found that powder samples after mechanical grinding have large RTP(T_3_)/RTP(T_1_) intensity ratio than melt-cast samples in the room-temperature phosphorescence spectra (Fig. 4b). Powder samples have large *k*_nr_ + *k*_q_ values than melt-cast samples given other conditions being fixed, because powders have larger surface area exposed to air and sometimes low crystallinity than melt-cast samples. In the reported studies of single-component luminescent systems^59–61^, upon grinding crystalline samples into powders, the emission spectra showed significant change because of the change of aggregation structures in the single-component systems. This is not the case in the present study, since in **3**-PhB-0.1% samples at such a low doping concentration, most of **3** molecules are in monomeric form rather than in aggregation state. The mechano-responsive RTP property of **3**-PhB-0.1% materials derives from the very different *k*_P_ values between T_1_ states and T_3_ states and consequently significant RTP(T_3_)/RTP(T_1_) ratiometric enhancement is observed upon increasing *k*_nr_ + *k*_q_ values.

**Fig. 5:**
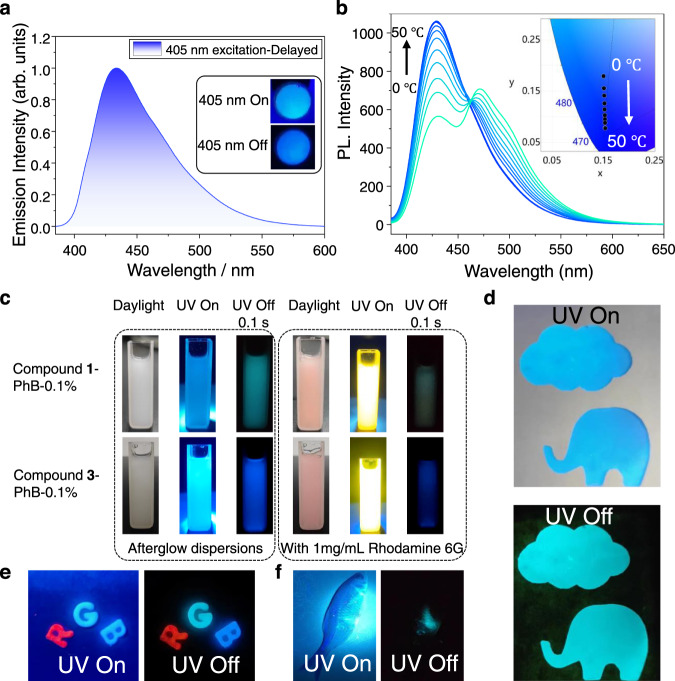
Functionalities of the up-converted RTP materials. **a** Delayed emission spectra (1 ms delay) of **3**-PhB-0.1% powder excited by 405 nm at room temperature. Inset: photographs of the **3**-PhB-0.1% powder, recorded upon switching on (top) and off (bottom) a 405 nm laser; **b** Temperature-dependent delayed emission spectra (1 ms delay) of **3**-PhB-0.1% powder from 0 to 50 °C and CIE diagram; **c** Aqueous afterglow dispersion of BPBF_2_-PhB-0.1% stabilized by Pluronic F127 surfactant; **d** Afterglow pattern of **1**-PhB-0.1% obtained by UV excitation through pre-designed mask; **e** RGB-colored afterglow objects, the letters R, G, and B, were made from NPhRedBF_2_–MeOBP-0.1%^62^, **1**-PhB-0.1% powder, and **3**-PhB-0.1% powder, respectively; **f** Preliminary bioimaging experiments of the aqueous dispersion of **1**-PhB material in fish.

Because of the room-temperature phosphorescence from higher triplet excited states, the present study of **3**-PhB system with *λ*_P_(T_3_) < *λ*_F_(S_1_) and thus small Stokes shift provides a unique method to achieve deep-blue afterglow at room temperature by using UVA or visible light excitation; the deep-blue afterglow under ambient conditions can also be obtained by exciting the **3**-PhB samples at 405 nm (Fig. 5a). In contrast, the reported studies based on conventional RTP mechanism (with large Stokes shift) for deep-blue afterglow requires the use of UV excitation at 310 nm or 280 nm or even shorter wavelengths^5,9^. Since both S_1_ to T_3_ ISC and T_1_ to T_3_ reverse internal conversion are uphill processes and temperature dependent, the **3**-PhB afterglow materials can function as temperature sensor in the range of 0 to 50 °C as shown in Fig. 5b and Supplementary Fig. 16. This temperature-responsive property is originated from the different population mechanisms between T_1_ states and T_3_ states. The BPBF_2_-PhB afterglow materials have been found to be readily melt-cast into large-area films and various shaped objects and processed into aqueous dispersion with the aid of Pluronic F127 surfactants (Fig. 5c). Diverse patterns can be obtained by UV excitation through pre-designed masks (Fig. 5d). Combined with other afterglow materials, RGB-colored afterglow objects can be obtained which can be used to increase security levels of anti-counterfeiting techniques (Fig. 5e). Figure 5c shows the aqueous dispersion of afterglow materials exhibit significant blue emission after ceasing UV lamp, which can eliminate the interference of strong background fluorescence. Preliminary in vivo bioimaging studies has also been performed to display very clean background in the afterglow imaging mode after switching off the excitation source (Fig. 5f).

## Discussion

In summary, the present study reports a serendipitous finding of up-converted RTP with *λ*_P_(T_n_) <*λ*_F_(S_1_) and *τ*_P_ > 0.1 s in BPBF_2_-PhB systems, which has been rarely observed in the reported studies. The involvement of benzophenone functional groups on BPBF_2_ molecules is very important to achieve such up-converted RTP in the dopant-matrix systems since it not only facilitates ISC but also endows T_n_ (*n* ≥ 2) states with n-π* character and large *k*_P_. Given that the energy levels of the T_n_ states are mainly determined by the benzophenone groups, here the use of difluoroboron β**-**diketonate functional groups (with suitable LUMO level and electron-accepting strength) is also very important to result in a proper Δ*E*(T_n_-T_1_) in BPBF_2_ system.

The present study shows that it is still possible to form T_1_-T_n_ equilibrium under ambient conditions in organic systems with Δ*E*(T_n_-T_1_) of around 0.3 eV. Theoretical studies reveal that the electron-vibrational coupling can increase the population of T_n_ states, and the large *k*_P_(T_n_)/*k*_P_(T_1_) ratios can compensate the small population of T_n_ states, leading to anti-Kasha RTP(T_n_) emission. Here the clearly resolved RTP(T_n_) and RTP(T_1_) bands endow the BPBF_2_-PhB materials with stimuli-responsive functions via RTP(T_n_)/RTP(T_1_) ratiometric change towards mechanical force and temperature variation.

The change of RTP(T_n_) emission reflects the change of photophysical processes related to T_n_ states, so the direct observation of RTP(T_n_) facilitates the study of the population, equilibrium, and radiative decay of T_n_ states. The present study would have significant impact on the deep understanding of photophysical behaviors of higher triplet excited states and provide strategies for designing high-performance organic afterglow materials with intriguing properties.

## Methods

### Physical measurements and instrumentation

Nuclear magnetic resonance (NMR) spectra were recorded on a JEOL Fourier-transform NMR spectrometer (400 MHz), including ^1^H NMR, ^13^C NMR, ^19^F NMR, ^11^B NMR. Mass spectra were performed on Agilent Technologies 5973 N and Thermo Fisher Scientific LTQ FT Ultras mass spectrometer. FT-IR spectra were recorded on a Nicolet AVATAR-360 FT-IR spectrophotomerter with a resolution of 4 cm^−1^. Single-crystal X-ray diffraction analysis was performed on a D8 VENTURE SC-XRD instrument. UV-Vis absorption spectra were recorded on a Techcomp UV1050 UV-vis spectrophotometer. Emission spectra were recorded using Hitachi FL-4700 fluorescence spectrometer, Hitachi FL-7000 fluorescence spectrometer and Horiba FluoroLog-3 fluorescence spectrometer. Photoluminescence quantum yield was measured by a Hamamatsu absolute PL quantum yield measurement system based on a standard protocol. Photographs and videos were captured by Xiaomi 11 Ultra camera. Before the capture, samples were irradiated by a 365 nm UV lamp (5 W) for approximately 5 s at a distance of approximately 15 cm.

### Synthesis of luminescent compounds via cascade reaction

In a round bottom flask, boron trifluoride diethyl etherate (2.0 mL, 15.8 mmol) was slowly added into a stirred solution of 4-methoxybenzophenone (425 mg, 2.00 mmol) in acetic anhydride (5.00 mL, 51.4 mmol). The reaction mixture was kept at 80 °C and stirred for 12 h. Then the reaction was quenched by dropwisely adding the reaction mixture into cold water. The precipitates were washed by deionized water for three times and dried under vacuum. The crude product of compound **1** was obtained by column chromatography over silica gel using petroleum ether/dichloromethane (1:2) as eluent. The product of compound **1** was further purified by three times recrystallization in spectroscopic grade dichloromethane/hexane, giving a pale yellow solids with an isolation yield of 22.6% (156 mg). ^1^H NMR (400 MHz, Chloroform-*d*) δ 8.56 (d, *J* = 2.3 Hz, 1H), 8.13 (dd, *J* = 8.7, 2.3 Hz, 1H), 7.82 – 7.71 (m, 2H), 7.60 (t, *J* = 7.4 Hz, 1H), 7.49 (t, *J* = 7.6 Hz, 2H), 7.14 (d, *J* = 8.7 Hz, 1H), 7.00 (s, 1H), 4.07 (s, 3H), 2.40 (s, 3H). ^13^C NMR (101 MHz, Chloroform-*d*) δ 194.40, 193.37, 179.79, 163.34, 138.01, 137.13, 134.37, 132.89, 130.87, 129.89, 128.66, 120.09, 112.18, 102.68, 56.60, 25.08. ^19^F NMR (376 MHz, Chloroform-*d*) δ -138.47 (20.9%), -138.53 (79.1%). ^11^B NMR (128 MHz, Chloroform-*d*) δ -0.10. FT-IR (KBr, cm^-1^): 3167.0, 3079.4, 2954.0, 2845.2, 1651.9, 1604.1, 1536.8, 1467.2, 1437.1, 1367.7, 1340.9, 1307.5, 1269.1, 1254.8, 1168.7, 1100.7, 1048.4, 1010.7, 978.5, 943.5, 876.5, 833.9, 798.8, 736.6, 708.9, 653.0, 632.6, 605.5, 567.2, 513.3, 471.0, 438.8. LRMS, m/z 345.1. HRMS (ESI) m/z found (calcd for C_18_H_16_O_4_^10^BF_2_): 344.1137 (344.1141). The synthetic and purification procedures for compounds **2** and **3** are similar to those of compound **1**, which have been attached in Supplementary Information.

### Preparation of two-component afterglow materials by doping BPBF_2_ compounds into organic matrices

For the preparation of BPBF_2_-PhB-0.1% afterglow materials, 200 μL BPBF_2_ in dichloromethane (1.0 mg/mL) and 200 mg phenylbenzoate (PhB) solids were added into an agate mortar (diameter = 5 cm). After solvent evaporation, the mixture of BPBF_2_ and PhB was heated to 100 °C to form molten mixture. The molten mixture was allowed to stand at room temperature to give solidified melt-cast sample. The powder sample can obtained by grinding melt-cast sample into powder. Afterglow materials with different BPBF_2_ dopants, different doping concentrations, different small organic matrices can be prepared by the procedure above.

## Supplementary information

Supplementary Information

Peer Review File

Description of Additional Supplementary Files

Supplementary Data 1

## Data Availability

The computational input and output files that support the findings of this study are available in Figshare with the identifier [10.6084/m9.figshare.22292959.v4]. The X-ray crystallographic coordinates for structures of compound **1** reported in this study have been deposited at the Cambridge Crystallographic Data Centre (CCDC), under deposition numbers 2156303. These data can be obtained free of charge from The Cambridge Crystallographic Data Centre. The crystallographic data of compound **1** is also provided in the Supplementary Data 1. Other experimental data that support the findings of this study are available from the corresponding author upon request.
