# Supplementary material for: A direct observation of up-converted room-temperature phosphorescence in an anti-Kasha dopant-matrix system
Source: Nat Commun. 2023 Apr 8;14:1987. doi: 10.1038/s41467-023-37662-y (PMC10082826; doi:10.1038/s41467-023-37662-y)
Supplement: Supplementary file 1 — Supplementary Information [file 41467_2023_37662_MOESM1_ESM.pdf]

Supporting Information

A Direct Observation of Up-Converted Room-Temperature Phosphorescence in an Anti-Kasha Dopant-Matrix System

*Jiuyang Li, Xun Li, Guangming Wang, Xuepu Wang, Minjian Wu, Jiahui Liu, and Kaka Zhang**

Key Laboratory of Synthetic and Self-Assembly Chemistry for Organic Functional Molecules, Shanghai Institute of Organic Chemistry, University of Chinese Academy of Sciences, Chinese Academy of Sciences, 345 Lingling Road, Shanghai 200032, People's Republic of China.

*Email: zhangkaka@sioc.ac.cn

Contents

1. Supplementary Figures 3

2. Supplementary Tables 23

3. Supplementary Methods 79

4. Supplementary Discussion 85

5. Supplementary References 86

1. Supplementary Figures

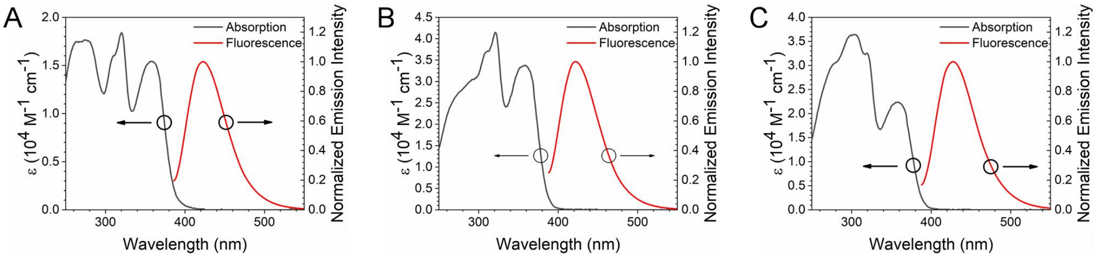

Supplementary Fig. 1 UV-Vis absorption spectra (gray lines) and fluorescence emission spectra (red lines) of $BPBF_2$ in dichloromethane solutions (4.0×10^{-3} mg/mL). A) compound **1**, B) compound **2** and C) compound **3**.

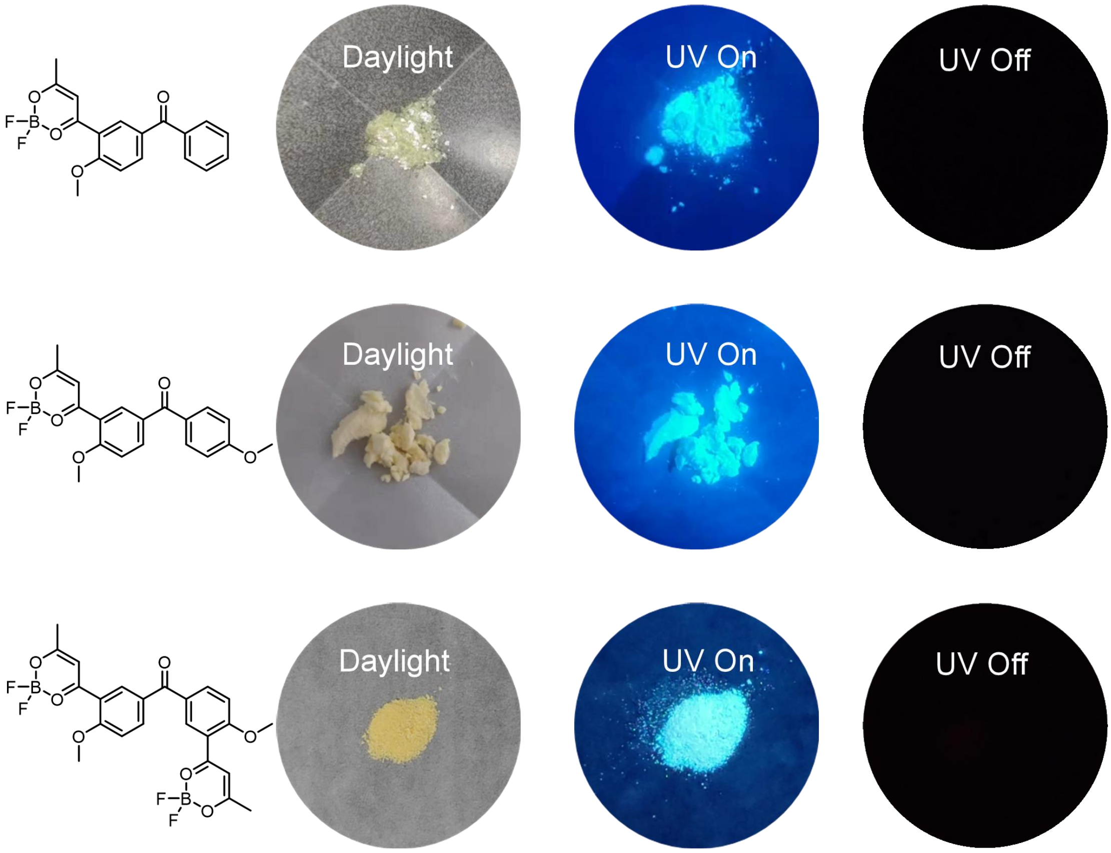

Supplementary Fig. 2 Photographs of $BPBF_2$ compounds under UV lamp and after switching off UV lamp.

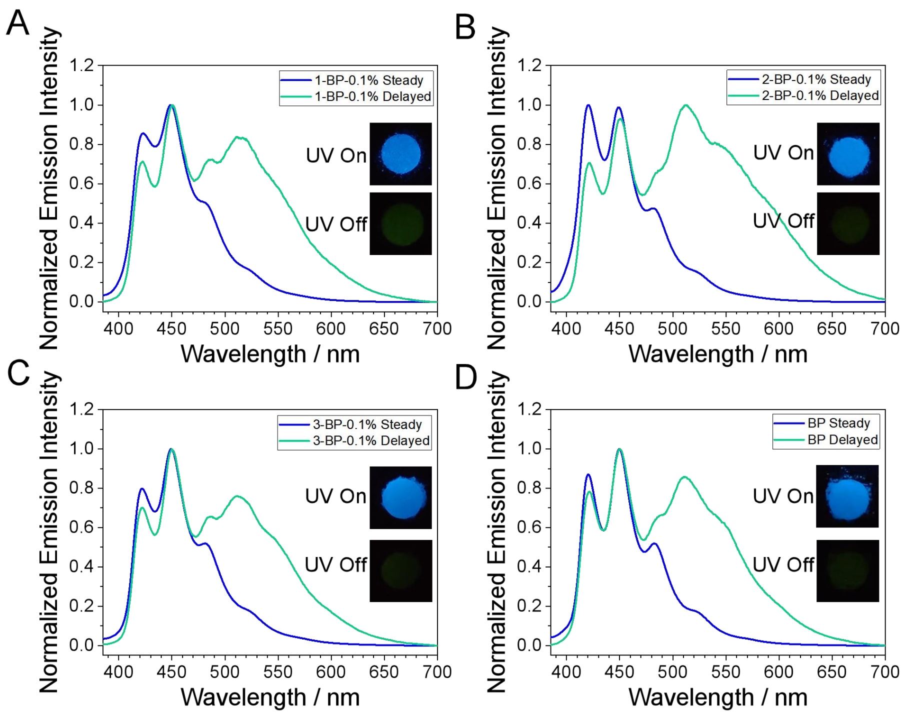

Supplementary Fig. 3 Steady-state and delayed emission (1 ms delay) spectra of A) **1**-BP-0.1% powder, B) **2**-BP-0.1% powder, C) **3**-BP-0.1% powder; D) BP powder. The $BPBF_2$-BP samples show similar spectra to pure BP (benzophenone) at room temperature.

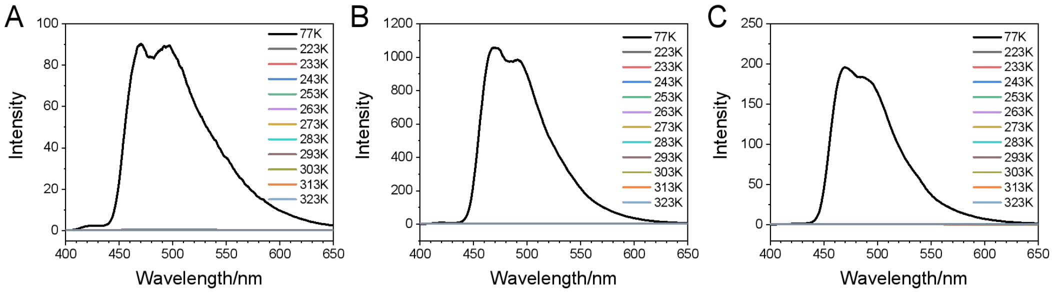

Supplementary Fig. 4 Temperature-dependent delayed emission spectra of A) 1-COP-0.5% sample, B) 2-COP-0.5% sample and C) 3-COP-0.5% sample.

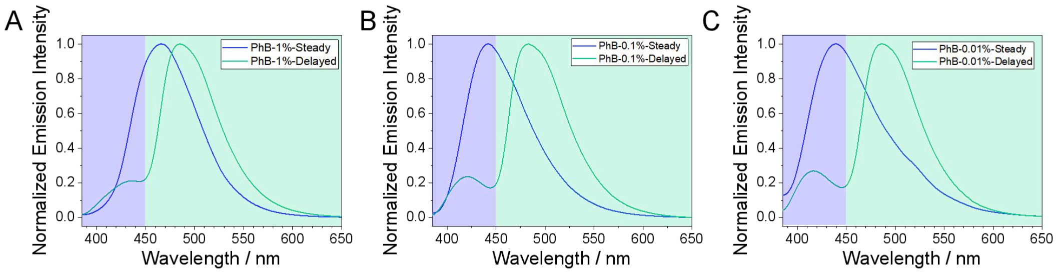

Supplementary Fig. 5 Steady-state and delayed emission spectra of **1**-PhB powders at different doping concentrations. A) 1%, B) 0.1% and C) 0.01%. It is noteworthy that, in the delayed emission spectra of these **1**-PhB samples, the weak delayed emission bands in the range of 400-500 nm have emission maxima with obviously shorter wavelengths than fluorescence maxima in the corresponding steady-state emission spectra.

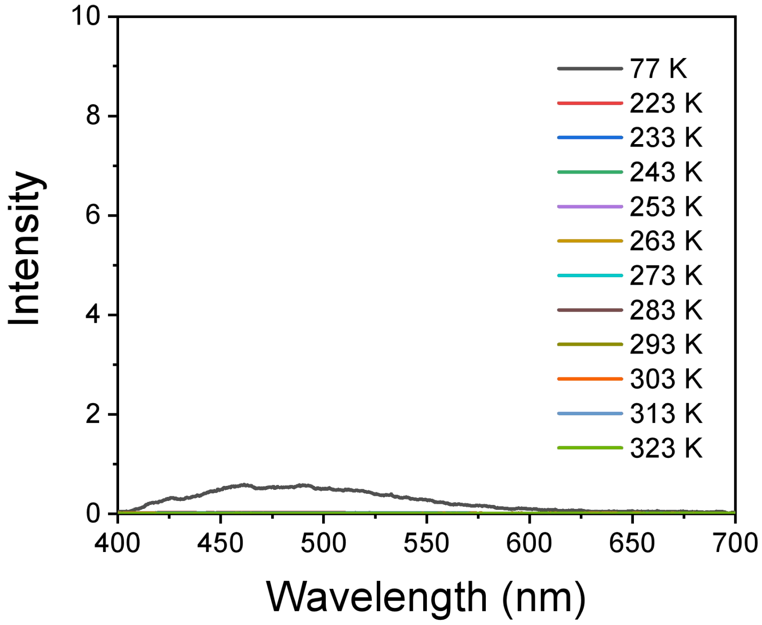

Supplementary Fig. 6 Temperature-dependent delayed emission spectra of pure PhB (1 ms delay).

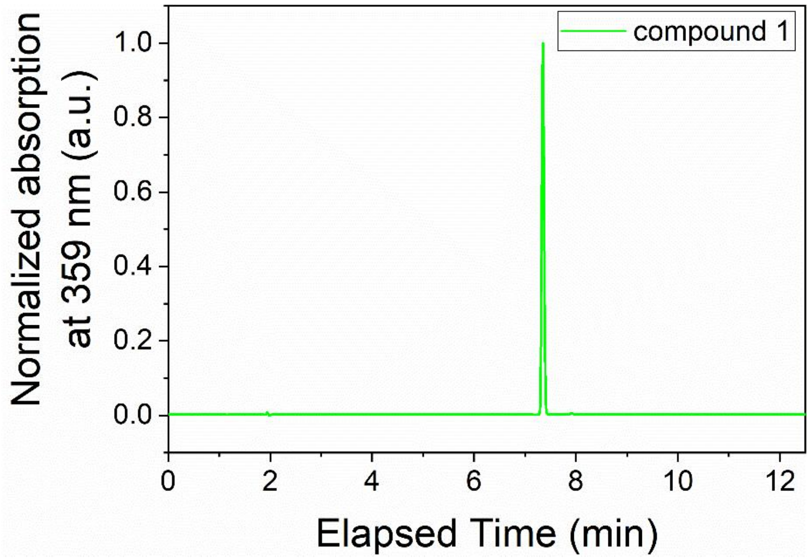

Supplementary Fig. 7 High-performance liquid chromatogram spectra of compound **1.**

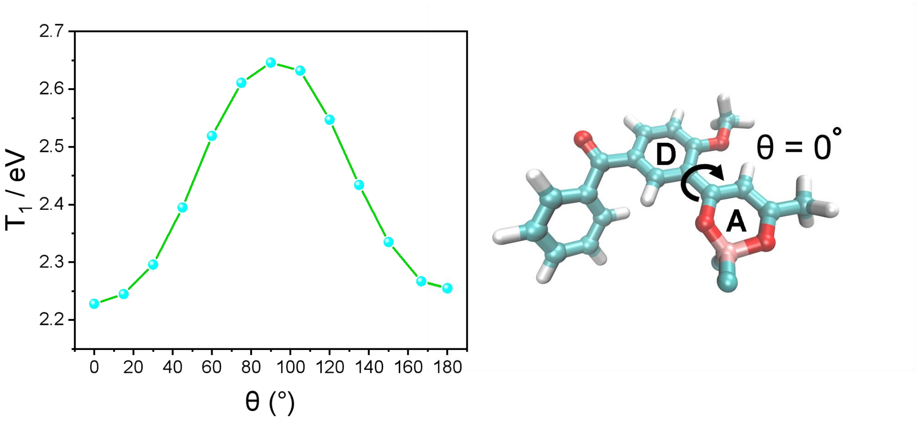

Supplementary Fig. 8 T_1 excitation energy of compound **1** as a function of twisted angle (θ) between aromatic donor ("D" ring) and dioxaborine acceptor ("A" ring) calculated by TD-DFT with B3LYP/G functional and def2-TZVP(-f) basis set.

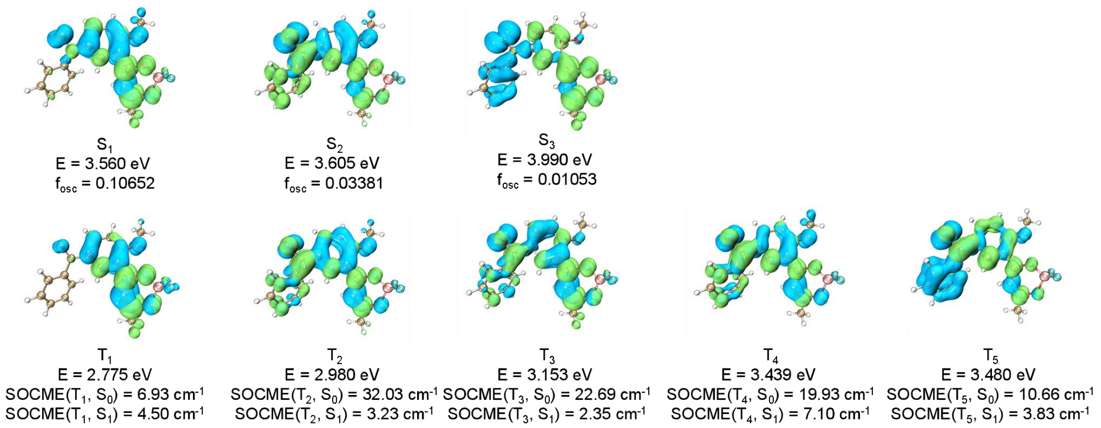

Supplementary Fig. 9 Isosurface maps of electron–hole density difference of the singlet and triplet excited states of compound **1**, where blue and green isosurfaces correspond to hole and electron distributions, respectively, and excitation energies, oscillator strengths and spin–orbit coupling matrix element (SOCME) values. TD-DFT were carried out on ORCA 5.0.3 program with B3LYP/G functional and def2-TZVP(-f) basis set based on optimized S_0 geometry.

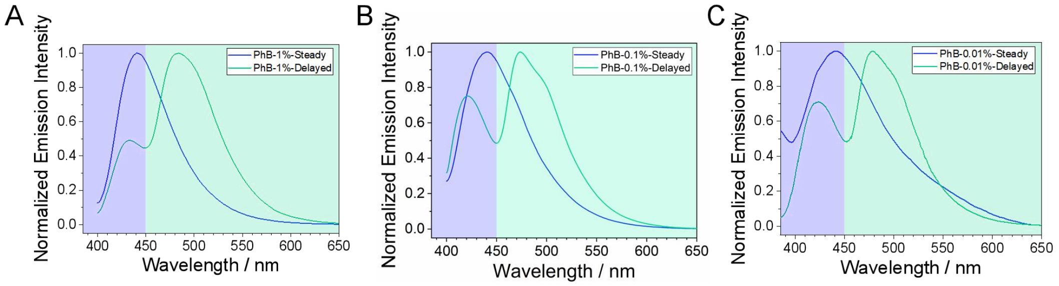

Supplementary Fig. 10 Steady-state and delayed emission spectra of compound **2**-PhB powders at different doping concentrations. A) 1%, B) 0.1% and C) 0.01%.

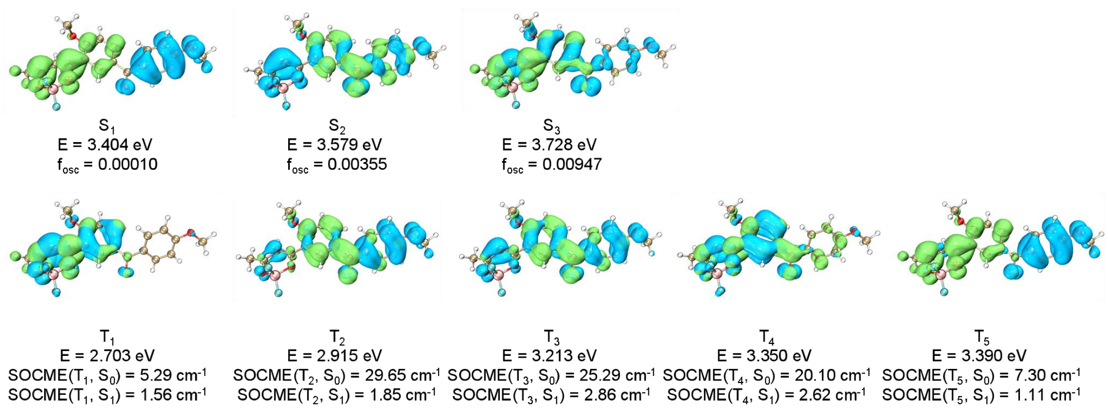

Supplementary Fig. 11 Isosurface maps of electron–hole density difference of the singlet and triplet excited states of compound **2**, where blue and green isosurfaces correspond to hole and electron distributions, respectively, and excitation energies, oscillator strengths and spin–orbit coupling matrix element (SOCME) values. TD-DFT were carried out on ORCA 5.0.3 program with B3LYP/G functional and def2-TZVP(-f) basis set based on optimized S_0 geometry.

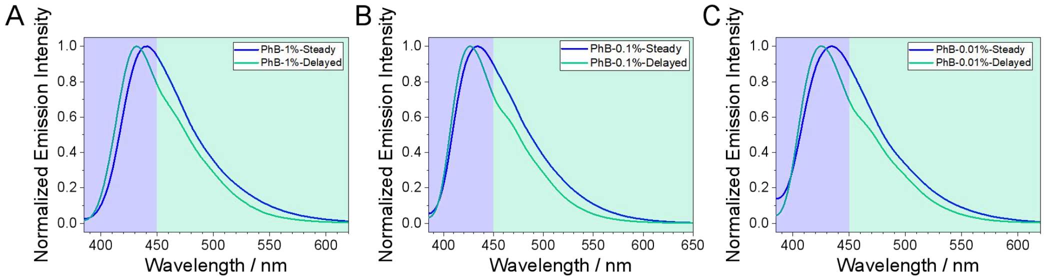

Supplementary Fig. 12 Steady-state and delayed emission spectra of compound **3**-PhB powders at different doping concentrations. A) 1%, B) 0.1% and C) 0.01%.

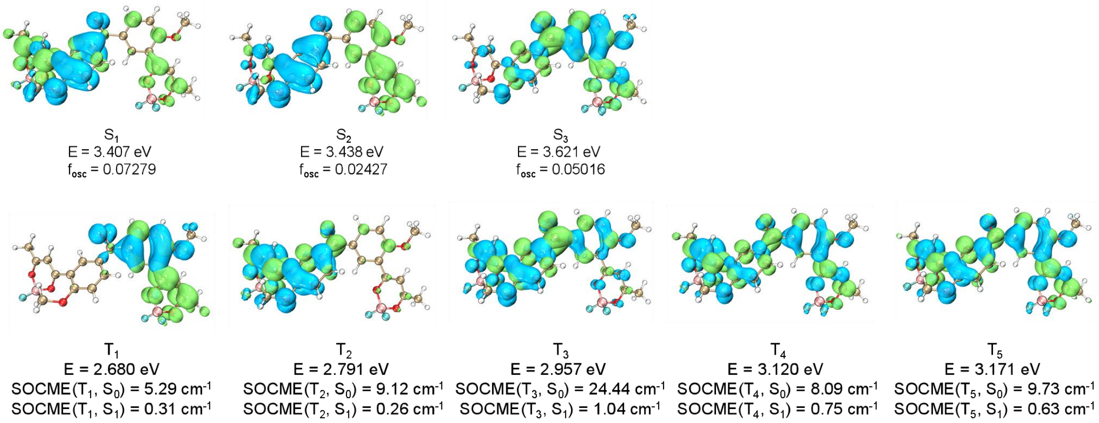

Supplementary Fig. 13 Isosurface maps of electron–hole density difference of the singlet and triplet excited states of compound **3**, where blue and green isosurfaces correspond to hole and electron distributions, respectively, and excitation energies, oscillator strengths and spin–orbit coupling matrix element (SOCME) values. TD-DFT were carried out on ORCA 5.0.3 program with B3LYP/G functional and def2-TZVP(-f) basis set based on optimized S_0 geometry.

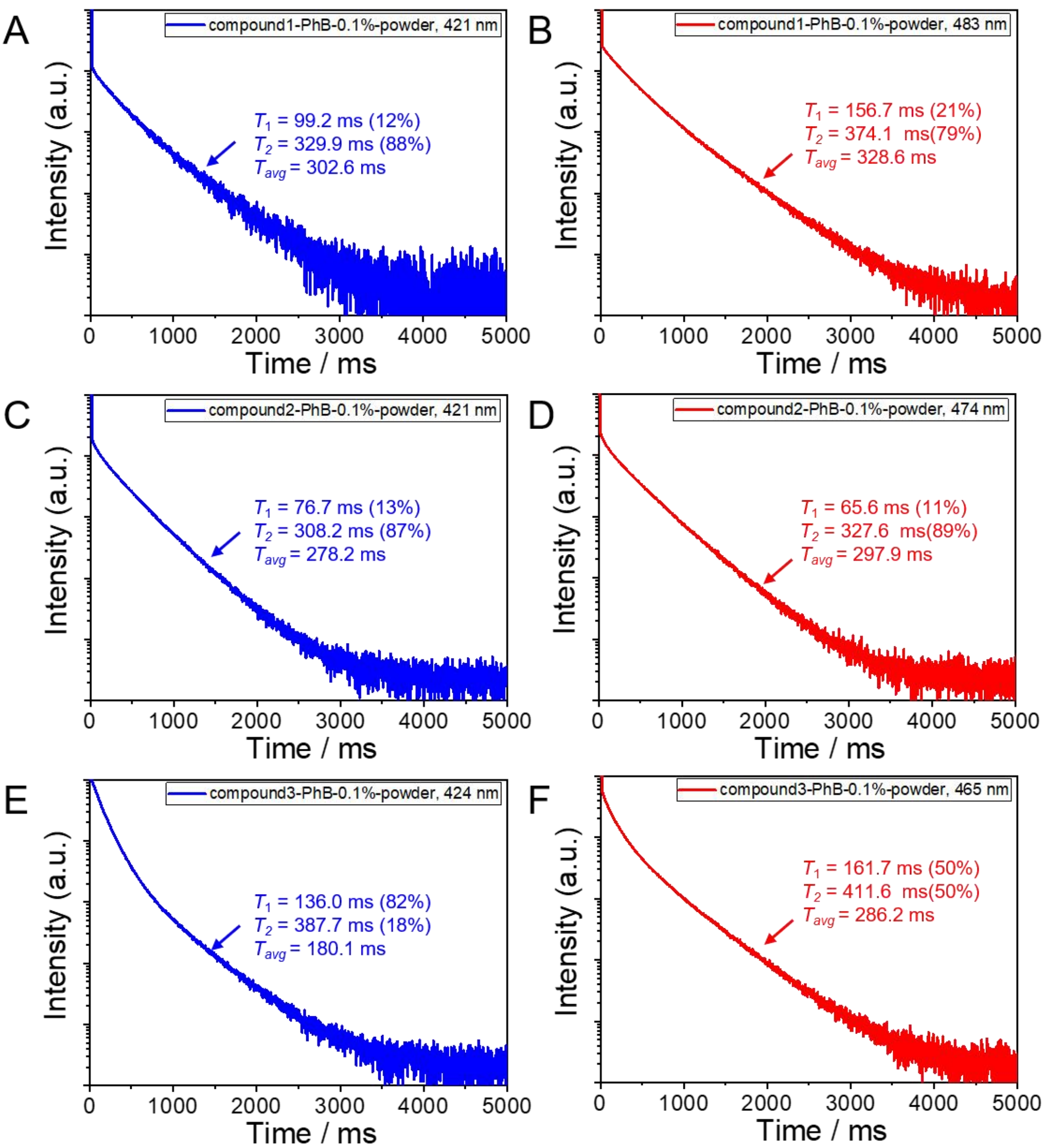

Supplementary Fig. 14 Room-temperature emission decay of $BPBF_2$-PhB-0.1% materials. A) compound **1**-PhB-0.1%-powder measured at 421 nm; B) compound **1**-PhB-0.1%-powder measured at 483 nm; C) compound **2**-PhB-0.1%-powder measured at 421 nm; D) compound **2**-PhB-0.1%-powder measured at 474 nm; E) compound **3**-PhB-0.1%-powder measured at 424 nm; F) compound **3**-PhB-0.1%-powder measured at 465 nm.

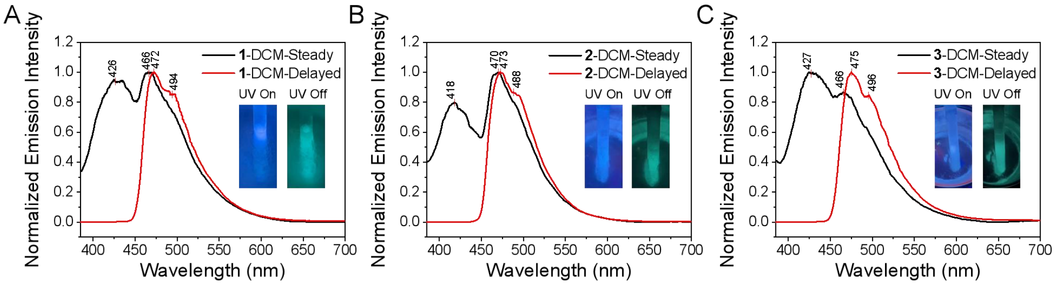

Supplementary Fig. 15 Steady emission spectra (black lines) and delayed emission spectra (1ms delay, red lines) of $BPBF_2$ in frozen dichloromethane at 77 K. A) compound **1**, B) compound **2** and C) compound **3**.

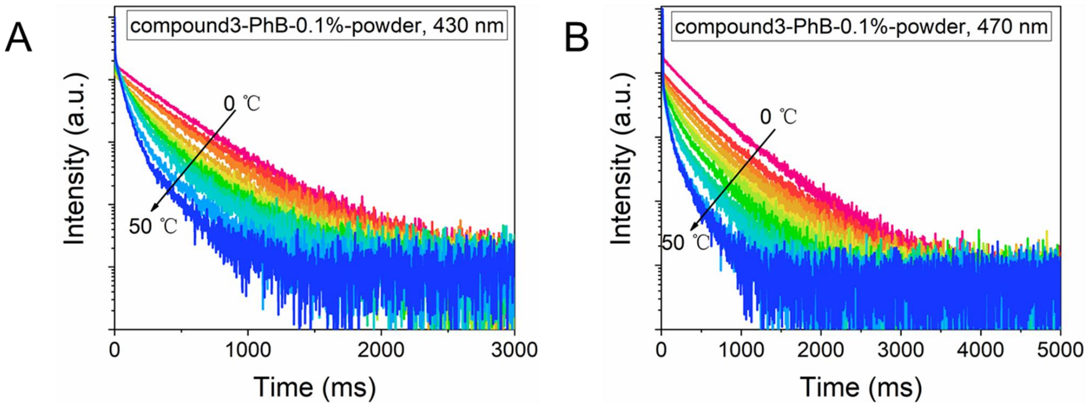

Supplementary Fig. 16 Phosphorescence emission decay curves of compound **3**-PhB-0.1%-powder measured at A) 430 nm and B) 470 nm from 0-50 °C.

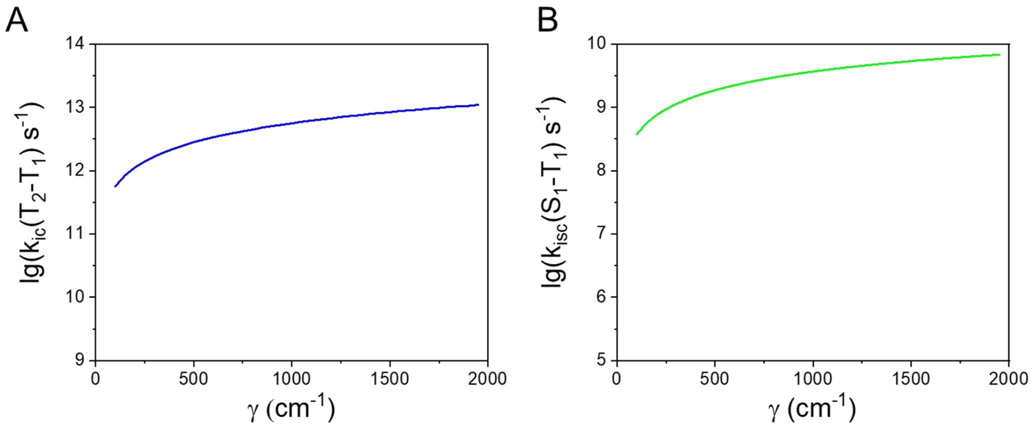

Supplementary Fig. 17 A) Logarithmic value of internal conversion rate (s^{-1}) from T_2 to T_1 of compound **1** against different γ value (cm^{-1}) at T_2 geometry; B) Logarithmic value of intersystem crossing rate (s^{-1}) from S_1 to T_1 of compound **1** against different γ value (cm^{-1}) at S_1 geometry. The γ value has little effect to both of the rate constants (small change in order of magnitude).

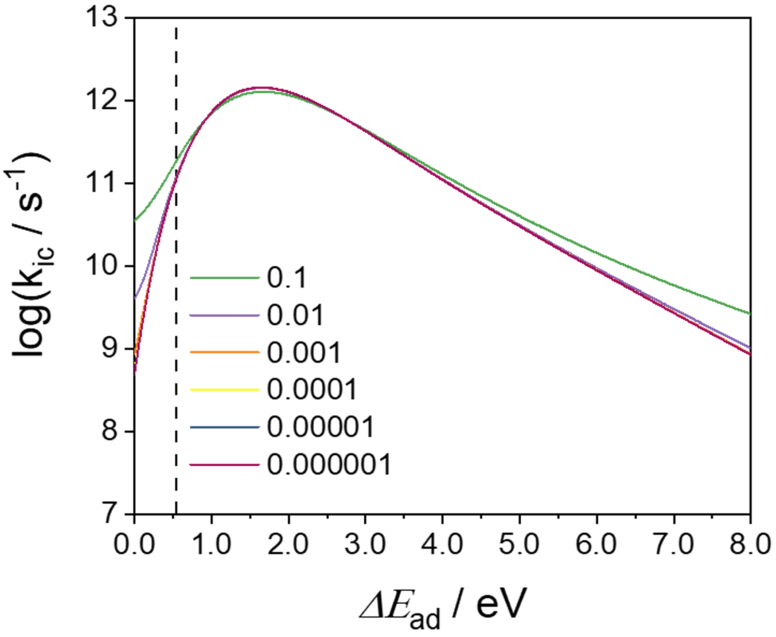

Supplementary Fig. 18. Log(k_{ic}) as a function of the adiabatic energy difference (ΔE_{ad}) for the internal conversion from T_2 to T_1 state of compound **1**, using different HWHMs (0.000001 to 0.1 eV).

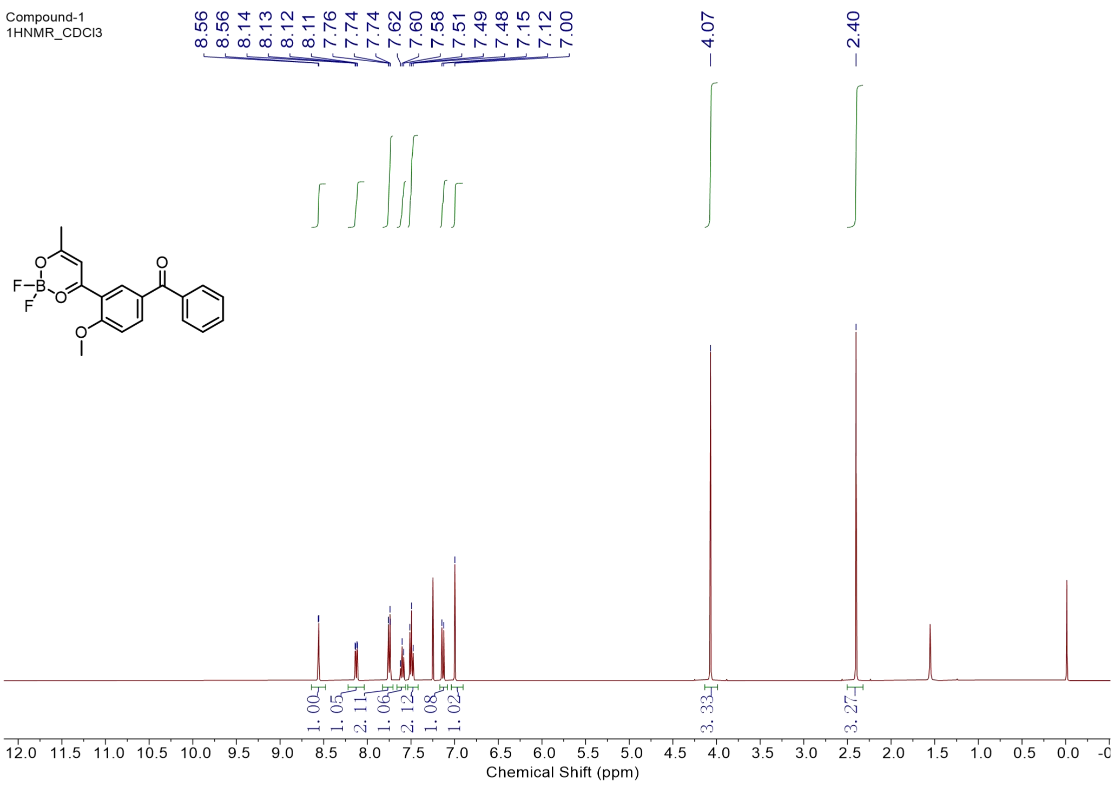

Supplementary Fig. 19 ^{1}H NMR spectrum of compound **1** in $CDCl_3$.

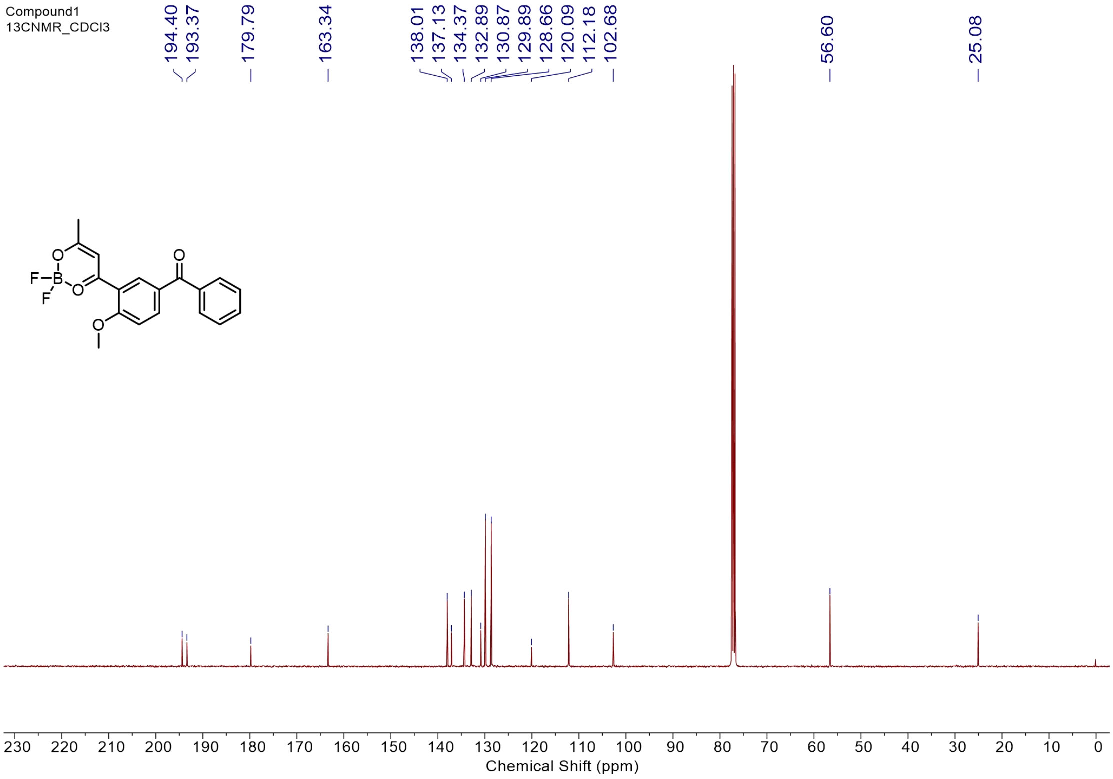

Supplementary Fig. 20 $^{13}C\{^{1}H\}$ NMR spectrum of compound **1** in $CDCl_3$.

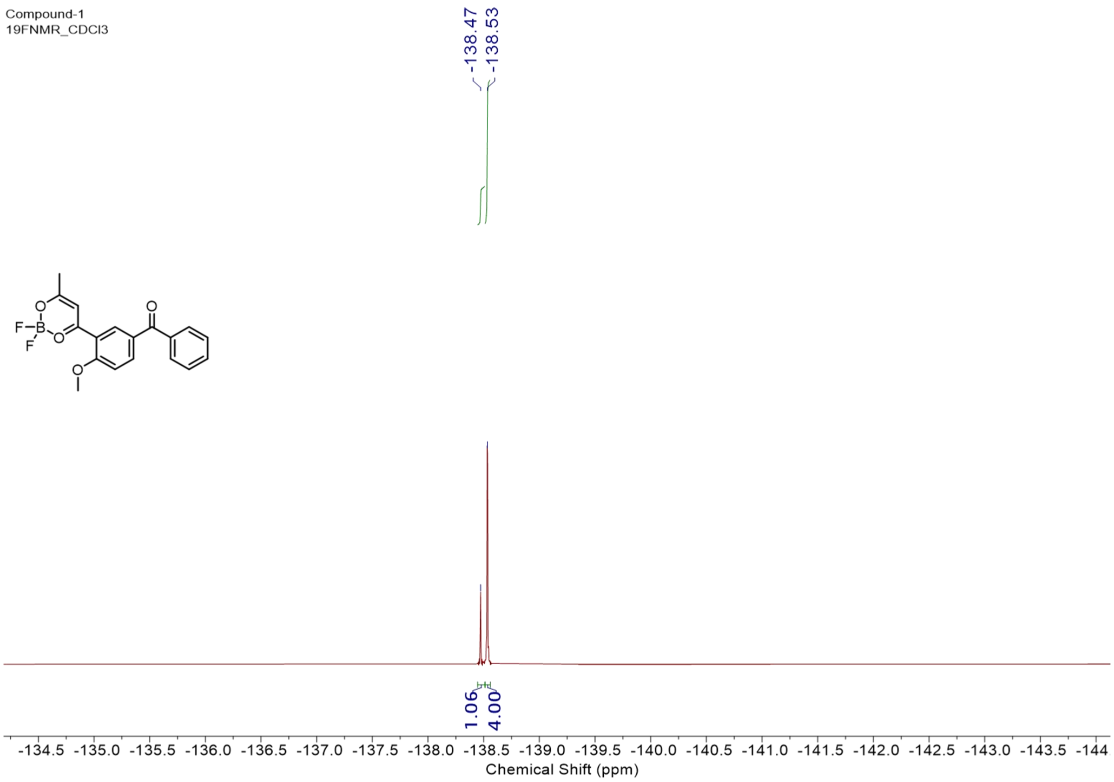

Supplementary Fig. 21 $^{19}F\{^{1}H\}$ NMR spectrum of compound **1** in $CDCl_3$.

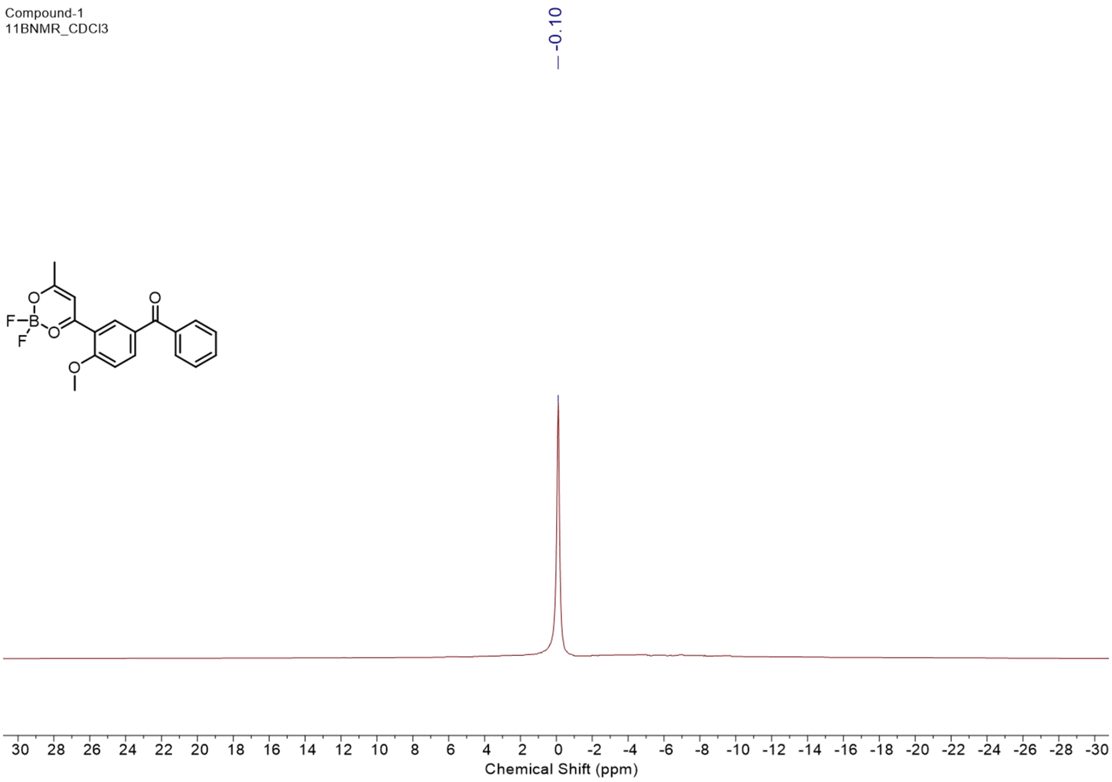

Supplementary Fig. 22 $^{11}B\{^{1}H\}$ NMR spectrum of compound **1** in $CDCl_3$.

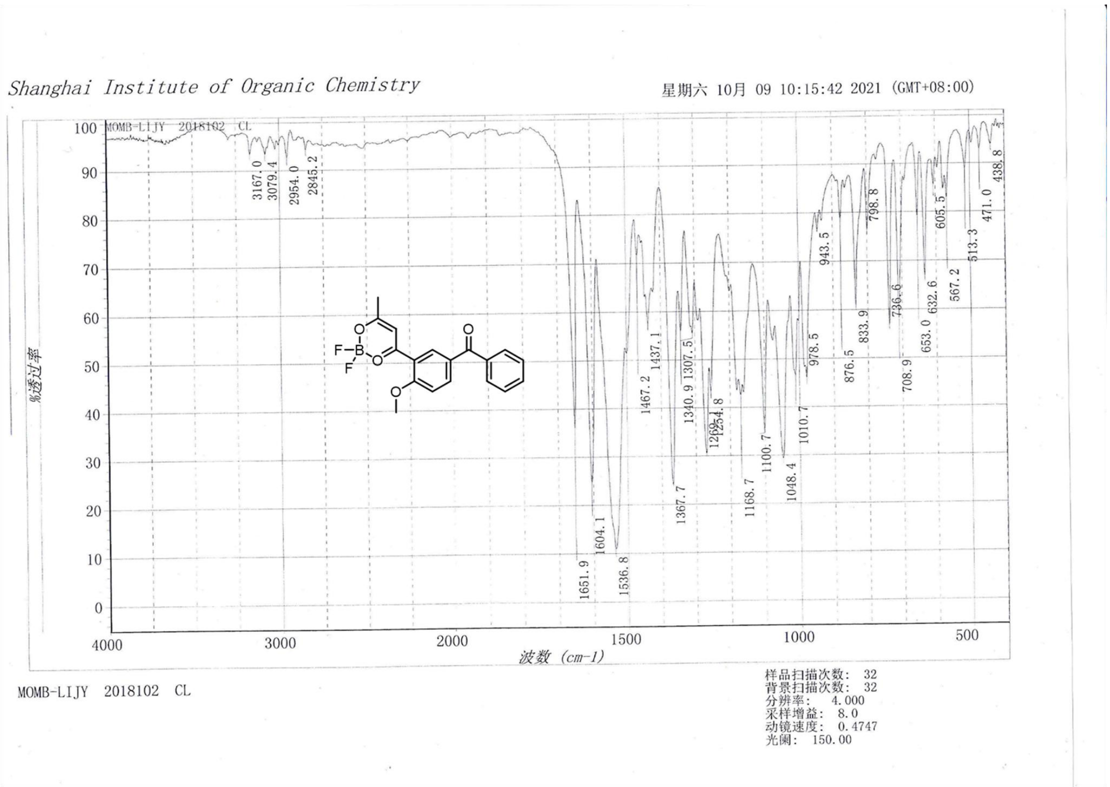

Supplementary Fig. 23 FT-IR spectrum of compound **1**.

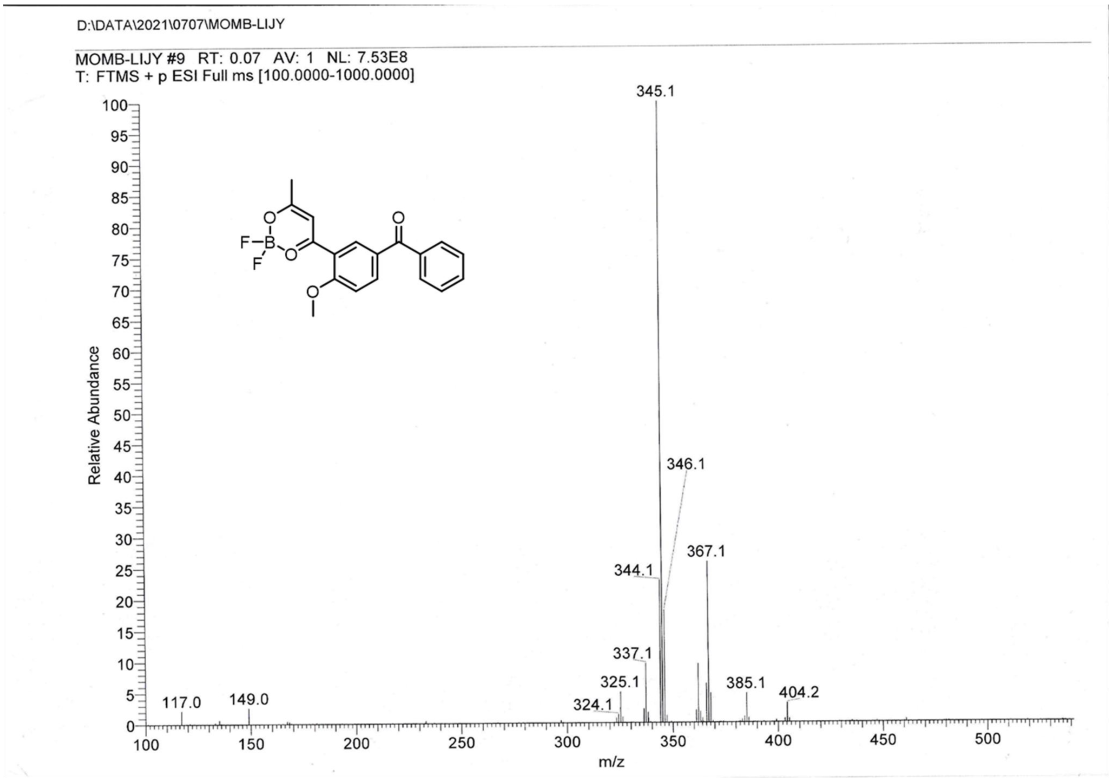

Supplementary Fig. 24 LRMS spectrum of compound **1**.

National Center for Organic Mass Spectrometry in Shanghai
Shanghai Institute of Organic Chemistry
Chinese Academic of Sciences
High Resolution ESI-MS REPORT

Instrument: Thermo Scientific Q Exactive HF Orbitrap-FTMS

Card Serial Number: E211705

Sample Serial Number: MOMB-LIJY

Operator: Songw Date: 2021/07/07

Operation Mode: ESI Positive Ion Mode

Elemental composition search on mass 344.1137

m/z= 339.1137-349.1137

m/z	Theo. Mass	Delta (ppm)	RDB equiv.	Composition
344.1137	344.1141	-0.91	10.5	$C_{18}H_{16}O_4{}^{10}BF_2$

Supplementary Fig. 25 HRMS results of compound **1**.

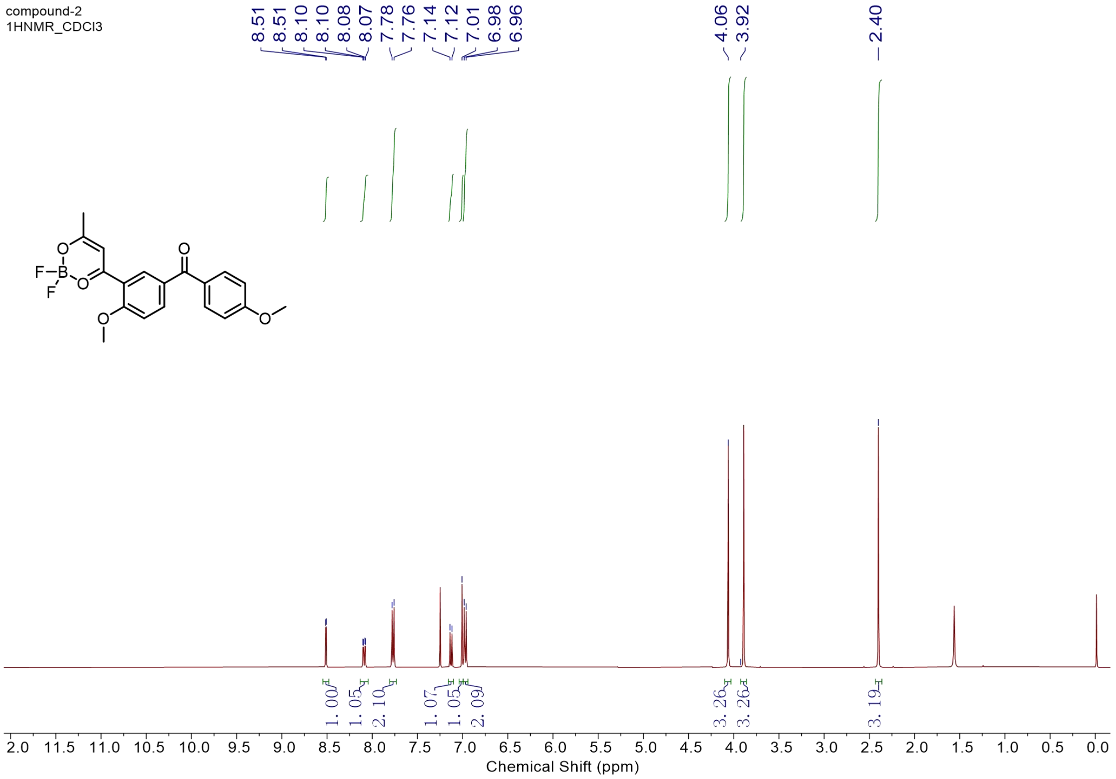

Supplementary Fig. 26 ^{1}H NMR spectrum of compound **2** in $CDCl_3$.

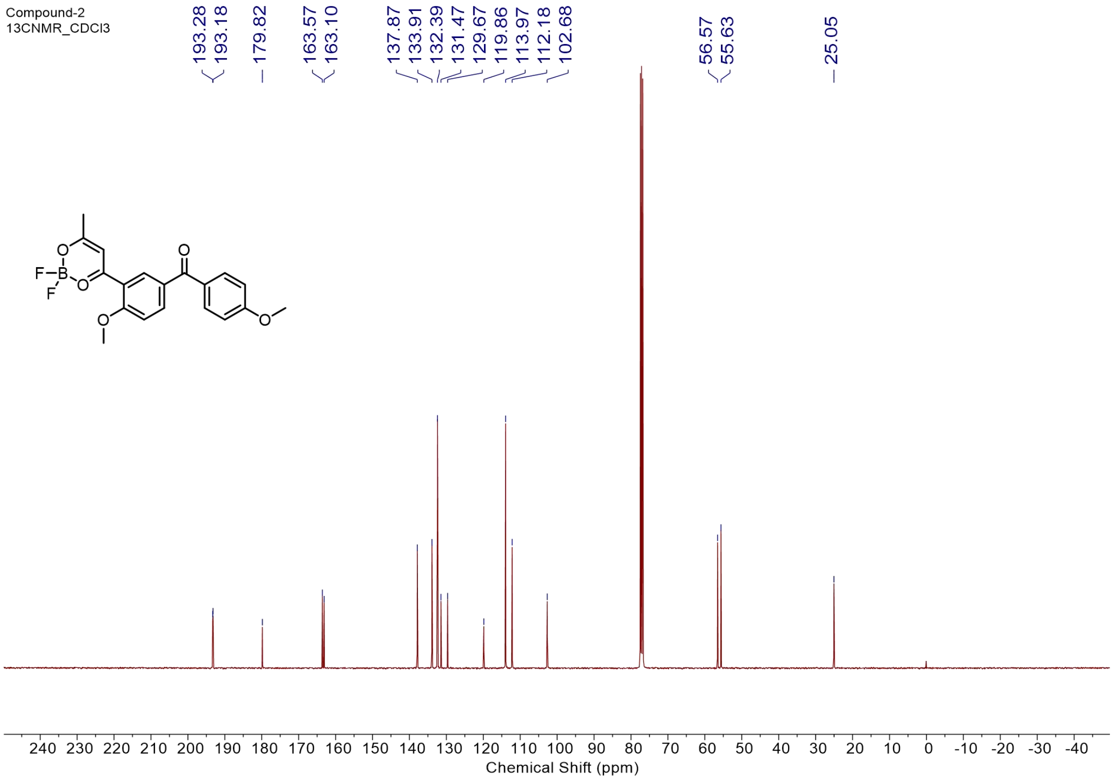

Supplementary Fig. 27 $^{13}C\{^1H\}$ NMR spectrum of compound **2** in $CDCl_3$.

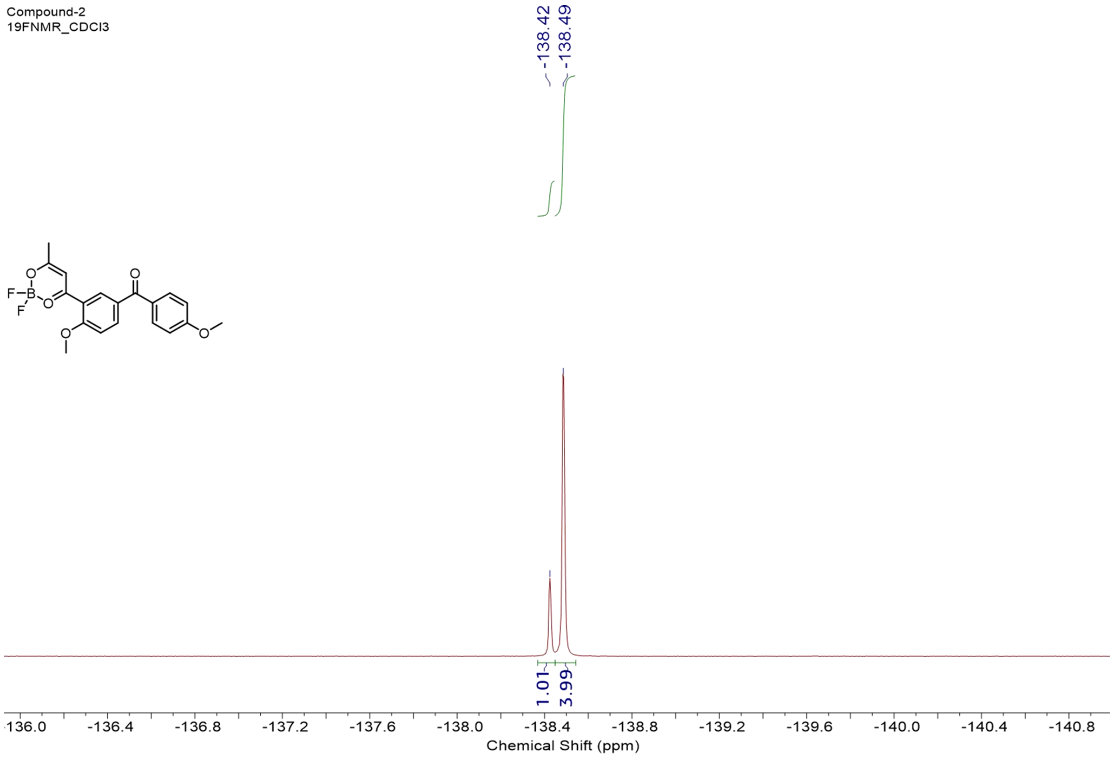

Supplementary Fig. 28 $^{19}F\{^1H\}$ NMR spectrum of compound **2** in $CDCl_3$.

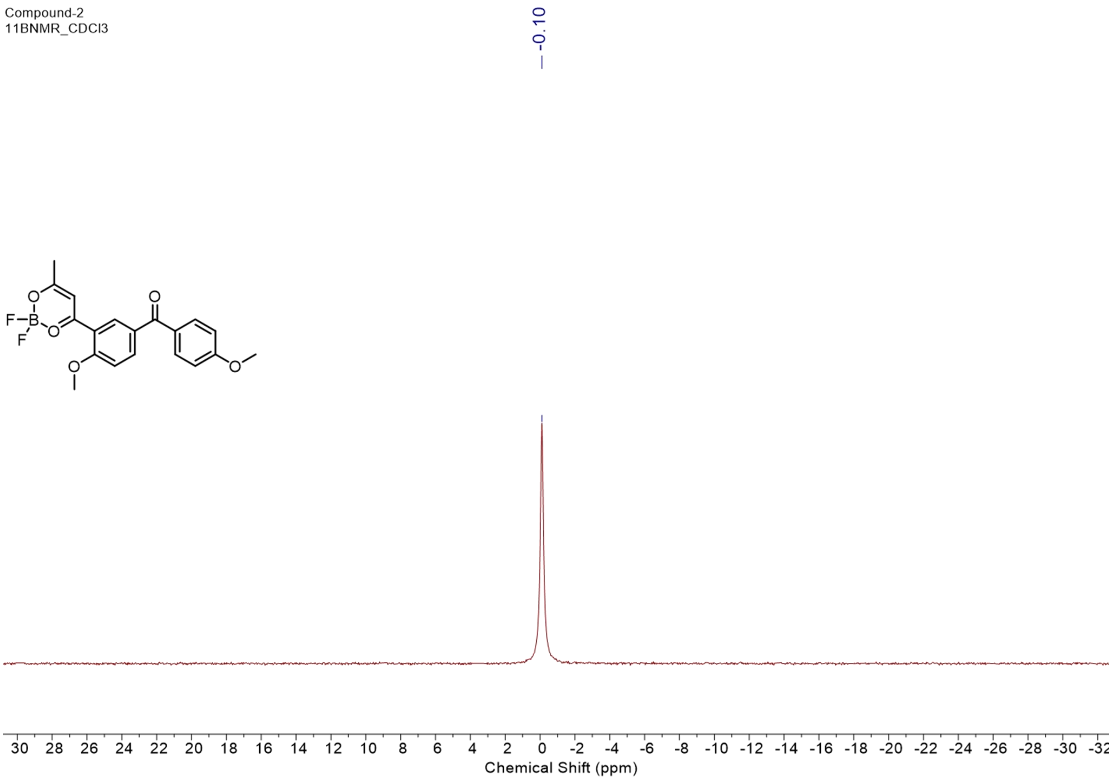

Supplementary Fig. 29 $^{11}B\{^{1}H\}$ NMR spectrum of compound **2** in $CDCl_3$.

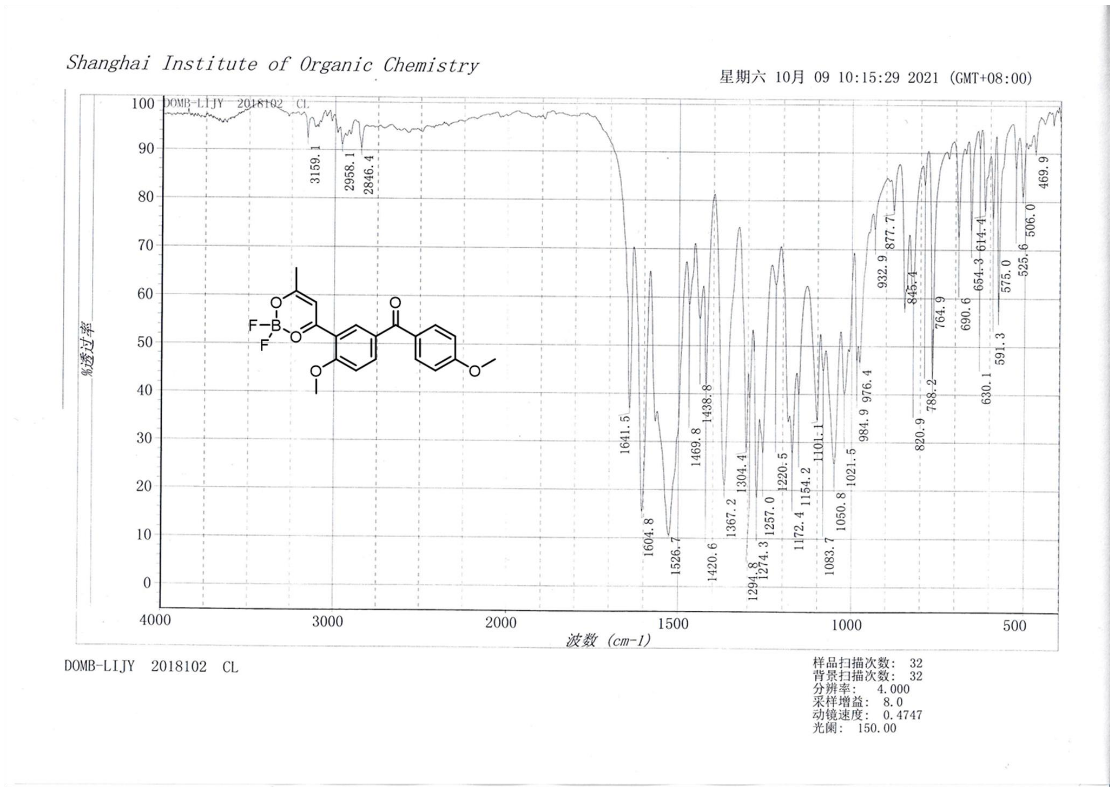

Supplementary Fig. 30 FT-IR spectrum of compound **2**.

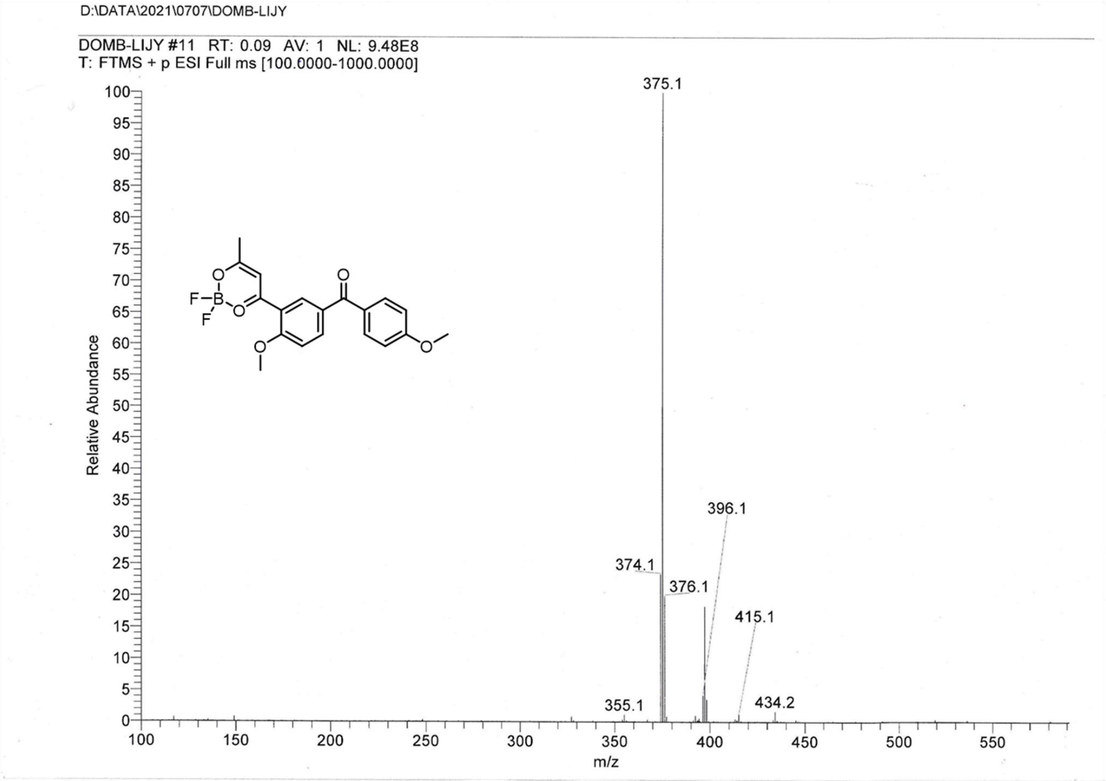

Supplementary Fig. 31 LRMS spectrum of compound **2**.

National Center for Organic Mass Spectrometry in Shanghai
Shanghai Institute of Organic Chemistry
Chinese Academic of Sciences
High Resolution ESI-MS REPORT

Instrument: Thermo Scientific Q Exactive HF Orbitrap-FTMS

Card Serial Number: E211706

Sample Serial Number: DOMB-LIJY

Operator: Songw Date: 2021/07/07

Operation Mode: ESI Positive Ion Mode

Elemental composition search on mass 374.1243

m/z= 369.1243-379.1243

m/z	Theo. Mass	Delta (ppm)	RDB equiv.	Composition
374.1243	374.1246	-0.77	10.5	$C_{19}H_{18}O_5{}^{10}BF_2$

Supplementary Fig. 32 HRMS spectrum of compound 2.

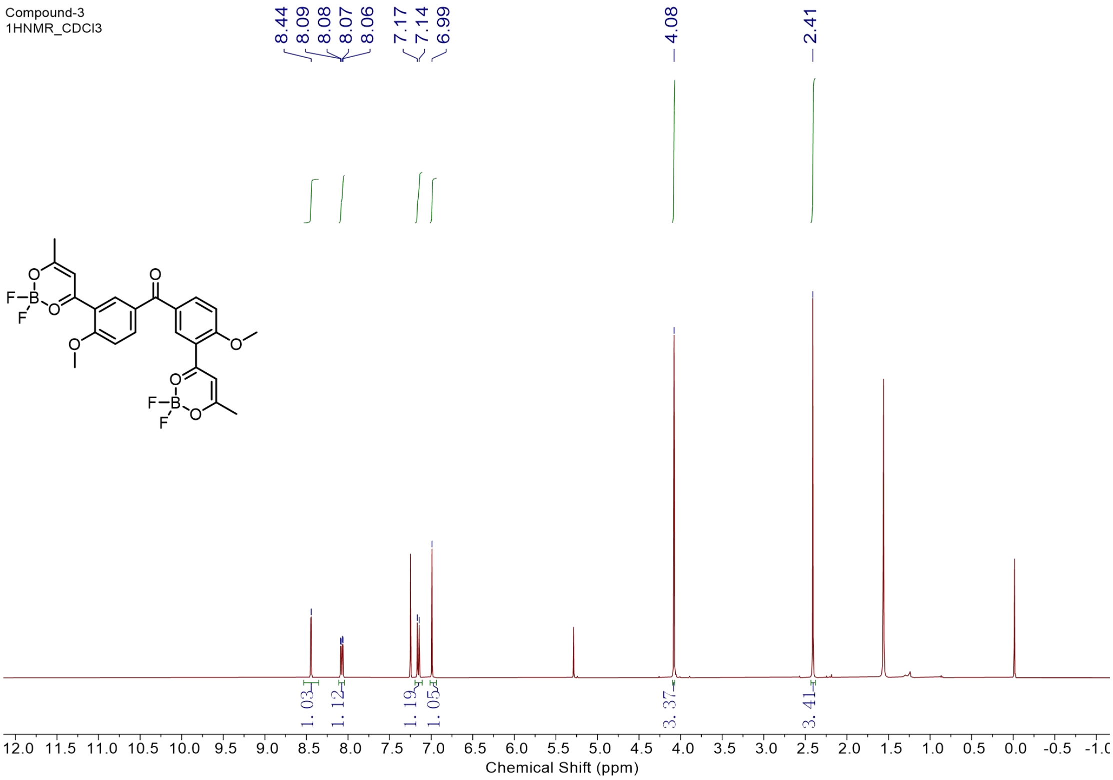

Supplementary Fig. 33 ^{1}H NMR spectrum of compound **3** in $CDCl_3$.

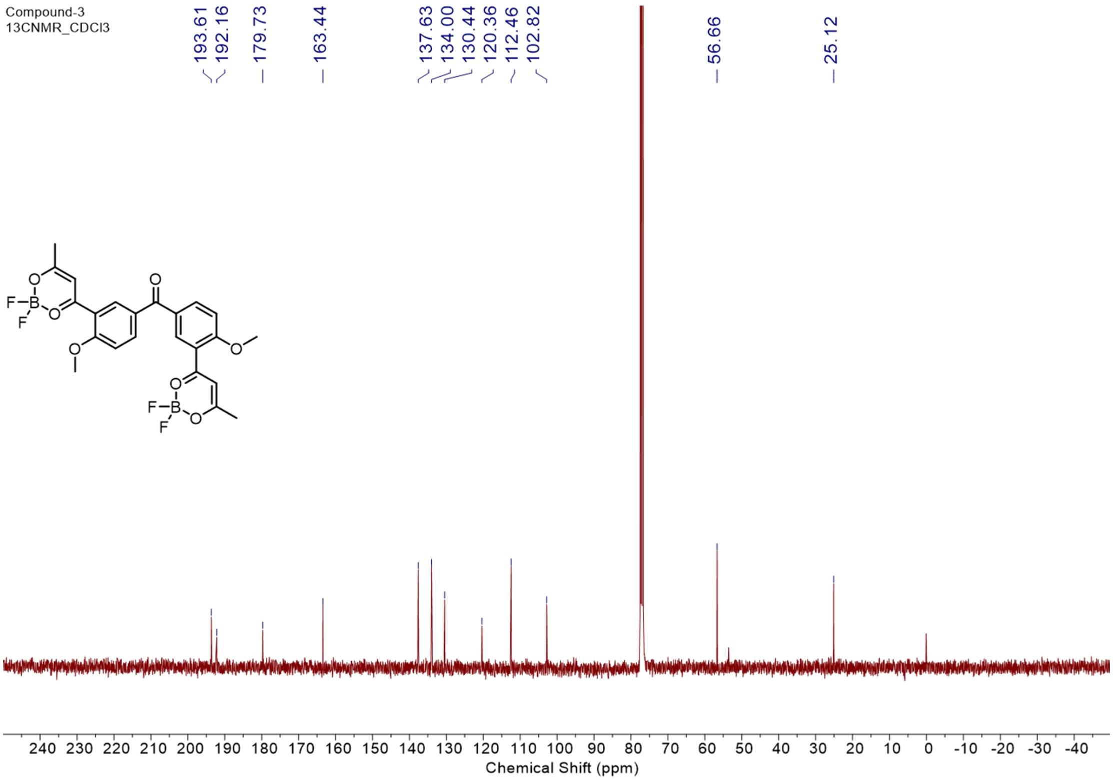

Supplementary Fig. 34 $^{13}C\{^{1}H\}$ NMR spectrum of compound **3** in $CDCl_3$.

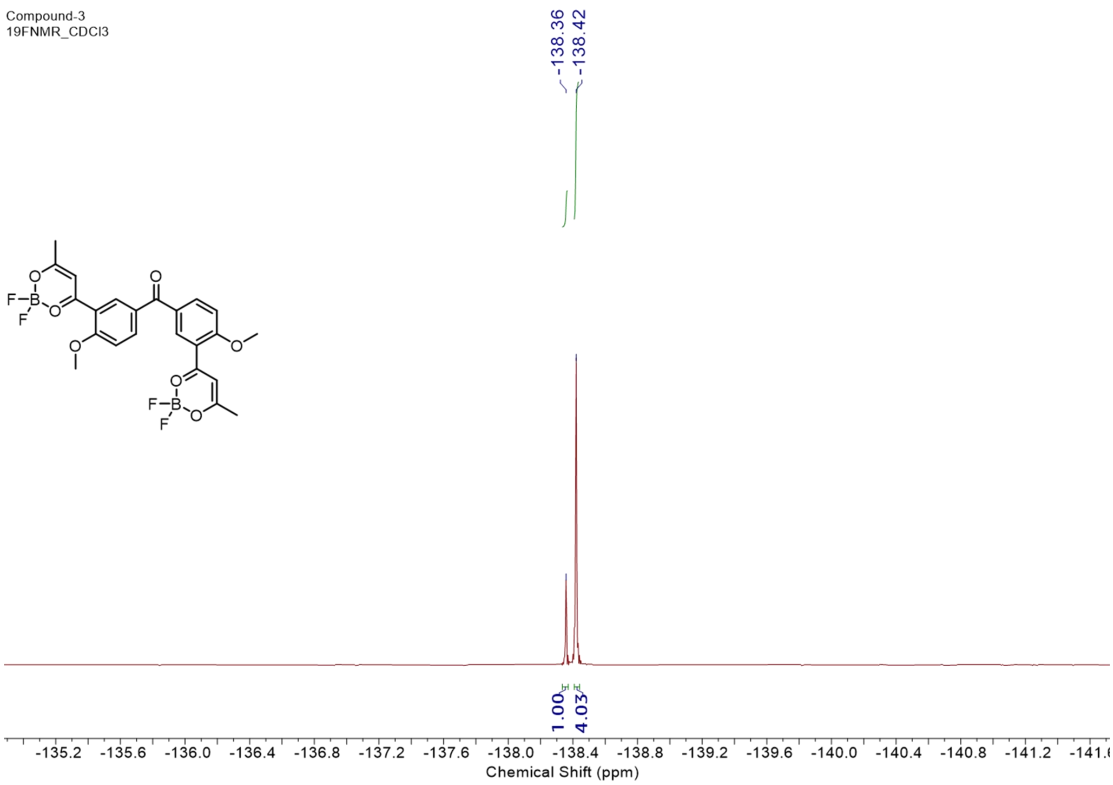

Supplementary Fig. 35 $^{19}F\{^{1}H\}$ NMR spectrum of compound **3** in $CDCl_3$.

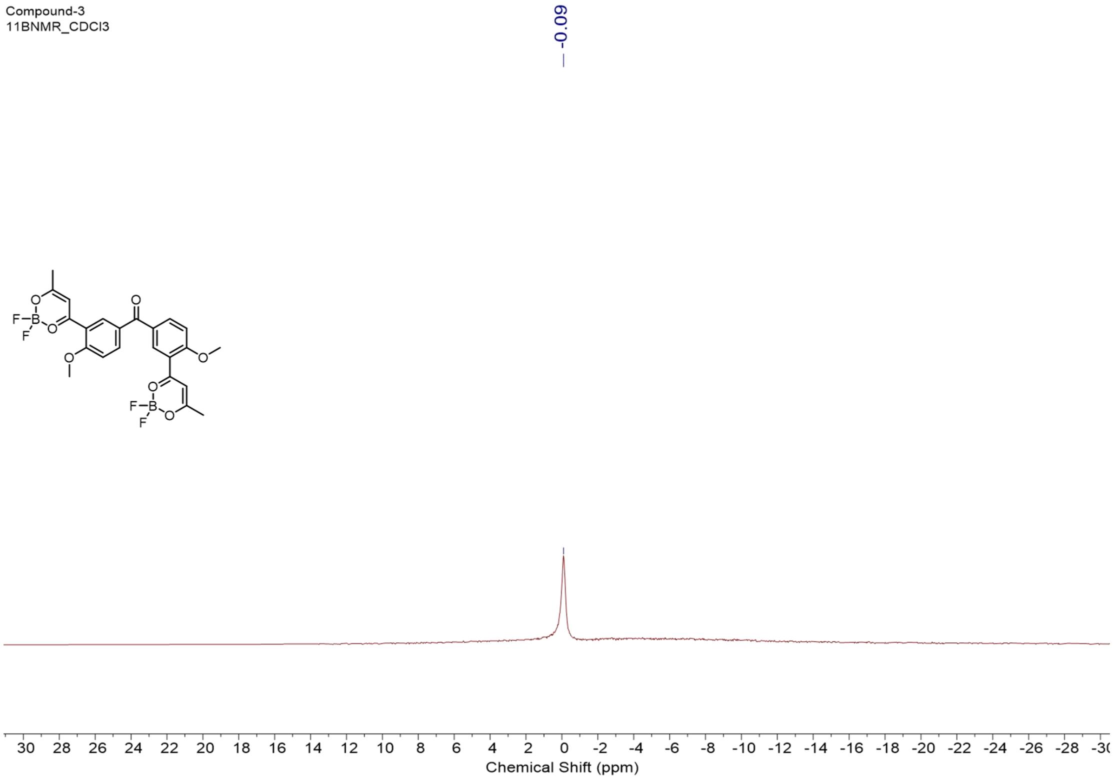

Supplementary Fig. 36 $^{11}B\{^{1}H\}$ NMR spectrum of compound **3** in $CDCl_3$.

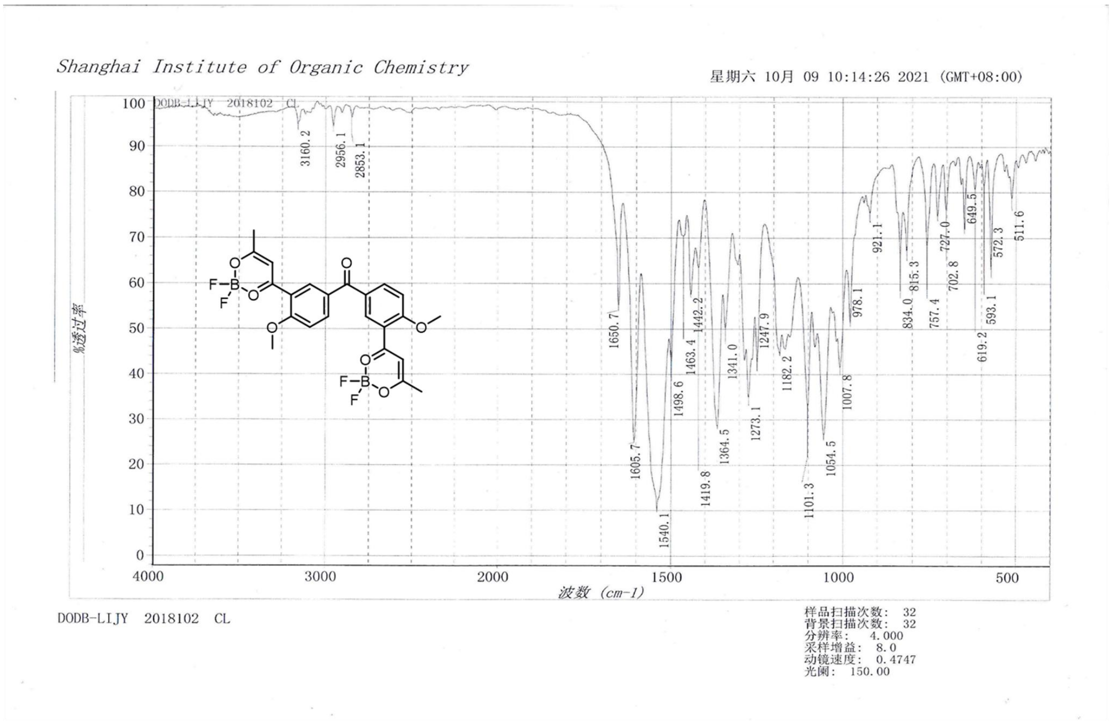

Supplementary Fig. 37 FT-IR spectrum of compound **3.**

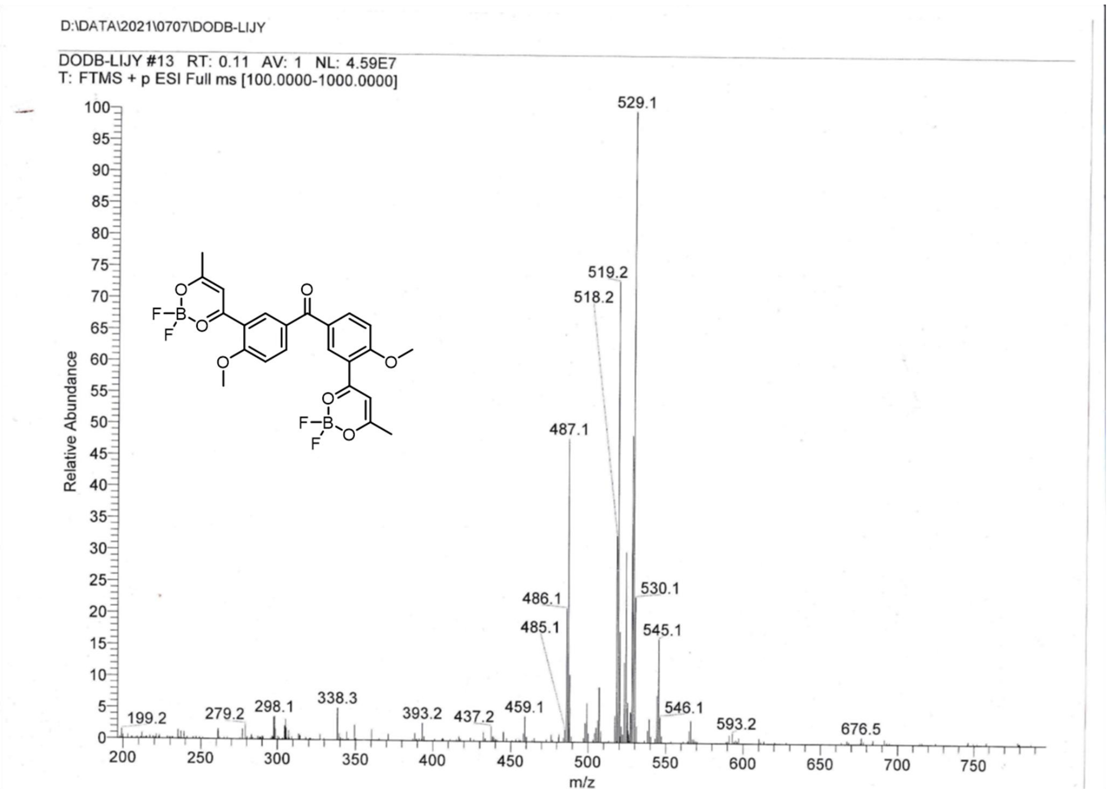

Supplementary Fig. 38 LRMS spectrum of compound **3**.

National Center for Organic Mass Spectrometry in Shanghai
Shanghai Institute of Organic Chemistry
Chinese Academic of Sciences
High Resolution ESI-MS REPORT

Instrument: Thermo Scientific Q Exactive HF Orbitrap-FTMS

Card Serial Number: E211704

Sample Serial Number: DODB-LIJY

Operator: Songw Date: 2021/07/07

Operation Mode: ESI Positive Ion Mode

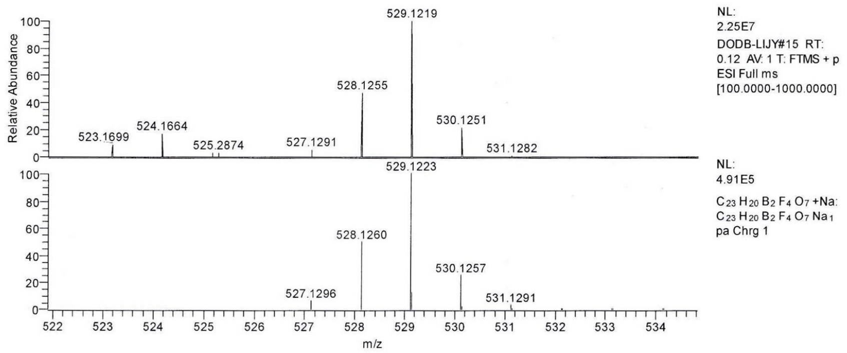


```
Elemental composition search on mass 527.1291

m/z= 522.1291-532.1291
   m/z      Theo.     Delta     RDB      Composition
            Mass      (ppm)    equiv.
 527.1291  527.1296   -0.99    12.5 C23 H20 O7 10B2 F4 Na
```

Supplementary Fig. 39 HRMS result of compound **3**.

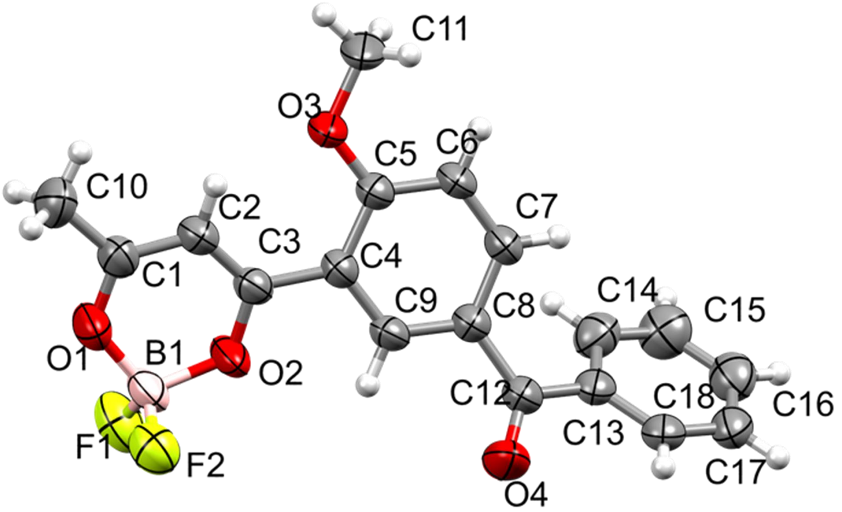

Supplementary Fig. 40 Single crystal structures of compound **1** (CCDC deposit number: 2156303).

2. Supplementary Tables

Supplementary Table 1 Photophysical properties of $BPBF_2$ compounds in dichloromethane solution.

Entry	λ_{abs} (nm)	ε (10^4 $M^{-1}cm^{-1}$)	λ_F (nm)	τ_F (ns)	Φ_F (%)
compound **1**	360	1.54	423	0.4	5.3
compound **2**	359	2.24	427	1.0	2.9
compound **3**	359	3.38	422	1.1	5.6

* Quinine sulfate dihydrate in 0.5 M H_2SO_4 as reference.

Supplementary Table 2 S_n, T_n excitation energy (n = 1, 2, 3, 4, 5) in eV of compound **1** calculated at the B3LYP/G/def2-TZVP level of theory. The smallest S_1, T_1 and T_2 energies are underlined.

	S_0 geometry	S_1 geometry	T_1 geometry	T_2 geometry
S_1	3.560	**2.368**	3.379	3.264
S_2	3.605	3.378	3.583	3.515
S_3	3.990	3.425	3.742	3.778
S_4	4.195	3.720	3.829	3.942
S_5	4.230	3.794	4.008	4.055
T_1	2.775	2.297	**2.266**	2.554
T_2	2.980	2.711	2.938	**2.683**
T_3	3.153	2.765	3.042	3.007
T_4	3.439	3.228	3.406	3.193
T_5	3.480	3.356	3.455	3.357

Supplementary Table 3 S_n, T_n excitation energy (n = 1, 2, 3, 4, 5) in eV of compound **2** calculated at the B3LYP/G/def2-TZVP level of theory. The smallest S_1, T_1 and T_2 energies are underlined.

	S_0 geometry	S_1 geometry	T_1 geometry	T_2 geometry
S_1	3.404	**1.981**	3.231	3.042
S_2	3.579	2.515	3.450	3.260
S_3	3.728	3.289	3.625	3.494
S_4	3.983	3.485	3.633	3.770
S_5	4.107	3.658	3.876	3.963
T_1	2.703	**1.963**	2.293	2.367
T_2	2.915	**2.213**	2.924	2.536
T_3	3.213	2.535	3.022	2.727
T_4	3.350	3.031	3.223	3.192
T_5	3.390	3.242	3.262	3.273

Supplementary Table 4 S_n, T_n excitation energy (n = 1, 2, 3, 4, 5) in eV of compound **3** calculated at the B3LYP/G/def2-TZVP level of theory. The smallest S_1, T_1, T_2 and T_3 energies are underlined.

	S_0 geometry	S_1 geometry	T_1 geometry	T_2 geometry	T_3 geometry
S_1	3.407	**2.192**	3.233	3.339	3.281
S_2	3.438	2.875	3.423	3.363	3.480
S_3	3.621	3.024	3.472	3.582	3.517
S_4	3.652	3.309	3.622	3.627	3.624
S_5	3.918	3.484	3.772	3.785	3.783
T_1	2.680	**2.155**	2.258	2.548	2.218
T_2	2.791	**2.482**	2.787	2.555	2.810
T_3	2.957	**2.517**	2.934	2.903	2.936
T_4	3.120	2.635	3.018	3.002	3.050
T_5	3.171	3.061	3.150	3.103	3.183

Supplementary Table 5 S_n, T_n excitation energy (n = 1, 2, 3, 4, 5) in eV of compound **1** calculated at the B3LYP/G/def2-TZVP(-f) level of theory (PCM: dichloromethane). The smallest S_1, T_1 and T_2 energies are underlined.

	S_0 geometry	S_1 geometry	T_1 geometry	T_2 geometry
S_1	3.452	**2.508**	3.202	3.305
S_2	3.778	3.427	3.661	3.322
S_3	4.030	3.702	3.698	3.794
S_4	4.162	3.793	3.771	3.831
S_5	4.176	3.869	3.888	3.958
T_1	2.829	2.457	**2.250**	2.551
T_2	3.094	2.818	2.885	**2.712**
T_3	3.242	2.834	3.092	3.010
T_4	3.448	3.291	3.372	3.135
T_5	3.581	3.388	3.473	3.201

Supplementary Table 6 S_n, T_n excitation energy (n = 1, 2, 3, 4, 5) in eV of compound **2** calculated at the B3LYP/G/def2-TZVP(-f) level of theory (PCM: dichloromethane). The smallest S_1, T_1 and T_2 energies are underlined.

	S_0 geometry	S_1 geometry	T_1 geometry	T_2 geometry
S_1	3.305	<u>**2.333**</u>	3.088	3.046
S_2	3.365	3.097	3.195	3.317
S_3	3.741	3.182	3.620	3.337
S_4	3.901	3.473	3.768	3.725
S_5	3.957	3.741	3.850	3.747
T_1	2.657	2.312	<u>**2.300**</u>	2.547
T_2	2.976	<u>**2.483**</u>	2.848	2.605
T_3	3.061	2.668	2.966	2.992
T_4	3.252	2.841	3.091	3.046
T_5	3.306	3.179	3.241	3.091

Supplementary Table 7 S_n, T_n excitation energy (n = 1, 2, 3, 4, 5) in eV of compound **3** calculated at the B3LYP/G/def2-TZVP(-f) level of theory (PCM: dichloromethane). The smallest S_1, T_1 and T_2 energies are underlined.

	S_0 geometry	S_1 geometry	T_1 geometry	T_2 geometry
S_1	3.408	<u>**2.405**</u>	3.201	3.316
S_2	3.410	2.761	3.317	3.402
S_3	3.532	2.931	3.400	3.423
S_4	3.608	3.641	3.615	3.611
S_5	3.766	3.663	3.719	3.738
T_1	2.629	<u>**2.150**</u>	2.267	2.480
T_2	2.727	<u>**2.381**</u>	2.767	2.494
T_3	3.045	2.647	2.897	3.002
T_4	3.078	2.762	3.024	3.034
T_5	3.177	2.860	3.161	3.152

Supplementary Table 8 S_n, T_n excitation energy (n = 1, 2, 3, 4, 5) in eV of compound **1** calculated at the TD-ωB97X-D3/def2-TZVP(-f) level of theory (ω = 0.1730 $Bohr^{-1}$). The smallest S_1, T_1 and T_2 energies are underlined.

	S_0 geometry	S_1 geometry	T_1 geometry	T_2 geometry
S_1	3.805	**<u>3.135</u>**	3.578	3.480
S_2	4.042	4.002	3.814	3.944
S_3	4.566	4.403	4.163	4.424
S_4	4.790	4.581	4.507	4.628
S_5	4.911	4.703	4.708	4.807
T_1	2.928	2.472	**<u>2.307</u>**	2.732
T_2	3.151	2.898	3.121	**<u>2.847</u>**
T_3	3.366	2.995	3.261	3.214
T_4	3.607	3.241	3.600	3.354
T_5	3.788	3.663	3.613	3.702

Supplementary Table 9 S_n, T_n excitation energy (n = 1, 2, 3, 4, 5) in eV of compound **2** calculated at the TD-ωB97X-D3/def2-TZVP(-f) level of theory (ω = 0.1839 $Bohr^{-1}$). The smallest S_1, T_1 and T_2 energies are underlined.

	S_0 geometry	S_1 geometry	T_1 geometry	T_2 geometry
S_1	3.887	**<u>3.180</u>**	3.856	3.111
S_2	4.232	4.203	4.029	4.344
S_3	4.644	4.470	4.356	4.391
S_4	4.877	4.571	4.571	4.556
S_5	4.973	4.709	4.700	4.726
T_1	2.977	2.612	**<u>2.448</u>**	2.494
T_2	3.300	2.966	3.273	**<u>2.969</u>**
T_3	3.570	3.192	3.546	2.989
T_4	3.655	3.460	3.609	3.485
T_5	3.943	3.770	3.725	3.955

Supplementary Table 10 S_n, T_n excitation energy (n = 1, 2, 3, 4, 5) in eV of compound **3** calculated at the TD-ωB97X-D3/def2-TZVP(-f) level of theory (ω = 0.1839 $Bohr^{-1}$). The smallest S_1, T_1, T_2 and T_3 energies are underlined.

	S_0 geometry	S_1 geometry	T_1 geometry	T_2 geometry	T_3 geometry
S_1	3.858	**<u>3.167</u>**	3.761	3.761	3.590
S_2	4.015	3.936	3.854	3.853	3.917
S_3	4.162	4.144	4.105	4.105	4.135
S_4	4.617	4.341	4.178	4.178	4.511
S_5	4.712	4.583	4.321	4.321	4.614
T_1	2.945	2.600	2.392	**<u>2.391</u>**	2.897
T_2	3.077	2.930	2.939	2.940	**<u>2.923</u>**
T_3	3.261	3.071	3.258	3.258	**<u>3.002</u>**
T_4	3.477	3.150	3.352	3.351	3.363
T_5	3.544	3.341	3.526	3.526	3.451

Supplementary Table 11 S_1, T_1 and T_n lowest excitation energy of $BPBF_2$ compounds **1-3** calculated at TD-B3LYP/G/def2-TZVP(-f) level without and with PCM solvent (n =2 for compounds **1** and **2**, n = 3 for compound **3**).

Method	Compound	S_1/eV	T_1/eV	T_n/eV
TD-B3LYP (Gas)	**1**	2.368	2.266	2.683
	**2**	1.981	1.963	2.213
	**3**	2.192	2.155	2.517
TD-B3LYP (DCM)	**1**	2.508	2.250	2.712
	**2**	2.333	2.300	2.483
	**3**	2.405	2.150	2.647
TD-ωB97X-D3	**1**	3.135	2.307	2.847
	**2**	3.180	2.448	2.969
	**3**	3.167	2.391	3.002
Exp.[a]	**1**	2.838	2.567	2.945
	**2**	2.870	2.616	2.945
	**3**	2.857	2.667	2.924

a. Experimental data is estimated from emission maxima.

Supplementary Table 12 Absolute value of SOCMEs (cm^{-1}) calculated at TD-B3LYP/G/def2-TZVP(-f) level of theory of compound **1**.

	S_0 geometry	S_1 geometry	T_1 geometry	T_2 geometry
$\|\langle T_1\|\mathcal{H}_{SOC}\|S_0\rangle\|$	6.933	3.700	1.701	17.588
$\|\langle T_2\|\mathcal{H}_{SOC}\|S_0\rangle\|$	32.032	28.577	19.620	35.031
$\|\langle T_3\|\mathcal{H}_{SOC}\|S_0\rangle\|$	22.690	1.926	32.776	17.774
$\|\langle T_4\|\mathcal{H}_{SOC}\|S_0\rangle\|$	19.926	32.122	13.392	19.896
$\|\langle T_5\|\mathcal{H}_{SOC}\|S_0\rangle\|$	10.663	11.097	19.816	3.131
$\|\langle T_1\|\mathcal{H}_{SOC}\|S_1\rangle\|$	4.501	1.085	0.829	13.432
$\|\langle T_2\|\mathcal{H}_{SOC}\|S_1\rangle\|$	3.229	1.829	1.823	10.044
$\|\langle T_3\|\mathcal{H}_{SOC}\|S_1\rangle\|$	2.347	2.869	2.457	17.313
$\|\langle T_4\|\mathcal{H}_{SOC}\|S_1\rangle\|$	7.101	0.912	1.009	23.480
$\|\langle T_5\|\mathcal{H}_{SOC}\|S_1\rangle\|$	3.826	2.432	3.417	4.169

Supplementary Table 13 Absolute value of SOCMEs (cm^{-1}) calculated at TD-B3LYP/G/def2-TZVP(-f) level of theory of compound **2**.

	S_0 geometry	S_1 geometry	T_1 geometry	T_2 geometry
$\|\langle T_1\|\mathcal{H}_{SOC}\|S_0\rangle\|$	5.287	13.139	1.019	3.251
$\|\langle T_2\|\mathcal{H}_{SOC}\|S_0\rangle\|$	29.646	37.531	25.146	44.086
$\|\langle T_3\|\mathcal{H}_{SOC}\|S_0\rangle\|$	25.292	1.739	15.323	12.366
$\|\langle T_4\|\mathcal{H}_{SOC}\|S_0\rangle\|$	20.095	2.838	3.130	10.320
$\|\langle T_5\|\mathcal{H}_{SOC}\|S_0\rangle\|$	7.302	17.763	30.558	5.550
$\|\langle T_1\|\mathcal{H}_{SOC}\|S_1\rangle\|$	1.556	0.262	1.413	32.269
$\|\langle T_2\|\mathcal{H}_{SOC}\|S_1\rangle\|$	1.850	0.460	1.558	2.569
$\|\langle T_3\|\mathcal{H}_{SOC}\|S_1\rangle\|$	2.863	3.223	2.098	1.431
$\|\langle T_4\|\mathcal{H}_{SOC}\|S_1\rangle\|$	2.623	6.711	0.519	10.871
$\|\langle T_5\|\mathcal{H}_{SOC}\|S_1\rangle\|$	1.105	5.110	0.319	9.724

Supplementary Table 14 Absolute value of SOCMEs (cm^{-1}) calculated at TD-B3LYP/G/def2-TZVP(-f) level of theory of compound **3**.

	S_0 geometry	S_1 geometry	T_1 geometry	T_2 geometry	T_3 geometry
$\|\langle T_1\|\mathcal{H}_{SOC}\|S_0\rangle\|$	5.294	11.978	1.741	4.835	1.796
$\|\langle T_2\|\mathcal{H}_{SOC}\|S_0\rangle\|$	9.120	29.124	9.954	2.399	10.437
$\|\langle T_3\|\mathcal{H}_{SOC}\|S_0\rangle\|$	24.442	11.668	21.640	20.161	21.038
$\|\langle T_4\|\mathcal{H}_{SOC}\|S_0\rangle\|$	8.088	2.476	8.774	17.755	10.115
$\|\langle T_5\|\mathcal{H}_{SOC}\|S_0\rangle\|$	9.728	1.327	16.333	6.812	16.809
$\|\langle T_1\|\mathcal{H}_{SOC}\|S_1\rangle\|$	0.310	0.336	0.851	0.420	0.962
$\|\langle T_2\|\mathcal{H}_{SOC}\|S_1\rangle\|$	0.263	0.965	0.285	0.426	0.335
$\|\langle T_3\|\mathcal{H}_{SOC}\|S_1\rangle\|$	1.043	2.615	1.037	1.013	1.137
$\|\langle T_4\|\mathcal{H}_{SOC}\|S_1\rangle\|$	0.753	0.266	1.684	0.813	1.751
$\|\langle T_5\|\mathcal{H}_{SOC}\|S_1\rangle\|$	0.633	4.144	1.431	0.476	1.646

Supplementary Table 15 Absolute value of SOCMEs (cm^{-1}) of compound **1** calculated at TD-ωB97X-D3/def2-TZVP(-f) level of theory (ω = 0.1730 $Bohr^{-1}$).

	S_0 geometry	S_1 geometry	T_1 geometry	T_2 geometry
$\|\langle T_1\|\mathcal{H}_{SOC}\|S_0\rangle\|$	4.937	36.374	0.943	16.465
$\|\langle T_2\|\mathcal{H}_{SOC}\|S_0\rangle\|$	29.718	28.449	19.917	32.605
$\|\langle T_3\|\mathcal{H}_{SOC}\|S_0\rangle\|$	26.335	11.288	33.966	22.059
$\|\langle T_4\|\mathcal{H}_{SOC}\|S_0\rangle\|$	19.974	2.365	7.920	18.938
$\|\langle T_5\|\mathcal{H}_{SOC}\|S_0\rangle\|$	5.242	3.547	21.253	3.995
$\|\langle T_1\|\mathcal{H}_{SOC}\|S_1\rangle\|$	6.323	21.917	0.405	11.938
$\|\langle T_2\|\mathcal{H}_{SOC}\|S_1\rangle\|$	12.942	26.182	1.465	13.232
$\|\langle T_3\|\mathcal{H}_{SOC}\|S_1\rangle\|$	4.674	20.592	0.609	17.128
$\|\langle T_4\|\mathcal{H}_{SOC}\|S_1\rangle\|$	13.666	6.413	1.232	17.678
$\|\langle T_5\|\mathcal{H}_{SOC}\|S_1\rangle\|$	9.310	10.027	1.087	10.562

Supplementary Table 16 Absolute value of SOCMEs (cm^{-1}) of compound **2** calculated at TD-ωB97X-D3/def2-TZVP(-f) level of theory (ω = 0.1839 $Bohr^{-1}$).

	S_0 geometry	S_1 geometry	T_1 geometry	T_2 geometry
$\|\langle T_1\|\mathcal{H}_{SOC}\|S_0\rangle\|$	2.122	46.832	1.197	41.733
$\|\langle T_2\|\mathcal{H}_{SOC}\|S_0\rangle\|$	40.712	7.670	40.932	26.092
$\|\langle T_3\|\mathcal{H}_{SOC}\|S_0\rangle\|$	8.092	21.426	5.866	17.136
$\|\langle T_4\|\mathcal{H}_{SOC}\|S_0\rangle\|$	26.209	9.135	26.838	4.171
$\|\langle T_5\|\mathcal{H}_{SOC}\|S_0\rangle\|$	5.258	2.644	4.524	2.245
$\|\langle T_1\|\mathcal{H}_{SOC}\|S_1\rangle\|$	1.691	11.441	1.156	16.548
$\|\langle T_2\|\mathcal{H}_{SOC}\|S_1\rangle\|$	10.390	6.680	10.292	23.223
$\|\langle T_3\|\mathcal{H}_{SOC}\|S_1\rangle\|$	2.313	32.292	2.888	16.662
$\|\langle T_4\|\mathcal{H}_{SOC}\|S_1\rangle\|$	14.930	6.942	15.214	15.722
$\|\langle T_5\|\mathcal{H}_{SOC}\|S_1\rangle\|$	7.982	10.185	0.300	4.590

Supplementary Table 17 Absolute value of SOCMEs (cm^{-1}) of compound **3** calculated at TD-ωB97X-D3/def2-TZVP(-f) level of theory (ω = 0.1657 $Bohr^{-1}$).

	S_0 geometry	S_1 geometry	T_1 geometry	T_2 geometry	T_3 geometry
$\|\langle T_1\|\mathcal{H}_{SOC}\|S_0\rangle\|$	4.165	44.262	0.523	0.532	13.604
$\|\langle T_2\|\mathcal{H}_{SOC}\|S_0\rangle\|$	4.912	8.794	4.360	3.695	8.391
$\|\langle T_3\|\mathcal{H}_{SOC}\|S_0\rangle\|$	35.542	11.514	31.245	31.449	36.048
$\|\langle T_4\|\mathcal{H}_{SOC}\|S_0\rangle\|$	6.941	20.674	25.879	25.685	23.537
$\|\langle T_5\|\mathcal{H}_{SOC}\|S_0\rangle\|$	26.176	7.811	21.641	21.771	16.787
$\|\langle T_1\|\mathcal{H}_{SOC}\|S_1\rangle\|$	2.613	12.851	2.471	2.470	6.615
$\|\langle T_2\|\mathcal{H}_{SOC}\|S_1\rangle\|$	2.816	8.753	0.262	0.262	3.464
$\|\langle T_3\|\mathcal{H}_{SOC}\|S_1\rangle\|$	11.028	9.591	4.864	4.838	10.935
$\|\langle T_4\|\mathcal{H}_{SOC}\|S_1\rangle\|$	2.519	29.054	5.848	5.876	11.544
$\|\langle T_5\|\mathcal{H}_{SOC}\|S_1\rangle\|$	7.447	4.677	0.768	0.741	13.130

Supplementary Table 18 Transition electric dipole moments (a.u) of compound **1** calculated at TD-ωB97X-D3/def2-TZVP(-f) level of theory (ω = 0.1730 $Bohr^{-1}$).

	S_0 geometry	S_1 geometry	T_1 geometry	T_2 geometry
$T_1 - S_0$	$7.2422*10^{-4}$	$4.3189*10^{-3}$	$1.8439*10^{-4}$	$1.4511*10^{-3}$
$T_2 - S_0$	$2.0340*10^{-3}$	$7.0351*10^{-3}$	$8.8284*10^{-4}$	$2.8084*10^{-3}$
$S_1 - S_0$	$4.2192*10^{-3}$	$1.9372*10^{-1}$	2.2471	$9.2391*10^{-2}$

Supplementary Table 19 Transition electric dipole moments (a.u) of compound **2** calculated at TD-ωB97X-D3/def2-TZVP(-f) level of theory (ω = 0.1839 $Bohr^{-1}$).

	S_0 geometry	S_1 geometry	T_1 geometry	T_2 geometry
$T_1 - S_0$	$5.3217*10^{-4}$	$5.7785*10^{-3}$	$3.1097*10^{-4}$	$4.9862*10^{-3}$
$T_2 - S_0$	$3.3899*10^{-3}$	$1.4954*10^{-3}$	$3.1980*10^{-3}$	$7.6159*10^{-3}$
$S_1 - S_0$	$1.7795*10^{-1}$	$3.0946*10^{-1}$	$2.0112*10^{-1}$	$2.3903*10^{-1}$

Supplementary Table 20 Transition electric dipole moments (a.u) of compound **3** calculated at TD-ωB97X-D3/def2-TZVP(-f) level of theory (ω = 0.1657 $Bohr^{-1}$).

	S_0 geometry	S_1 geometry	T_1 geometry	T_2 geometry	T_3 geometry
$T_1 - S_0$	$2.9155*10^{-4}$	$5.4428*10^{-3}$	$4.1304*10^{-4}$	$4.1461*10^{-4}$	$8.8916*10^{-4}$
$T_2 - S_0$	$5.0626*10^{-4}$	$1.9096*10^{-3}$	$3.0067*10^{-4}$	$3.2047*10^{-4}$	$8.4380*10^{-4}$
$T_3 - S_0$	$2.5165*10^{-3}$	$4.6311*10^{-3}$	$2.5718*10^{-3}$	$2.5445*10^{-3}$	$3.1489*10^{-3}$
$S_1 - S_0$	$1.5087*10^{-1}$	$3.5175*10^{-1}$	2.4355	2.4425	$1.9719*10^{-1}$

Supplementary Table 21 The ground state (S_0) and excited state (S_n and T_n, n = 1, 2, 3, 4, 5) electronic energies in eV of compound **1** calculated at the TD-ωB97X-D3/def2-TZVP(-f) level of theory (ω = 0.1730 $Bohr^{-1}$). The zero point of the energies is set to be the S_0 energy for the S_0 geometry. The smallest S_1, T_1 and T_2 energies are underlined.

	S_0 geometry	S_1 geometry	T_1 geometry	T_2 geometry
S_0	0.000	0.387	0.357	0.175
S_1	3.805	**3.522**	3.935	3.655
S_2	4.042	4.389	4.171	4.119
S_3	4.566	4.790	4.520	4.599
S_4	4.790	4.968	4.864	4.803
S_5	4.911	5.090	5.065	4.982
T_1	2.928	2.859	**2.664**	2.907
T_2	3.151	3.285	3.478	**3.022**
T_3	3.366	3.382	3.618	3.389
T_4	3.607	3.628	3.957	3.529
T_5	3.788	4.050	3.970	3.877

Supplementary Table 22 The ground state (S_0) and excited state (S_n and T_n, n = 1, 2, 3, 4, 5) electronic energyies in eV of compound **2** calculated at the TD-ωB97X-D3/def2-TZVP(-f) level of theory (ω = 0.1839 $Bohr^{-1}$). The zero point of the energies is set to be the S_0 energy for the S_0 geometry. The smallest S_1, T_1 and T_2 energies are underlined.

	S_0 geometry	S_1 geometry	T_1 geometry	T_2 geometry
S_0	0.000	0.416	0.459	0.626
S_1	3.887	**<u>3.596</u>**	4.315	3.737
S_2	4.232	4.619	4.488	4.970
S_3	4.644	4.886	4.815	5.017
S_4	4.877	4.987	5.030	5.182
S_5	4.973	5.125	5.159	5.352
T_1	2.977	3.028	**<u>2.907</u>**	3.120
T_2	**<u>3.300</u>**	3.382	3.732	3.595
T_3	3.570	3.608	4.005	3.615
T_4	3.655	3.876	4.068	4.111
T_5	3.943	4.186	4.184	4.581

Supplementary Table 23 The ground state (S_0) and excited state (S_n and T_n, n = 1, 2, 3, 4, 5) electronic energyies in eV of compound **3** calculated at the TD-ωB97X-D3/def2-TZVP(-f) level of theory (ω = 0.1657 $Bohr^{-1}$). The zero point of the energies is set to be the S_0 energy for the S_0 geometry. The smallest S_1, T_1, T_2, and T_3 energies are underlined.

	S_0 geometry	S_1 geometry	T_1 geometry	T_2 geometry	T_3 geometry
S_0	0.000	0.395	0.463	0.459	0.127
S_1	3.858	**<u>3.562</u>**	4.224	4.220	3.717
S_2	4.015	4.331	4.317	4.312	4.044
S_3	4.162	4.539	4.568	4.564	4.262
S_4	4.617	4.736	4.641	4.637	4.638
S_5	4.712	4.978	4.784	4.780	4.741
T_1	2.945	2.995	2.855	**<u>2.850</u>**	3.024
T_2	3.077	3.325	3.402	3.399	**<u>3.050</u>**
T_3	3.261	3.466	3.721	3.717	**<u>3.129</u>**
T_4	3.477	3.545	3.815	3.810	3.490
T_5	3.544	3.736	3.989	3.985	3.578

Supplementary Table 24 Nonradiative and radiative transition rate constants (s^{-1}) calculated for the S_0, S_1, T_1 and T_2 geometries of compound **1**. The rate constants were calculated based on frequency analysis at the ωB97XD/6-31G(d,p) level of theory and TD-DFT calculated results at the ωB97X-D3/def2-TZVP(-f) level of theory (ω=0.1730 $Bohr^{-1}$). The temperature was set to 300 K and γ was set to 1000 cm^{-1}.

	**S_0 geometry**	**S_1 geometry**	**T_1 geometry**	**T_2 geometry**
		**Intersystem Crossing (ISC)**		
**S_1->T_1**	$3.08*10^{8}$	$3.70*10^{9}$	$1.26*10^{6}$	$1.10*10^{9}$
**S_1->T_2**	$3.66*10^{9}$	$1.50*10^{10}$	$4.68*10^{7}$	$3.82*10^{9}$
**T_1->S_1**	$5.70*10^{-7}$	$2.69*10^{-2}$	$5.62*10^{-16}$	$2.98*10^{-4}$
**T_2->S_1**	$6.76*10^{-6}$	$1.56*10^{6}$	$9.85*10^{-1}$	$8.88*10^{-2}$
		**Internal Conversion (IC)**		
**T_1->T_2**	$2.57*10^{8}$	$1.00*10^{5}$	$2.28*10^{-3}$	$6.31*10^{10}$
**T_2->T_1**	$1.43*10^{12}$	$3.93*10^{11}$	$1.08*10^{11}$	$5.39*10^{12}$
		**Radiative Decay**		
**S_1->S_0**	$1.04*10^{4}$	$2.19*10^{7}$	$2.95*10^{9}$	$4.98*10^{6}$
**T_1->S_0**	$4.42*10^{1}$	$1.57*10^{3}$	2.86	$1.77*10^{2}$
**T_2->S_0**	$5.08*10^{2}$	$6.08*10^{3}$	$9.58*10^{1}$	$9.69*10^{2}$

Supplementary Table 25 Nonradiative and radiative transition rate constants (s^{-1}) calculated for the S_0, S_1, T_1 and T_2 geometries of compound **2**. The rate constants were calculated based on frequency analysis at the ωB97XD/6-31G(d,p) level of theory and TD-DFT calculated results at the ωB97X-D3/def2-TZVP(-f) level of theory (ω=0.1839 $Bohr^{-1}$). The temperature was set to 300 K and γ was set to 1000 cm^{-1}.

	**S_0 geometry**	**S_1 geometry**	**T_1 geometry**	**T_2 geometry**
		**Intersystem Crossing (ISC)**		
**S_1->T_1**	$3.38*10^{7}$	$1.55*10^{9}$	$1.58*10^{7}$	$3.24*10^{9}$
**S_1->T_2**	$6.07*10^{9}$	$2.51*10^{9}$	$5.96*10^{9}$	$3.03*10^{10}$
**T_1->S_1**	$1.74*10^{-8}$	$4.44*10^{-1}$	$3.51*10^{-17}$	$1.40*10^{-1}$
**T_2->S_1**	$8.36*10^{-1}$	$6.38*10^{5}$	$9.57*10^{-1}$	$1.25*10^{8}$
		**Internal Conversion (IC)**		
**T_1->T_2**	$8.25*10^{4}$	$2.49*10^{4}$	$4.66*10^{-5}$	$1.07*10^{2}$
**T_2->T_1**	$2.20*10^{10}$	$1.83*10^{10}$	$3.37*10^{9}$	$1.02*10^{10}$
		**Radiative Decay**		
**S_1->S_0**	$1.97*10^{7}$	$5.95*10^{7}$	$2.51*10^{7}$	$3.55*10^{7}$
**T_1->S_0**	$3.10*10^{1}$	$3.65*10^{3}$	$1.06*10^{1}$	$2.72*10^{3}$
**T_2->S_0**	$1.84*10^{3}$	$3.58*10^{2}$	$1.64*10^{3}$	$9.28*10^{3}$

Supplementary Table 26 Nonradiative and radiative transition rate constants (s^{-1}) calculated for the S_0, S_1, T_1 and T_2 geometries of compound **3**. The rate constants were calculated based on frequency analysis at the ωB97XD/6-31G(d,p) level of theory and TD-DFT calculated results at the ωB97X-D3/def2-TZVP(-f) level of theory (ω=0.1657 $Bohr^{-1}$). The temperature was set to 300 K and γ was set to 1000 cm^{-1}.

Transition	S_0 geometry	S_1 geometry	T_1 geometry	T_2 geometry	T_3 geometry
			**Intersystem Crossing (ISC)**		
**S_1->T_1**	$7.57*10^{7}$	$1.83*10^{9}$	$6.77*10^{7}$	$6.76*10^{7}$	$4.85*10^{8}$
**S_1->T_2**	$1.65*10^{8}$	$1.60*10^{9}$	$1.43*10^{6}$	$1.43*10^{6}$	$2.50*10^{8}$
**S_1->T_3**	$3.47*10^{9}$	$2.63*10^{9}$	$6.75*10^{8}$	$6.68*10^{8}$	$3.41*10^{9}$
**T_1->S_1**	$3.48*10^{-8}$	$5.46*10^{-1}$	$6.80*10^{-16}$	$6.53*10^{-16}$	$1.11*10^{-3}$
**T_2->S_1**	$1.25*10^{-5}$	$1.67*10^{5}$	$2.22*10^{-8}$	$2.31*10^{-8}$	$1.56*10^{-3}$
**T_3->S_1**	$3.25*10^{-1}$	$6.41*10^{7}$	2.40	2.37	$4.52*10^{-1}$
			**Internal Conversion (IC)**		
**T_1->T_2**	$8.86*10^{8}$	$6.69*10^{4}$	5.51	5.06	$1.38*10^{12}$
**T_2->T_1**	$1.46*10^{11}$	$2.34*10^{10}$	$8.52*10^{9}$	$8.45*10^{9}$	$3.77*10^{12}$
**T_2->T_3**	$1.40*10^{10}$	$1.25*10^{11}$	$2.50*10^{7}$	$2.62*10^{7}$	$4.39*10^{12}$
**T_3->T_2**	$1.72*10^{13}$	$2.93*10^{13}$	$5.72*10^{12}$	$5.76*10^{12}$	$9.33*10^{13}$
**T_1->T_3**	$1.28*10^{7}$	$1.44*10^{4}$	$9.85*10^{-4}$	$9.45*10^{-4}$	$4.08*10^{11}$
**T_3->T_1**	$2.61*10^{12}$	$1.18*10^{12}$	$3.48*10^{11}$	$3.47*10^{11}$	$2.37*10^{13}$
			**Radiative Decay**		
**S_1->S_0**	$1.37*10^{7}$	$7.47*10^{7}$	$3.58*10^{9}$	$3.60*10^{9}$	$2.35*10^{7}$
**T_1->S_0**	8.76	$3.05*10^{3}$	$1.76*10^{1}$	$1.77*10^{1}$	$8.15*10^{1}$
**T_2->S_0**	$3.24*10^{1}$	$4.61*10^{2}$	$1.14*10^{1}$	$1.30*10^{1}$	$9.00*10^{1}$
**T_3->S_0**	$8.64*10^{3}$	$2.93*10^{3}$	$9.02*10^{3}$	$8.83*10^{3}$	$1.35*10^{3}$

Supplementary Table 27 Intersystem crossing rate (s^{-1}) of compound **1** calculated from semiclassical Marcus formula at ωB97X-D3/def2-TZVP(-f) level of theory (ω=0.1730 $Bohr^{-1}$). The temperature was set to 300 K.

	S_0 geometry	S_1 geom	T_1 geom	T_2 geom
**S_1->T_1**	$1.55*10^{8}$	$1.86*10^{9}$	$6.37*10^{5}$	$5.53*10^{8}$
**S_1->T_2**	$6.60*10^{6}$	$2.70*10^{7}$	$8.46*10^{4}$	$6.90*10^{6}$
**T_1->S_1**	$3.07*10^{-14}$	$3.68*10^{-13}$	$1.26*10^{-16}$	$1.09*10^{-13}$
**T_2->S_1**	$4.25*10^{1}$	$1.74*10^{2}$	$5.44*10^{-1}$	$4.44*10^{1}$

Supplementary Table 28 Intersystem crossing rate (s^{-1}) of compound **2** calculated from semiclassical Marcus formula at ωB97X-D3/def2-TZVP(-f) level of theory (ω=0.1839 $Bohr^{-1}$). The temperature was set to 300 K.

	S_0 geometry	S_1 geometry	T_1 geometry	T_2 geometry
**S_1->T_1**	$8.58*10^{8}$	$3.93*10^{10}$	$4.01*10^{8}$	$8.21*10^{10}$
**S_1->T_2**	$1.42*10^{10}$	$5.89*10^{9}$	$1.40*10^{10}$	$7.12*10^{10}$
**T_1->S_1**	$3.57*10^{-14}$	$1.63*10^{-12}$	$1.67*10^{-14}$	$3.42*10^{-12}$
**T_2->S_1**	$4.67*10^{3}$	$1.93*10^{3}$	$4.58*10^{3}$	$2.33*10^{4}$

Supplementary Table 29 Intersystem crossing rate (s^{-1}) of compound **3** calculated from semiclassical Marcus formula at ωB97X-D3/def2-TZVP(-f) level of theory (ω=0.1657 $Bohr^{-1}$). The temperature was set to 300 K.

	S_0 geometry	S_1 geometry	T_1 geometry	T_2 geometry	T_3 geometry
**S_1->T_1**	$2.08*10^{9}$	$5.04*10^{10}$	$1.86*10^{9}$	$1.86*10^{9}$	$1.33*10^{10}$
**S_1->T_2**	$1.84*10^{9}$	$1.78*10^{10}$	$1.59*10^{7}$	$1.59*10^{7}$	$2.78*10^{9}$
**S_1->T_3**	$6.40*10^{8}$	$4.84*10^{8}$	$1.25*10^{8}$	$1.23*10^{8}$	$6.30*10^{8}$
**T_1->S_1**	$7.87*10^{-13}$	$1.90*10^{-11}$	$7.03*10^{-13}$	$7.03*10^{-13}$	$5.04*10^{-12}$
**T_2->S_1**	$2.43*10^{-10}$	$2.35*10^{-9}$	$2.11*10^{-12}$	$2.11*10^{-12}$	$3.68*10^{-10}$
**T_3->S_1**	$2.20*10^{3}$	$1.67*10^{3}$	$4.28*10^{2}$	$4.24*10^{2}$	$2.17*10^{3}$

Supplementary Table 30 Calculated internal conversion rate (s^{-1}) of compound **1-3** by FCClasses3 software. The rate constants were calculated based on frequency analysis at the ωB97XD/6-31G(d,p) level of theory and TD-DFT calculated results at the ωB97X-D3/def2-TZVP(-f) level of theory. The temperature was set to 300 K.

	$k_{IC}(T_2 \rightarrow T_1)$	$k_{IC}(T_1 \rightarrow T_2)$	$k_{IC}(T_3 \rightarrow T_1)$	$k_{IC}(T_1 \rightarrow T_3)$	$k_{IC}(T_3 \rightarrow T_2)$	$k_{IC}(T_2 \rightarrow T_3)$
**1**	$3.02*10^{10}$	$2.02*10^{8}$	\	\	\	\
**2**	$2.67*10^{11}$	$7.30*10^{8}$	\	\	\	\
**3**	$2.82*10^{13}$	$5.08*10^{9}$	$6.50*10^{10}$	$1.20*10^{8}$	$3.58*10^{9}$	$1.94*10^{8}$

Supplementary Table 31 Crystal data and structure refinement for mo_dm21102_0m.

Identification code	mo_dm21102_0m	
Empirical formula	C18 H15 B F2 O4	
Formula weight	344.11	
Temperature	296.15 K	
Wavelength	0.71073 Å	
Crystal system	orthorhombic	
Space group	Pbca	
Unit cell dimensions	a = 10.3880(18) Å	α = 90°.
	b = 16.326(3) Å	β = 90°.
	c = 19.538(3) Å	γ = 90°.
Volume	3313.5(10) $Å^3$	
Z	8	
Density (calculated)	1.380 Mg/m^3	
Absorption coefficient	0.110 mm^{-1}	
F(000)	1424.0	
Crystal size	0.18 × 0.15 × 0.02 mm^3	
Theta range for data collection	4.17 to 54.642°.	
Index ranges	-12<=h<=13, -21<=k<=19, -25<=l<=25	
Reflections collected	25067	
Independent reflections	3721 [R(int) = 0.0614]	
Completeness to theta = 27.321°	99.6 %	
Absorption correction	Semi-empirical from equivalents	
Max. and min. transmission	0.7456 and 0.6488	
Refinement method	Full-matrix least-squares on F^2	
Data / restraints / parameters	3721 / 0 / 228	
Goodness-of-fit on F^2	0.978	
Final R indices [I>2sigma(I)]	R1 = 0.0517, wR2 = 0.1343	
R indices (all data)	R1 = 0.1169, wR2 = 0.1799	
Largest diff. peak and hole	0.15 and -0.20 e.$Å^{-3}$	

Supplementary Table 32 Atomic coordinates (x10^4) and equivalent isotropic displacement parameters (Å^{2}x 10^3) for mo_dm21102_0m. U(eq) is defined as one third of the trace of the orthogonalized U^{ij} tensor.

Atom	x	y	z	U(eq)
F1	968.3(16)	3069.9(10)	4284.3(9)	86.0(6)
F2	984.7(16)	4437.0(10)	4337.2(8)	78.0(5)
O1	2190.8(18)	3797.0(13)	3502.3(10)	81.8(6)
O2	2767.3(16)	3723.9(12)	4712.3(9)	74.5(6)
O3	6708.7(15)	3935.2(12)	4474.7(8)	65.5(5)
O4	3376.3(18)	4400.3(14)	7117.6(10)	85.7(7)
C1	3385(2)	3961.0(15)	3377.1(13)	57.1(6)
C2	4278(2)	4030.9(15)	3888.8(12)	54.2(6)
C3	3950(2)	3893.4(13)	4558.5(12)	47.3(5)
C4	4826(2)	3912.5(13)	5151.1(12)	45.3(5)
C5	6181(2)	3917.4(14)	5103.9(12)	47.3(5)
C6	6923(2)	3901.2(14)	5693.9(12)	52.1(6)
C7	6352(2)	3895.7(14)	6326.1(12)	50.9(6)
C8	5015(2)	3920.6(14)	6392.8(12)	47.8(6)
C9	4279(2)	3928.5(13)	5802.6(11)	47.8(6)
C10	3704(3)	4060.4(19)	2643.2(13)	76.8(8)
C11	8085(3)	3975(3)	4415.5(16)	104.1(13)
C12	4379(2)	4012.3(16)	7066.5(12)	56.0(6)
C13	4989(2)	3649.9(15)	7682.9(13)	52.8(6)
C14	5650(3)	2910.6(16)	7657.6(14)	65.0(7)
C15	6163(3)	2578.9(18)	8247.9(16)	76.8(8)
C16	6059(3)	2990.1(18)	8859.6(15)	71.6(8)
C17	5419(2)	3726.2(18)	8888.7(14)	65.2(7)
C18	4878(2)	4053.6(17)	8304.5(13)	56.9(7)
B1	1693(3)	3749(2)	4211.7(17)	63.0(8)

Supplementary Table 33 Bond lengths [Å] and angles [°] for mo_dm21102_0m.

F(1)-B(1)	1.347(3)
F(2)-B(1)	1.366(4)
O(1)-C(1)	1.292(3)
O(1)-B(1)	1.482(4)
O(2)-C(3)	1.294(3)
O(2)-B(1)	1.485(3)
O(3)-C(5)	1.346(3)
O(3)-C(11)	1.435(3)
O(4)-C(12)	1.224(3)
C(1)-C(2)	1.369(3)
C(1)-C(10)	1.481(4)
C(2)-C(3)	1.371(3)
C(3)-C(4)	1.473(3)
C(4)-C(5)	1.411(3)
C(4)-C(9)	1.394(3)
C(5)-C(6)	1.387(3)
C(6)-C(7)	1.370(3)
C(7)-C(8)	1.395(3)
C(8)-C(9)	1.384(3)
C(8)-C(12)	1.480(3)
C(12)-C(13)	1.484(3)
C(13)-C(14)	1.389(3)
C(13)-C(18)	1.387(4)
C(14)-C(15)	1.381(4)
C(15)-C(16)	1.375(4)
C(16)-C(17)	1.374(4)
C(17)-C(18)	1.380(4)
C(1)-O(1)-B(1)	121.6(2)
C(3)-O(2)-B(1)	123.7(2)
C(5)-O(3)-C(11)	118.7(2)
O(1)-C(1)-C(2)	122.0(2)
O(1)-C(1)-C(10)	114.9(2)
C(2)-C(1)-C(10)	123.1(2)

C(1)-C(2)-C(3)	121.0(2)
O(2)-C(3)-C(2)	119.5(2)
O(2)-C(3)-C(4)	114.1(2)
C(2)-C(3)-C(4)	126.4(2)
C(5)-C(4)-C(3)	124.4(2)
C(9)-C(4)-C(3)	117.8(2)
C(9)-C(4)-C(5)	117.8(2)
O(3)-C(5)-C(4)	117.8(2)
O(3)-C(5)-C(6)	122.2(2)
C(6)-C(5)-C(4)	120.0(2)
C(7)-C(6)-C(5)	120.6(2)
C(6)-C(7)-C(8)	121.0(2)
C(7)-C(8)-C(12)	122.0(2)
C(9)-C(8)-C(7)	118.2(2)
C(9)-C(8)-C(12)	119.6(2)
C(8)-C(9)-C(4)	122.4(2)
O(4)-C(12)-C(8)	120.3(2)
O(4)-C(12)-C(13)	120.2(2)
C(8)-C(12)-C(13)	119.4(2)
C(14)-C(13)-C(12)	121.9(2)
C(18)-C(13)-C(12)	119.1(2)
C(18)-C(13)-C(14)	119.0(3)
C(15)-C(14)-C(13)	120.1(3)
C(16)-C(15)-C(14)	120.3(3)
C(17)-C(16)-C(15)	120.1(3)
C(16)-C(17)-C(18)	120.1(3)
C(17)-C(18)-C(13)	120.4(3)
F(1)-B(1)-F(2)	110.9(2)
F(1)-B(1)-O(1)	109.7(2)
F(1)-B(1)-O(2)	109.2(2)
F(2)-B(1)-O(1)	108.2(2)
F(2)-B(1)-O(2)	108.0(2)
O(1)-B(1)-O(2)	110.8(2)

Supplementary Table 34 Optimized S_0 geometry of compound **1** at the B3LYP/G/6-31G(d, p) level of theory in Cartesian coordinates (Å)

Atom	X	Y	Z
C	0.06366473	3.21821077	-0.29628684
C	-1.26939023	2.90661317	-0.07535780
C	-1.68663835	1.57936522	0.10864728
C	-0.71177112	0.57650606	0.10867146
C	0.64766812	0.85876159	-0.07647155
C	1.04005226	2.20711258	-0.30409740
C	-3.13334549	1.32909951	0.39708439
C	-3.74260332	-0.01597233	0.12977156
C	-4.82093897	-0.41692489	0.93398612
C	-5.45767538	-1.63275684	0.70504647
C	-5.04409598	-2.45165729	-0.34974437
C	-3.99232171	-2.04869376	-1.17356882
C	-3.33864011	-0.83995198	-0.93206547
O	-3.82643828	2.23068291	0.85710468
C	1.62777116	-0.24619092	-0.00529686
C	1.31353072	-1.53772495	-0.45913696
C	2.22595746	-2.56771459	-0.24575257
O	3.38315297	-2.36459017	0.29248334
O	2.77190695	0.01320599	0.53031008
C	1.93633894	-3.99334196	-0.60648549
B	3.92969019	-0.97305221	0.56687319
F	4.45937916	-0.95763571	1.81906602
F	4.79994089	-0.65008501	-0.43567048
O	2.34125377	2.43455108	-0.55268842
C	2.79362350	3.76637875	-0.78066258
H	0.34901217	4.24945236	-0.46087004
H	-2.01872501	3.69040186	-0.04136427
H	-1.00163979	-0.44641683	0.31758715
H	-5.14350635	0.24291646	1.73229548
H	-6.28119668	-1.94115538	1.34210265
H	-5.54647952	-3.39688396	-0.53339799
H	-3.68464219	-2.67181275	-2.00812702
H	-2.53740571	-0.52043227	-1.59058898
H	0.36839789	-1.74172285	-0.94005370
H	0.93814333	-4.11473660	-1.02919937
H	2.68316164	-4.34477163	-1.32578730
H	2.03283286	-4.61874040	0.28675088
H	3.86811618	3.68295665	-0.94054936
H	2.32293072	4.20244718	-1.66950878
H	2.60254867	4.40573832	0.08842379

Supplementary Table 35 Optimized S_1 geometry of compound **1** at the TD-B3LYP/G/6-31G(d, p) level of theory in Cartesian coordinates (Å).

Atom	X	Y	Z
C	0.04210893	2.81953096	0.78472111
C	1.35977712	2.60547585	0.46233226
C	1.79261362	1.32584304	0.02121631
C	0.84879275	0.28036246	-0.17685458
C	-0.47373854	0.43408793	0.16210275
C	-0.87875565	1.74031611	0.64960915
C	3.23898584	1.19207163	-0.36377286
C	3.99399625	-0.06698942	-0.16434902
C	5.17472654	-0.25304204	-0.90380630
C	5.93399301	-1.40361051	-0.72673881
C	5.53281617	-2.36964529	0.20171208
C	4.37188731	-2.18329953	0.95403590
C	3.59995802	-1.03771321	0.77072949
O	3.73976336	2.18365643	-0.88916078
C	-1.55202136	-0.56891459	-0.10036203
C	-1.97878623	-1.52600990	0.80002357
C	-3.19700960	-2.17744340	0.57461570
O	-3.99740077	-1.78460617	-0.41811283
O	-2.26901234	-0.22378942	-1.18986989
C	-3.68251338	-3.35805692	1.35152865
B	-3.74313062	-0.49022717	-1.11163700
F	-4.24220188	-0.55482462	-2.38863083
F	-4.34054690	0.55527194	-0.37514344
O	-2.15269268	1.82200737	0.91264841
C	-2.90435831	3.04129597	1.03932083
H	-0.28840281	3.77523938	1.17425139
H	2.09599662	3.39538596	0.55255424
H	1.17143101	-0.66174071	-0.60363378
H	5.47219844	0.51222246	-1.61237118
H	6.83846123	-1.55061006	-1.30833325
H	6.12802817	-3.26708251	0.34027788
H	4.06773505	-2.92824034	1.68202412
H	2.70681605	-0.89280162	1.36935903
H	-1.37238316	-1.79311083	1.65808629
H	-2.99984733	-3.60895804	2.16793342
H	-4.67782083	-3.16491466	1.77146608
H	-3.78396434	-4.24040649	0.70492699
H	-3.90166578	2.75078221	0.71322736
H	-2.90435158	3.35715573	2.08611509
H	-2.49193398	3.82048907	0.39584511

Supplementary Table 36 Optimized T_1 geometry of compound **1** at the TD-B3LYP/G/6-31G(d, p) level of theory in Cartesian coordinates (Å).

Atom	X	Y	Z
C	0.08172021	3.24461637	-0.13263583
C	-1.26855942	2.94158309	0.04260336
C	-1.67954802	1.58844499	0.15507159
C	-0.72979758	0.58211936	0.13408457
C	0.67728929	0.83643792	-0.01525485
C	1.05202539	2.24631791	-0.16788531
C	-3.13045053	1.31789551	0.40423748
C	-3.74330677	-0.00261953	0.03792679
C	-4.83719727	-0.44799343	0.79696555
C	-5.47929225	-1.64030311	0.47753416
C	-5.05483350	-2.39070162	-0.62315505
C	-3.98687351	-1.94249333	-1.40143214
C	-3.32865106	-0.75724188	-1.07064194
O	-3.82443412	2.18642142	0.92365961
C	1.61971098	-0.22549691	-0.01867008
C	1.22564046	-1.59803585	0.24526283
C	2.15957563	-2.65564534	0.21795296
O	3.41626296	-2.41933096	0.04047024
O	2.89674571	-0.02365233	-0.30115652
C	1.76423825	-4.09143017	0.38335823
B	3.98095145	-0.95383003	0.06938278
F	4.38750414	-0.75591354	1.36146554
F	4.96327860	-0.90917931	-0.86652861
O	2.36569775	2.49386917	-0.31974284
C	2.81589947	3.83847018	-0.43991888
H	0.37832270	4.28106791	-0.23282860
H	-2.01644096	3.72277402	0.09832121
H	-1.07338693	-0.43514539	0.25184640
H	-5.16810856	0.16028299	1.63200878
H	-6.31573827	-1.98307052	1.07930273
H	-5.56189109	-3.31680310	-0.87794633
H	-3.67007413	-2.51087265	-2.27096137
H	-2.51302510	-0.40210108	-1.69172810
H	0.19700974	-1.85127570	0.45499119
H	0.68377642	-4.21074593	0.48219486
H	2.11172225	-4.67152058	-0.47799676
H	2.25307645	-4.50991033	1.27000091
H	3.89864883	3.77373620	-0.54334841
H	2.38962562	4.32318199	-1.32613093
H	2.56496392	4.42338451	0.45266347

Supplementary Table 37 Optimized T_2 geometry of compound **1** at the TD-B3LYP/G/6-31G(d, p) level of theory in Cartesian coordinates (Å).

Atom	X	Y	Z
C	-0.01848266	3.21886202	0.00464308
C	1.31976565	2.87780990	-0.11737051
C	1.71835688	1.52574101	-0.20549170
C	0.72132084	0.54375661	-0.20732825
C	-0.65043618	0.85100255	-0.09016425
C	-1.01704180	2.23292536	0.02771296
C	3.14831242	1.21324668	-0.34782247
C	3.76687304	-0.06052127	0.03914116
C	4.96914172	-0.45331754	-0.60034381
C	5.60679114	-1.63313835	-0.23964943
C	5.08068063	-2.44072230	0.77511973
C	3.90853737	-2.04768069	1.43579464
C	3.26166713	-0.86925645	1.08427519
O	3.92018786	2.11078105	-0.81518154
C	-1.63721640	-0.22299606	-0.10632071
C	-1.37685420	-1.46853360	-0.74277845
C	-2.31980994	-2.50723058	-0.69785702
O	-3.46589269	-2.34860910	-0.10961348
O	-2.78729020	-0.04029025	0.48414020
C	-2.07831590	-3.85577488	-1.30242671
B	-3.95533991	-0.98694870	0.39271745
F	-4.84495006	-0.51001110	-0.52999860
F	-4.46094607	-1.16775504	1.64338528
O	-2.32840878	2.51541894	0.11363836
C	-2.75596564	3.87256776	0.16882191
H	-0.29067372	4.26382841	0.08134070
H	2.07961472	3.65080038	-0.13315337
H	1.01592723	-0.49470495	-0.28683696
H	5.36838078	0.17577656	-1.38769857
H	6.51767308	-1.92933108	-0.75153003
H	5.58150012	-3.36201686	1.05601387
H	3.50695763	-2.65968636	2.23810702
H	2.37865107	-0.55567574	1.62999686
H	-0.45297794	-1.62670208	-1.28096126
H	-1.08063569	-3.93550940	-1.73775083
H	-2.20033656	-4.63033326	-0.53773435
H	-2.82543776	-4.05328196	-2.07919219
H	-3.84356484	3.83076650	0.21891795
H	-2.36821300	4.37632333	1.06171695
H	-2.44981444	4.42553915	-0.72690347

Supplementary Table 38 Optimized S_0 geometry of compound **2** at the TD-B3LYP/G/6-31G(d, p) level of theory in Cartesian coordinates (Å).

Atom	X	Y	Z
O	4.03259822	0.48552449	-0.14236936
C	4.17928784	1.70148273	-0.07141322
C	5.54092120	2.29289608	0.07232491
C	5.79699865	3.54527582	0.66293614
H	4.97707848	4.14698369	1.04078159
C	7.09518276	4.01177614	0.80760866
H	7.30298004	4.96903601	1.27393338
C	8.17727461	3.23923001	0.35464628
O	9.40629896	3.79112914	0.52416494
C	10.54758214	3.05297178	0.10503485
H	11.40990248	3.67891939	0.33756379
H	10.52455361	2.85441167	-0.97355110
H	10.63278019	2.10273348	0.64604393
C	7.94060737	1.98193676	-0.22328386
H	8.76040012	1.36511163	-0.57135229
C	6.63453597	1.52011804	-0.34581226
H	6.43716136	0.54281549	-0.77370276
C	2.96410880	2.58718492	-0.15406794
C	1.74061625	2.05433432	0.26365014
H	1.72027720	1.05108877	0.67284185
C	0.54036454	2.77061232	0.14401951
C	-0.71018003	2.10615072	0.57962311
O	-0.55225837	1.16198164	1.45227617
B	-1.72625675	0.48900865	2.14906268
F	-1.98961490	1.18396400	3.29814766
F	-1.43265899	-0.82586259	2.31648081
O	-2.93821330	0.60548069	1.24283167
C	-3.03889611	1.54758872	0.36439337
C	-4.37521462	1.64903950	-0.30686215
H	-4.62140960	0.68853908	-0.77105332
H	-4.39448089	2.43485552	-1.06298320
H	-5.14515137	1.84651686	0.44646321
C	-1.97268361	2.38619698	0.03880164
H	-2.08757526	3.16151043	-0.70151803
C	0.58976831	4.07116702	-0.41207379
O	-0.54012352	4.84019463	-0.56221269
C	-0.88239908	5.63355281	0.58283424
H	-1.10905620	5.00338976	1.45051756
H	-1.76990774	6.20479345	0.30623205
H	-0.06777553	6.32237145	0.83661232
C	1.80500651	4.60152961	-0.84613669
H	1.80279294	5.59205707	-1.28927874
C	2.98253551	3.86837306	-0.72603971
H	3.91194182	4.28592824	-1.09801536

Supplementary Table 39 Optimized S_1 geometry of compound **2** at the TD-B3LYP/G/6-31G(d, p) level of theory in Cartesian coordinates (Å).

Atom	X	Y	Z
O	1.83475979	-2.00969174	-0.09817404
C	1.90427520	-0.77819929	-0.12152022
C	3.37883593	-0.45812139	-0.01878046
C	3.93050949	0.02747814	1.19215252
H	3.27181867	0.29332218	2.00978006
C	5.28768881	0.20816932	1.30110418
H	5.74421198	0.62015987	2.19346443
C	6.14310476	-0.13942827	0.21151131
O	7.43384533	0.07542724	0.42665679
C	8.41063221	-0.25004500	-0.58039702
H	9.36957006	0.02877062	-0.14911485
H	8.22586911	0.32711568	-1.48988184
H	8.39268186	-1.32147083	-0.79481983
C	5.60316260	-0.68045526	-0.98349839
H	6.24694312	-0.96081643	-1.80682962
C	4.24212815	-0.86273245	-1.07403993
H	3.80734984	-1.30839163	-1.96063464
C	0.84666091	0.20062821	-0.15775551
C	-0.47784895	-0.24805340	-0.10057967
H	-0.69067576	-1.30720728	-0.02125951
C	-1.57783762	0.63218409	-0.15653308
C	-2.90854553	0.05373908	-0.17714024
O	-2.95161737	-1.25451865	0.08733471
B	-4.24518319	-1.91779694	0.43703041
F	-4.45856841	-1.79388761	1.80285425
F	-4.18172074	-3.23023886	0.02675642
O	-5.37743436	-1.25014455	-0.28701148
C	-5.28218772	0.02078087	-0.62588698
C	-6.56123251	0.62367103	-1.12792873
H	-6.93175913	0.05608403	-1.98961654
H	-6.42925980	1.66747093	-1.42228438
H	-7.33673776	0.56574155	-0.35503377
C	-4.09511036	0.72463039	-0.54321736
H	-4.06149226	1.76109978	-0.84250201
C	-1.24823847	2.03036382	-0.20329587
O	-2.21733230	3.00780672	-0.17777353
C	-2.80828278	3.22268808	1.11205077
H	-3.27973487	2.31277816	1.49488153
H	-3.56849234	3.99419040	0.97575234
H	-2.05191028	3.57596705	1.82442963
C	0.07257029	2.47194090	-0.28160340
H	0.23488105	3.54372366	-0.33603169
C	1.14251015	1.58288922	-0.27170409
H	2.15975719	1.94878560	-0.34464110

Supplementary Table 40 Optimized T_1 geometry of compound **2** at the TD-B3LYP/G/6-31G(d, p) level of theory in Cartesian coordinates (Å).

Atom	X	Y	Z
O	1.74120452	-1.69051244	-1.57282728
C	1.96244851	-0.70830648	-0.87191526
C	3.33644807	-0.45265437	-0.34572151
C	3.60476234	0.29686312	0.81538886
H	2.78922879	0.74684525	1.37162938
C	4.90182902	0.44039380	1.28642233
H	5.11643164	1.00315280	2.18882119
C	5.97147456	-0.15827456	0.60117568
O	7.20376734	0.04999339	1.13518060
C	8.32688853	-0.55439387	0.50742150
H	9.19378175	-0.26619236	1.10339315
H	8.45829110	-0.19273718	-0.52003210
H	8.24218696	-1.64799314	0.49674731
C	5.72067410	-0.91692342	-0.55307035
H	6.52951895	-1.39339749	-1.09395753
C	4.41308417	-1.06501643	-1.00391307
H	4.20078974	-1.66647498	-1.88166003
C	0.83603897	0.23607844	-0.55109146
C	-0.44883725	-0.28533290	-0.48792702
H	-0.59033380	-1.34801189	-0.63635004
C	-1.59820038	0.51815498	-0.24460493
C	-2.86604128	-0.14377908	-0.19133114
O	-2.87521092	-1.46763056	-0.32586665
B	-4.01503138	-2.32752319	0.08268061
F	-3.95022380	-2.61112444	1.42183483
F	-4.08204154	-3.40389770	-0.74263084
O	-5.34240584	-1.53818078	-0.11546892
C	-5.37017056	-0.24086205	-0.12490078
C	-6.71958823	0.40581617	-0.14802642
H	-7.28680722	0.06106516	-1.02048937
H	-6.64718152	1.49486721	-0.18161456
H	-7.29336124	0.11443984	0.73983814
C	-4.15786555	0.50173429	-0.11470214
H	-4.20477150	1.58003295	-0.08027757
C	-1.36836227	1.93467447	-0.07803238
O	-2.45399544	2.71503597	0.18833418
C	-2.27242505	4.11094034	0.40214598
H	-1.60663998	4.29837969	1.25211350
H	-3.26333285	4.50942512	0.62310239
H	-1.87295049	4.60402078	-0.49161889
C	-0.08051744	2.45713480	-0.17639457
H	0.08110745	3.52260895	-0.07297060
C	1.01842631	1.62692472	-0.40494829
H	2.00581268	2.06493704	-0.49628593

Supplementary Table 41 Optimized T_2 geometry of compound **2** at the TD-B3LYP/G/6-31G(d, p) level of theory in Cartesian coordinates (Å).

Atom	X	Y	Z
O	1.73941087	-1.47374523	-1.49712686
C	1.99468213	-0.39789888	-0.82263343
C	3.36306117	-0.22308601	-0.40506255
C	3.90026717	0.90130128	0.31700079
H	3.24502992	1.69629179	0.64561838
C	5.22285449	0.94615215	0.66766570
H	5.62425004	1.77224276	1.24640412
C	6.12122324	-0.10745553	0.29923390
O	7.39996740	0.07110130	0.69100822
C	8.36866478	-0.92773620	0.37228906
H	9.31590453	-0.55920828	0.76585867
H	8.44888969	-1.06777164	-0.71139861
H	8.11970492	-1.88260675	0.84887638
C	5.62259170	-1.23640048	-0.39002307
H	6.27397123	-2.06697327	-0.63723400
C	4.28978676	-1.29930364	-0.72143605
H	3.87738047	-2.17042206	-1.21342985
C	0.87760511	0.52684790	-0.55566149
C	-0.40737192	-0.03345539	-0.43143825
H	-0.51839640	-1.10796601	-0.50814264
C	-1.55285074	0.74451496	-0.21710848
C	-2.85148869	0.04389580	-0.11008085
O	-2.77808751	-1.20568250	0.22640447
B	-3.99696645	-2.04943695	0.55596255
F	-4.18064423	-2.01585034	1.91271330
F	-3.81734602	-3.29006136	0.02858693
O	-5.20735822	-1.41612284	-0.10247537
C	-5.22212565	-0.17226349	-0.45573042
C	-6.54872371	0.33102622	-0.94006176
H	-6.88374428	-0.28376766	-1.78177193
H	-6.50071673	1.37586375	-1.24947149
H	-7.29314545	0.21762127	-0.14512850
C	-4.08641304	0.63325538	-0.42001266
H	-4.13299773	1.66715529	-0.72115811
C	-1.40722160	2.15001857	-0.12528838
O	-2.48318062	2.98683289	0.08296923
C	-2.77208537	3.25334232	1.45944994
H	-3.06398200	2.33869431	1.98916401
H	-3.60387961	3.96006835	1.47012736
H	-1.90600624	3.70074587	1.96196650
C	-0.14411973	2.72158329	-0.27271726
H	-0.06475549	3.80350193	-0.23822226
C	0.97989811	1.93358562	-0.49652905
H	1.92636372	2.42587149	-0.68435617

Supplementary Table 42 Optimized S_0 geometry of compound **3** at the B3LYP/G/6-31G(d, p) level of theory in Cartesian coordinates (Å).

Atom	X	Y	Z
O	-0.46119220	3.37461461	-0.05571081
C	0.06620651	2.31594632	0.27803565
C	-0.78552833	1.17025353	0.71913943
C	-2.11502221	1.16417555	0.29311779
H	-2.42722405	1.94731213	-0.38815514
C	-3.02483011	0.16141544	0.66033889
C	-4.33449626	0.15359281	-0.02149518
O	-4.77532532	-0.99980268	-0.40713803
B	-6.14781129	-1.20610024	-1.03614116
F	-6.00728547	-2.00808891	-2.12165196
F	-6.97564866	-1.69973798	-0.05631368
O	-6.68373720	0.13642878	-1.48313601
C	-6.19543073	1.26256945	-1.07388913
C	-6.96059991	2.47714025	-1.50136880
H	-7.02147948	2.49842431	-2.59424966
H	-6.49821381	3.39840743	-1.14516935
H	-7.98659563	2.41205459	-1.12490110
C	-5.03811759	1.33388000	-0.30290605
H	-4.67264076	2.28868658	0.04575450
C	-2.58784623	-0.84754141	1.56408755
O	-3.33739426	-1.82973711	2.10027481
C	-4.72592062	-1.64678437	2.41967264
H	-4.94539894	-0.58999695	2.60648859
H	-4.89183742	-2.21503078	3.33698542
H	-5.36928154	-2.02023666	1.62453897
C	-1.24839604	-0.84170417	1.99098531
H	-0.94267777	-1.61681650	2.68460863
C	-0.36121118	0.13627544	1.57550738
H	0.65317765	0.11848406	1.95770330
C	1.56037028	2.20380032	0.23697866
C	2.30332641	3.39254540	0.30886005
H	1.76956709	4.32954097	0.42585756
C	3.68715965	3.37754040	0.23064401
H	4.23204929	4.30920970	0.31116887
C	4.37810123	2.16915200	0.04584495
O	5.72974907	2.10659911	-0.01671853
C	6.49654203	3.29837750	0.14730419
H	6.31044311	3.75889419	1.12351387
H	6.28652620	4.02188538	-0.64812400
H	7.53903003	2.98582423	0.08709385
C	3.65098263	0.95365926	-0.06308385
C	4.25786075	-0.38143990	-0.26512619
O	3.46262951	-1.37100533	0.00485308
B	3.85262115	-2.83935145	-0.04418323
F	3.97531230	-3.28837276	1.23994869
F	2.92985750	-3.49785229	-0.79752105
O	5.20298318	-2.95500490	-0.72359603
C	5.94801361	-1.93479199	-0.99214526
C	7.27872712	-2.26933636	-1.59578440
H	7.12632948	-2.84991549	-2.51122631
H	7.84061511	-2.90506215	-0.90373323
H	7.86102071	-1.37572155	-1.82347649
C	5.54804300	-0.62020988	-0.75543444
H	6.20509070	0.19726263	-0.99573469
C	2.25061286	1.00666422	0.04430482
H	1.71434630	0.07270082	-0.05963579

Supplementary Table 43 Optimized S_1 geometry of compound **3** at the TD-B3LYP/G/6-31G(d, p) level of theory in Cartesian coordinates (Å).

Atom	X	Y	Z
O	-0.50891633	3.56708765	0.49025072
C	0.16933393	2.53968298	0.42670410
C	-0.66866560	1.34525984	0.85809543
C	-1.98354240	1.26336799	0.32600810
H	-2.26446717	1.98208898	-0.43404817
C	-2.88234475	0.28043137	0.69957459
C	-4.15829545	0.18025693	-0.05998203
O	-4.43358969	-0.99194946	-0.52804889
B	-5.78242307	-1.30674460	-1.18536165
F	-5.56369519	-2.07195829	-2.27287074
F	-6.57370002	-1.85577754	-0.20782233
O	-6.41073815	0.00730770	-1.62037765
C	-6.06469375	1.15328781	-1.15192454
C	-6.90286201	2.31219866	-1.58726998
H	-6.88660399	2.37411250	-2.68032621
H	-6.55437220	3.25427182	-1.16341282
H	-7.94280597	2.13662360	-1.29358200
C	-4.95470260	1.29524588	-0.30107418
H	-4.69785528	2.26102477	0.11023028
C	-2.46720794	-0.64075572	1.72232665
O	-3.18770822	-1.56710404	2.32140847
C	-4.62915155	-1.69348057	2.27420190
H	-5.09694805	-0.70765969	2.25415795
H	-4.88273745	-2.21736532	3.19405617
H	-4.92678772	-2.27013596	1.40114701
C	-1.14093413	-0.55320446	2.25673002
H	-0.86054037	-1.29082157	2.99948486
C	-0.27375898	0.44028696	1.86212967
H	0.71953102	0.49575233	2.28730606
C	1.57654961	2.44442445	0.08689663
C	2.33910385	3.62409662	-0.10025937
H	1.88191220	4.60034057	-0.00108739
C	3.69197999	3.47560717	-0.38289422
H	4.33046535	4.34325180	-0.51815327
C	4.29412206	2.21803936	-0.48869261
O	5.64901004	2.19254872	-0.74015423
C	6.45820719	2.03801641	0.43247545
H	6.23829447	1.09711534	0.94791472
H	6.30615350	2.87913776	1.12084905
H	7.49571310	2.03016745	0.09230056
C	3.53966714	1.01137988	-0.34406635
C	4.01917888	-0.35210182	-0.44183591
O	3.11582975	-1.27588367	-0.07960805
B	3.53457152	-2.66624033	0.25556533
F	3.86341629	-2.72613297	1.60687258
F	2.48617571	-3.51050042	-0.05606122
O	4.72887414	-3.05531518	-0.55341546
C	5.56086732	-2.12653574	-0.99368201
C	6.82515282	-2.66777658	-1.59220308
H	6.58984152	-3.34729292	-2.41975538
H	7.37644186	-3.25525199	-0.84844888
H	7.47090197	-1.86886504	-1.96400946
C	5.27525165	-0.77603199	-0.92069949
H	5.98079393	-0.05280929	-1.30048236
C	2.16849282	1.18946688	-0.05278203
H	1.59510165	0.27748413	0.04236633

Supplementary Table 44 Optimized T_1 geometry of compound **3** at the TD-B3LYP/G/6-31G(d, p) level of theory in Cartesian coordinates (Å).

Atom	X	Y	Z
O	-0.46921732	3.35382004	-0.09779947
C	0.05672000	2.29944261	0.25187845
C	-0.79485599	1.15757273	0.70120792
C	-2.12771437	1.15395209	0.28583543
H	-2.44293748	1.93611969	-0.39514603
C	-3.03693756	0.15420618	0.66265855
C	-4.35195303	0.14737368	-0.00820289
O	-4.80156640	-1.00661681	-0.38194673
B	-6.18059463	-1.21083946	-0.99699237
F	-6.05423298	-2.02326292	-2.07650322
F	-7.00245177	-1.69162577	-0.00556284
O	-6.71326630	0.13043119	-1.45092456
C	-6.21625557	1.25727644	-1.05384471
C	-6.97878157	2.47229980	-1.48487947
H	-7.04642751	2.48629585	-2.57748874
H	-6.50994884	3.39391741	-1.13813576
H	-8.00265848	2.41450971	-1.10153393
C	-5.05287944	1.32883777	-0.29243817
H	-4.68037700	2.28453792	0.04612310
C	-2.59457262	-0.85393186	1.56499271
O	-3.34285097	-1.83251788	2.11015797
C	-4.72628808	-1.64143492	2.44595264
H	-4.93808695	-0.58299309	2.63234665
H	-4.88424198	-2.20617417	3.36689065
H	-5.38131367	-2.01362254	1.65979525
C	-1.25144167	-0.85138454	1.97954421
H	-0.94167456	-1.62656460	2.67131765
C	-0.36529131	0.12369087	1.55498846
H	0.65355121	0.10459370	1.92445812
C	1.55237025	2.19275537	0.21673914
C	2.28935900	3.39591597	0.31574950
H	1.75417194	4.32809332	0.45189196
C	3.67774465	3.37503826	0.23492772
H	4.22349557	4.30457432	0.33443762
C	4.36839105	2.18057611	0.02778608
O	5.72602471	2.12520704	-0.03964940
C	6.48299519	3.32509748	0.10271521
H	6.31214582	3.78746550	1.08097694
H	6.24429016	4.04256320	-0.69001262
H	7.52839940	3.02692296	0.02019055
C	3.65023267	0.93313894	-0.11454417
C	4.22060402	-0.35451548	-0.34347786
O	3.38462590	-1.38891980	-0.44120186
B	3.80733315	-2.79224069	-0.20763137
F	3.84838721	-3.06872088	1.13467177
F	3.03619152	-3.62441714	-0.95221960
O	5.26930660	-2.96610593	-0.71815999
C	6.08573823	-1.96134540	-0.79286767
C	7.50948317	-2.27350076	-1.13036740
H	7.56158638	-2.78979717	-2.09582190
H	7.93214540	-2.95413293	-0.38235141
H	8.12295888	-1.37175216	-1.17848996
C	5.62081194	-0.63981278	-0.55996639
H	6.32889412	0.17502804	-0.57215135
C	2.22740407	1.00368741	-0.00529212
H	1.68754085	0.07605742	-0.13603178

Supplementary Table 45 Optimized T_2 geometry of compound **3** at the TD-B3LYP/G/6-31G(d, p) level of theory in Cartesian coordinates (Å).

Atom	X	Y	Z
O	-0.48582569	3.27602050	-0.06418731
C	0.05111409	2.21306679	0.24432245
C	-0.80403111	1.05341317	0.65087670
C	-2.13721488	1.10204180	0.25488603
H	-2.42754554	1.92657702	-0.38494947
C	-3.07837405	0.10327664	0.58048711
C	-4.38136460	0.13184895	-0.05823522
O	-4.99140867	-1.01045415	-0.27637069
B	-6.41764615	-1.12507047	-0.74172206
F	-6.48247228	-2.04342770	-1.74324258
F	-7.19883255	-1.42182276	0.35514048
O	-6.89140897	0.21032692	-1.29400388
C	-6.25116156	1.32675252	-1.10100455
C	-6.92958084	2.55968081	-1.61072726
H	-7.11512069	2.46155117	-2.68606313
H	-6.33376089	3.45640676	-1.43194082
H	-7.90667940	2.67420914	-1.12835465
C	-4.99957872	1.34453465	-0.46563025
H	-4.51797735	2.29493381	-0.27956307
C	-2.62393053	-0.96162406	1.44577982
O	-3.39291142	-1.92536441	1.98049796
C	-4.72232502	-1.66181803	2.46570978
H	-4.85815534	-0.59515713	2.66688489
H	-4.80733123	-2.22313731	3.39876469
H	-5.47123015	-1.99518322	1.74955986
C	-1.26753563	-1.02503916	1.81234688
H	-0.96638109	-1.84379350	2.45641914
C	-0.36756671	-0.04487233	1.43708329
H	0.65185876	-0.09819764	1.79835764
C	1.54692906	2.12924047	0.21780923
C	2.25329362	3.34477208	0.30581660
H	1.68962617	4.26368782	0.42035264
C	3.63840646	3.36932644	0.24257078
H	4.15381661	4.31679076	0.33228433
C	4.36745584	2.18538326	0.06824250
O	5.72201964	2.16878714	0.02929042
C	6.44433812	3.38870044	0.18558586
H	6.23321300	3.85280539	1.15509662
H	6.21367763	4.09497172	-0.61957544
H	7.49822854	3.11414618	0.13700567
C	3.68159240	0.93628614	-0.05474755
C	4.32231114	-0.36748343	-0.22956638
O	3.52989264	-1.39972764	-0.09272409
B	3.98630190	-2.83958921	-0.05417846
F	4.03138888	-3.24617082	1.25067637
F	3.17060629	-3.57468893	-0.85699760
O	5.39967245	-2.92514570	-0.61765841
C	6.14895436	-1.88423777	-0.79176150
C	7.54655767	-2.17002472	-1.24986621
H	7.51652572	-2.73927996	-2.18502176
H	8.05431084	-2.79845876	-0.51017798
H	8.12183034	-1.25555620	-1.40230720
C	5.67772513	-0.58106636	-0.57741328
H	6.33938677	0.25630512	-0.72118290
C	2.27046029	0.95464166	0.02747780
H	1.76403972	0.00677409	-0.09505286

Supplementary Table 46 Optimized T_3 geometry of compound **3** at the TD-B3LYP/G/6-31G(d, p) level of theory in Cartesian coordinates (Å).

Atom	X	Y	Z
O	-0.46618357	3.36473384	-0.07158171
C	0.06047441	2.30814223	0.27158104
C	-0.79368821	1.16603942	0.71842495
C	-2.12359863	1.15976266	0.29438651
H	-2.43502127	1.93943131	-0.39118619
C	-3.03461255	0.16039893	0.66883952
C	-4.34520589	0.14998778	-0.01009872
O	-4.79384064	-1.00607911	-0.37877801
B	-6.16879057	-1.21337163	-1.00120435
F	-6.03597995	-2.03268424	-2.07496325
F	-6.99729433	-1.68812508	-0.01225206
O	-6.69819718	0.12500473	-1.46679827
C	-6.20255853	1.25417124	-1.07415032
C	-6.96186020	2.46680400	-1.51753996
H	-7.02296165	2.47384044	-2.61060513
H	-6.49453613	3.39037792	-1.17396170
H	-7.98802763	2.41207875	-1.13991973
C	-5.04370545	1.33005528	-0.30661002
H	-4.67262757	2.28777107	0.02776850
C	-2.59768756	-0.84380518	1.57793624
O	-3.34940572	-1.82167025	2.12091278
C	-4.73307587	-1.62647581	2.45306807
H	-4.94279311	-0.56708261	2.63652761
H	-4.89497408	-2.18862654	3.37496635
H	-5.38719837	-1.99885733	1.66619776
C	-1.25784313	-0.83794817	2.00220007
H	-0.95248449	-1.60950634	2.69999346
C	-0.36954651	0.13637108	1.57962790
H	0.64670996	0.12083862	1.95643935
C	1.55417283	2.19788880	0.23042254
C	2.29813020	3.40252026	0.31735613
H	1.76891189	4.33787683	0.45136987
C	3.69072897	3.37250044	0.22453265
H	4.23996476	4.30114896	0.31396870
C	4.37501638	2.17948431	0.01874913
O	5.73496702	2.11568114	-0.06399035
C	6.49421566	3.31426035	0.06508692
H	6.33401875	3.78236007	1.04269402
H	6.24818276	4.02915483	-0.72803568
H	7.53897224	3.01593459	-0.02680113
C	3.64901489	0.92616193	-0.10994311
C	4.20718045	-0.35932065	-0.33576964
O	3.37135943	-1.39028712	-0.43406653
B	3.78277512	-2.79503313	-0.21335828
F	3.83384180	-3.08848283	1.12319850
F	3.01506541	-3.61943665	-0.96728391
O	5.25325701	-2.96793981	-0.72894441
C	6.07059391	-1.96839434	-0.78485125
C	7.49661288	-2.28526514	-1.11599339
H	7.54628177	-2.80709066	-2.07768135
H	7.91122910	-2.96292244	-0.36168939
H	8.11292827	-1.38595868	-1.16648223
C	5.61636952	-0.65321508	-0.54334097
H	6.32630382	0.15887753	-0.54333743
C	2.22029125	1.00565475	0.01002328
H	1.67433825	0.08071469	-0.11335927

Supplementary Table 47 Optimized S_0 geometry of compound **1** at the B3LYP/G/6-31G(d, p) level of theory in Cartesian coordinates (Å). (PCM: dichloromethane)

Atom	X	Y	Z
C	-2.63847562	-2.27138636	0.46432135
C	-1.26633327	-2.22183060	0.28128098
C	-0.60717290	-1.01684840	0.02359393
C	-1.35993496	0.15754744	-0.00949815
C	-2.73684512	0.13997585	0.20204879
C	-3.39371235	-1.09232242	0.42452653
C	0.87630073	-1.02148335	-0.12550478
C	1.54847195	0.09818807	-0.85995200
C	2.79897283	0.53147576	-0.40865286
C	3.47006309	1.54791245	-1.07822003
C	2.91017239	2.11559418	-2.22230776
C	1.67818998	1.66755242	-2.69401527
C	0.99284834	0.66699498	-2.01094009
O	1.54309233	-1.93050621	0.34977975
C	-3.46998453	1.42380146	0.18751520
C	-3.17583727	2.40960649	-0.75037771
C	-3.76994701	3.66124583	-0.61400330
O	-4.65725516	3.89734098	0.29460636
O	-4.35147497	1.60259572	1.11551086
C	-3.43698624	4.80798424	-1.50439703
B	-5.25308214	2.80143177	1.13501873
F	-5.34012739	3.25395707	2.42820889
F	-6.47332458	2.45093438	0.59847626
O	-4.72579730	-1.05175699	0.56061623
C	-5.43570247	-2.27774384	0.71603484
H	-3.11791030	-3.22746965	0.63549554
H	-0.68424079	-3.13733708	0.32612708
H	-0.87381065	1.11708446	-0.15551456
H	3.22725463	0.07209070	0.47726527
H	4.42978776	1.89872434	-0.71047187
H	3.43754945	2.90573011	-2.74912490
H	1.24937163	2.09646004	-3.59475343
H	0.03622221	0.31391696	-2.38613804
H	-2.44808360	2.23120819	-1.52985706
H	-2.49267206	4.65580848	-2.02624137
H	-4.23521465	4.90598617	-2.24960916
H	-3.41749352	5.73354806	-0.92549334
H	-6.48795327	-2.00145120	0.76497814
H	-5.26717526	-2.93834733	-0.14042654
H	-5.14584993	-2.78571319	1.64119451

Supplementary Table 48 Optimized S_1 geometry of compound **1** at the TD-B3LYP/G/6-31G(d, p) level of theory in Cartesian coordinates (Å). (PCM: dichloromethane)

Atom	X	Y	Z
C	-2.56437708	-2.14447836	0.15966941
C	-1.19343004	-2.10004957	0.14986365
C	-0.51253777	-0.85147645	0.12410751
C	-1.24123744	0.35910931	0.20536895
C	-2.62068810	0.36492313	0.21519155
C	-3.29294083	-0.91919498	0.19520537
C	0.99135058	-0.87695515	0.16428079
C	1.80904220	0.03126568	-0.66419130
C	3.16737197	0.19636071	-0.33559606
C	3.97578635	1.02104635	-1.10976562
C	3.44107622	1.67537292	-2.22653085
C	2.09727214	1.50342469	-2.56849362
C	1.27889943	0.68721995	-1.78889773
O	1.47300834	-1.68061571	0.96126582
C	-3.40705925	1.62478081	0.28369586
C	-3.65999196	2.44398836	-0.81094800
C	-4.36463902	3.62773175	-0.64589244
O	-4.83386818	3.97126614	0.57473391
O	-3.84753240	1.92950582	1.52755832
C	-4.63403747	4.62376224	-1.72907972
B	-4.93531272	2.93642058	1.62271826
F	-4.82842898	3.55894056	2.86655303
F	-6.17564052	2.28064383	1.51358024
O	-4.60215042	-0.85255800	0.21924417
C	-5.43213273	-2.04294686	0.23330470
H	-3.08381747	-3.09392979	0.13349361
H	-0.61208476	-3.01437434	0.13698549
H	-0.71304991	1.30496141	0.23856470
H	3.56628681	-0.32074091	0.53063138
H	5.02069248	1.15650268	-0.84854380
H	4.07452348	2.31754956	-2.83131971
H	1.68756167	2.00294831	-3.44061550
H	0.24002241	0.54704667	-2.06943191
H	-3.28630941	2.16937522	-1.79020951
H	-4.20255680	4.29943983	-2.67923897
H	-5.71239362	4.77246344	-1.87144083
H	-4.21125167	5.60493593	-1.47646990
H	-6.45231164	-1.66812731	0.25802803
H	-5.25398532	-2.62468469	-0.67296669
H	-5.21322860	-2.63035376	1.12668742

Supplementary Table 49 Optimized T_1 geometry of compound **1** at the TD-B3LYP/G/6-31G(d, p) level of theory in Cartesian coordinates (Å). (PCM: dichloromethane)

Atom	X	Y	Z
C	-2.58038086	-2.26099294	0.57786635
C	-1.20459099	-2.16203022	0.43699702
C	-0.61422724	-0.91325899	0.10173109
C	-1.40852334	0.21104889	-0.03047321
C	-2.83014692	0.18093296	0.14446795
C	-3.40345599	-1.13772609	0.43287612
C	0.88024993	-0.84034016	0.02641459
C	1.55512585	0.15150213	-0.86450558
C	2.85033239	0.57508146	-0.51890476
C	3.54538981	1.45878613	-1.33939534
C	2.96468181	1.91129399	-2.52980684
C	1.68746371	1.47848911	-2.89346851
C	0.98008990	0.60779320	-2.06283732
O	1.54793151	-1.61075818	0.71660913
C	-3.60242802	1.37389876	0.04328874
C	-3.02325976	2.61811734	-0.38121791
C	-3.80129785	3.79154505	-0.50660016
O	-5.05278685	3.79520692	-0.13440923
O	-4.92262722	1.36935343	0.27380268
C	-3.26458909	5.07053674	-1.05851491
B	-5.66359339	2.59454818	0.58416312
F	-5.61552315	2.87848515	1.94146031
F	-6.95566030	2.46787396	0.12002056
O	-4.73761840	-1.19469173	0.53573918
C	-5.37919863	-2.45041245	0.79999027
H	-3.02275588	-3.22323010	0.80138258
H	-0.57010491	-3.03073393	0.56699037
H	-0.92333245	1.15640316	-0.22574602
H	3.29367247	0.20504759	0.39977316
H	4.53955706	1.79269249	-1.05734967
H	3.50988281	2.59525057	-3.17390543
H	1.24196503	1.81538118	-3.82475849
H	-0.00434705	0.26252247	-2.36035054
H	-1.98221397	2.68762706	-0.66235637
H	-2.22309016	4.96862161	-1.36716457
H	-3.86522431	5.38607444	-1.91976160
H	-3.34090882	5.86451670	-0.30584673
H	-6.44552371	-2.22921971	0.82326936
H	-5.16307508	-3.17135502	0.00542421
H	-5.05995795	-2.85338115	1.76620639

Supplementary Table 50 Optimized T_2 geometry of compound **1** at the TD-B3LYP/G/6-31G(d, p) level of theory in Cartesian coordinates (Å). (PCM: dichloromethane)

Atom	X	Y	Z
C	-2.59543710	-2.24822649	0.52956245
C	-1.22745655	-2.16863611	0.34214394
C	-0.59618105	-0.93045441	0.06417598
C	-1.39316742	0.22441996	0.04034352
C	-2.78846587	0.18034220	0.22139899
C	-3.39977605	-1.09452339	0.46647757
C	0.85736283	-0.89473750	-0.10693165
C	1.59444758	0.11446761	-0.86106889
C	2.96497686	0.33202684	-0.56765107
C	3.69577339	1.28050678	-1.27388809
C	3.09238554	2.02491043	-2.29434735
C	1.74546630	1.79850740	-2.61249827
C	1.00531743	0.84876534	-1.91848815
O	1.53383461	-1.83059633	0.46229719
C	-3.55367883	1.42421568	0.16184812
C	-3.12207385	2.52901707	-0.60455011
C	-3.88825063	3.69700791	-0.64794654
O	-5.00573129	3.80360782	0.02207637
O	-4.67053067	1.50083953	0.84439131
C	-3.50576043	4.89740183	-1.44996608
B	-5.54390265	2.70810984	0.89601807
F	-5.57369343	3.17167207	2.19651392
F	-6.79947467	2.36536791	0.43631796
O	-4.73409903	-1.12893254	0.58546826
C	-5.39940532	-2.38380036	0.78928047
H	-3.04865840	-3.21474026	0.70762830
H	-0.62664366	-3.07052873	0.38165273
H	-0.91622417	1.18973300	-0.07000113
H	3.42681314	-0.23010874	0.23653855
H	4.73931278	1.44847017	-1.02390480
H	3.66378228	2.76897924	-2.84054832
H	1.27534343	2.36032341	-3.41449025
H	-0.02399927	0.65946339	-2.20232047
H	-2.22208352	2.47188641	-1.20005759
H	-2.58628034	4.72945458	-2.01167607
H	-4.31543029	5.15353011	-2.14201526
H	-3.36924927	5.75594913	-0.78236983
H	-6.45944479	-2.13981416	0.84964154
H	-5.22089390	-3.06186172	-0.05114914
H	-5.07292372	-2.85151489	1.72319380

Supplementary Table 51 Optimized S_0 geometry of compound **2** at the B3LYP/G/6-31G(d, p) level of theory in Cartesian coordinates (Å). (PCM: dichloromethane)

Atom	X	Y	Z
O	4.02694937	0.46831807	-0.33004785
C	4.15387758	1.67769549	-0.12222164
C	5.50529449	2.26590994	0.08892456
C	5.73794361	3.41802796	0.86669151
H	4.90869643	3.93641788	1.33625090
C	7.02720719	3.88432368	1.07851372
H	7.21220813	4.76217862	1.68920110
C	8.12436324	3.21777959	0.50449527
O	9.34153721	3.76047205	0.75520174
C	10.50017897	3.12951884	0.20255193
H	11.34867527	3.73313284	0.52566582
H	10.45814488	3.11288356	-0.89204007
H	10.61319450	2.10713448	0.57961251
C	7.91107932	2.06280259	-0.26706562
H	8.74129043	1.53086605	-0.71573754
C	6.61407290	1.59560150	-0.45316696
H	6.44452453	0.69691273	-1.03734434
C	2.93421758	2.54724699	-0.09354000
C	1.71347838	1.96185899	0.25157026
H	1.69920947	0.91521441	0.52875597
C	0.50510781	2.67332571	0.24085332
C	-0.71875853	1.94464630	0.62210378
O	-0.50631454	0.84546949	1.29537756
B	-1.58301940	-0.07358276	1.78638111
F	-1.55802039	-0.07836069	3.16538523
F	-1.36908208	-1.32629954	1.25216377
O	-2.92616451	0.41208631	1.33536699
C	-3.08911705	1.50431854	0.64746217
C	-4.50507607	1.81560865	0.28475010
H	-4.92845779	0.97708461	-0.27863083
H	-4.57926683	2.72916307	-0.30545188
H	-5.09854881	1.92173695	1.19960287
C	-2.02299104	2.31852968	0.27603769
H	-2.20558348	3.21366093	-0.29351520
C	0.53572409	4.04792547	-0.14128958
O	-0.62332733	4.73457136	-0.11021698
C	-0.62694642	6.13461292	-0.42448758
H	0.02327839	6.68840622	0.25947696
H	-1.65974709	6.45643385	-0.29456724
H	-0.31358805	6.30524094	-1.45902465
C	1.75587580	4.63414982	-0.51026821
H	1.78959314	5.67036806	-0.82078636
C	2.93343857	3.89589672	-0.48539014
H	3.85454817	4.37461114	-0.79950417

Supplementary Table 52 Optimized S_1 geometry of compound **2** at the TD-B3LYP/G/6-31G(d, p) level of theory in Cartesian coordinates (Å). (PCM: dichloromethane)

Atom	X	Y	Z
O	4.17694578	0.48674011	0.54922123
C	4.14521255	1.70429332	0.34790661
C	5.52499369	2.30552742	0.39777176
C	5.85527449	3.32009572	1.33061796
H	5.08571050	3.73742379	1.96867503
C	7.14223216	3.79182547	1.39508633
H	7.43007909	4.59004251	2.06877784
C	8.14940985	3.23055281	0.54383114
O	9.34485443	3.75979264	0.68267203
C	10.47246253	3.28543461	-0.09776003
H	11.31084887	3.89957537	0.22162225
H	10.27146145	3.43056261	-1.16066139
H	10.65782956	2.23339720	0.12562878
C	7.83363213	2.17598820	-0.35934932
H	8.59552481	1.74484971	-0.99506367
C	6.54363801	1.70903897	-0.40009928
H	6.27793260	0.89248226	-1.06061845
C	2.94494067	2.50287044	0.14729853
C	1.69470676	1.90205785	0.35570441
H	1.65542661	0.87088044	0.67895019
C	0.47502457	2.58957083	0.18292551
C	-0.75679632	1.87575659	0.43425407
O	-0.59313597	0.55781981	0.69915000
B	-1.66796063	-0.22343876	1.33492560
F	-1.54015184	-0.14727377	2.73339138
F	-1.54691507	-1.54964772	0.91621503
O	-3.01007901	0.26245105	0.94681637
C	-3.15536337	1.56195278	0.65282700
C	-4.58130208	2.01841474	0.58462174
H	-5.13711422	1.42876469	-0.15486829
H	-4.64999425	3.07395471	0.31295306
H	-5.08182421	1.86927625	1.54943074
C	-2.07801685	2.38651659	0.42508655
H	-2.25206008	3.42737245	0.20929832
C	0.58891058	3.96119612	-0.25319284
O	-0.57155778	4.63549516	-0.44655814
C	-0.53375615	5.99236648	-0.89173746
H	-0.01449473	6.63257229	-0.17043785
H	-1.57537998	6.30375913	-0.97281964
H	-0.04883033	6.07487878	-1.87043793
C	1.83465753	4.55299274	-0.47185077
H	1.89035435	5.58005855	-0.80874765
C	3.02076593	3.84225819	-0.27260321
H	3.97160338	4.32340084	-0.47075354

Supplementary Table 53 Optimized T_1 geometry of compound **2** at the TD-B3LYP/G/6-31G(d, p) level of theory in Cartesian coordinates (Å). (PCM: dichloromethane)

Atom	X	Y	Z
O	4.07839713	0.54588846	-0.88409136
C	4.17609311	1.70280940	-0.46919638
C	5.49695768	2.25596663	-0.06997139
C	5.64791367	3.31734068	0.84535006
H	4.77492919	3.78860050	1.28428883
C	6.90808474	3.75211519	1.22758624
H	7.02976763	4.55967422	1.94224998
C	8.05846496	3.14370558	0.69407043
O	9.24241978	3.64916526	1.11774088
C	10.45323968	3.07173001	0.62066835
H	11.26256889	3.62944008	1.09242097
H	10.52357859	3.17420349	-0.46774934
H	10.52938622	2.01370918	0.89423396
C	7.92631870	2.07927794	-0.21442021
H	8.79827972	1.59415528	-0.63601369
C	6.65612981	1.64299686	-0.57437799
H	6.54718098	0.81469359	-1.26679798
C	2.93782278	2.55022660	-0.37792895
C	1.73483765	1.92075858	-0.10368915
H	1.72905373	0.85531514	0.08316054
C	0.49535166	2.62179046	-0.02589611
C	-0.67122531	1.87969634	0.32084312
O	-0.54307392	0.54453658	0.41746835
B	-1.50034322	-0.29129052	1.15552862
F	-1.15370416	-0.38269338	2.49471993
F	-1.58015602	-1.52479998	0.54790111
O	-2.89846484	0.31654024	1.10633591
C	-3.07370795	1.59991365	0.92598333
C	-4.46413789	2.11737384	1.08708579
H	-5.13881321	1.59860225	0.39557314
H	-4.51643336	3.19057184	0.89722126
H	-4.82598643	1.91391065	2.10235174
C	-1.97335053	2.42769514	0.59121936
H	-2.12714227	3.49460102	0.53394559
C	0.54222038	4.04437942	-0.33563496
O	-0.63196962	4.70293190	-0.35456125
C	-0.65831055	6.11457684	-0.61480331
H	-0.07294988	6.65831247	0.13265066
H	-1.70706521	6.40122916	-0.54751547
H	-0.27738133	6.32907814	-1.61778552
C	1.76020516	4.66736562	-0.62248571
H	1.78253384	5.72379598	-0.85709859
C	2.94847940	3.94497558	-0.63944265
H	3.87370061	4.44903409	-0.89303815

Supplementary Table 54 Optimized T_2 geometry of compound **2** at the TD-B3LYP/G/6-31G(d, p) level of theory in Cartesian coordinates (Å). (PCM: dichloromethane)

Atom	X	Y	Z
O	4.10539660	0.46926658	0.08903889
C	4.17697210	1.74792893	0.08438155
C	5.51397519	2.31229539	0.24103335
C	5.77368009	3.55857712	0.88246023
H	4.95335966	4.13649997	1.29306779
C	7.05952177	4.03825366	0.99920362
H	7.26112363	4.99475834	1.47124320
C	8.15851585	3.27935558	0.51688845
O	9.37056915	3.85293081	0.67111666
C	10.53431109	3.14634790	0.22290950
H	11.37947512	3.79483210	0.45268787
H	10.48963862	2.96571176	-0.85622102
H	10.64287984	2.19435111	0.75257925
C	7.93117789	2.01671467	-0.06792357
H	8.75658114	1.41990663	-0.43739530
C	6.63719951	1.54152856	-0.18665282
H	6.45476186	0.57823776	-0.64856987
C	2.94642661	2.54080522	-0.02849279
C	1.71144672	1.93365218	0.23075120
H	1.69652938	0.89912895	0.54433501
C	0.48174973	2.61741123	0.12380809
C	-0.73243151	1.87158111	0.43761207
O	-0.56485117	0.57455905	0.60190169
B	-1.61699089	-0.35291677	1.09982244
F	-1.37152547	-0.64465538	2.42982355
F	-1.61483150	-1.48231333	0.30931491
O	-2.97184594	0.28490040	1.00102085
C	-3.11805219	1.57242684	0.83403537
C	-4.52369572	2.07170908	0.91643417
H	-5.14229996	1.55037753	0.17750181
H	-4.57946506	3.14656168	0.74178293
H	-4.93933282	1.84134239	1.90389442
C	-2.02978592	2.41203688	0.58090104
H	-2.19536932	3.46965944	0.46567104
C	0.51593437	3.98970978	-0.28924149
O	-0.65664420	4.64560127	-0.41508687
C	-0.66329890	6.02490405	-0.80571402
H	-0.10482652	6.63716439	-0.09099324
H	-1.71197895	6.32055618	-0.80458369
H	-0.24546570	6.14881394	-1.80950195
C	1.75001800	4.59425662	-0.57790989
H	1.78161531	5.62095414	-0.91849933
C	2.93690802	3.88783367	-0.46273607
H	3.86262452	4.38034256	-0.73759903

Supplementary Table 55 Optimized S_0 geometry of compound **3** at the B3LYP/G/6-31G(d, p) level of theory in Cartesian coordinates (Å). (PCM: dichloromethane)

Atom	X	Y	Z
O	0.67901575	2.34617648	4.32334216
C	0.53406150	1.76275981	3.24789342
C	-0.63570230	0.85086523	3.06280832
C	-1.24060292	0.61340949	1.82859762
H	-0.81051291	1.03846753	0.92971055
C	-2.39140803	-0.19053535	1.70129411
C	-2.84417788	-0.52704549	0.34032611
O	-3.26362831	-1.74743565	0.16400425
B	-3.83475565	-2.23733227	-1.13863106
F	-3.29647738	-3.47055392	-1.40895384
F	-5.21372552	-2.27304140	-1.02171040
O	-3.47155364	-1.29295480	-2.24115525
C	-3.05400882	-0.08044049	-2.01649350
C	-2.89766023	0.77387554	-3.23132526
H	-2.20888829	0.28559822	-3.92923330
H	-2.52194305	1.76605956	-2.98066778
H	-3.86365192	0.86355470	-3.74015846
C	-2.76442395	0.36970059	-0.73100331
H	-2.44543079	1.39039845	-0.57752422
C	-2.97342137	-0.73288272	2.87823998
O	-4.11545595	-1.45211089	2.94067372
C	-5.27419890	-1.07671491	2.16803562
H	-5.21657409	-0.02519610	1.87168459
H	-6.13208297	-1.22062706	2.82788626
H	-5.37130573	-1.70556521	1.28388708
C	-2.34386219	-0.51368872	4.11691569
H	-2.80335573	-0.94592297	4.99947729
C	-1.20702206	0.26664330	4.21126078
H	-0.75059230	0.45392517	5.17730710
C	1.52733899	1.98633555	2.15484862
C	2.25988911	3.18747327	2.16169539
H	2.04679097	3.92772639	2.92558681
C	3.23610958	3.43611290	1.21201143
H	3.77456105	4.37446566	1.23860452
C	3.53564128	2.47879018	0.22541389
O	4.49277187	2.67398967	-0.69949626
C	5.24805221	3.89583420	-0.70046070
H	5.80277579	4.01173905	0.23536684
H	4.59464824	4.75892700	-0.85924662
H	5.94420257	3.80185944	-1.53314260
C	2.82333118	1.24314924	0.20604557
C	3.04510912	0.15819710	-0.76679433
O	2.47289252	-0.97433984	-0.44964660
B	2.41855504	-2.17347246	-1.34472131
F	2.77619241	-3.27932955	-0.60744900
F	1.14519964	-2.27738290	-1.86888930
O	3.38897780	-2.01241867	-2.47409968
C	3.93859001	-0.86746460	-2.75895488
C	4.76653773	-0.85502797	-4.00341417
H	4.14022952	-1.12771922	-4.85987767
H	5.55288009	-1.61345628	-3.92387105
H	5.21768652	0.12209475	-4.17724573
C	3.77871381	0.25913442	-1.95603680
H	4.24964366	1.18483314	-2.23682536
C	1.83336991	1.03290566	1.18469968
H	1.31880697	0.08133377	1.17527708

Supplementary Table 56 Optimized S_1 geometry of compound **3** at the TD-B3LYP/G/6-31G(d, p) level of theory in Cartesian coordinates (Å). (PCM: dichloromethane)

Atom	X	Y	Z
O	-0.04881849	2.03625510	-1.48745498
C	0.07195294	1.42669803	-0.35651511
C	-0.92682300	0.79778001	0.41176701
C	-2.27255667	0.80532962	-0.04997319
H	-2.48267132	1.27500641	-1.00420627
C	-3.30171779	0.17704427	0.65022128
C	-4.63771199	0.15973428	0.03446992
O	-5.35678306	-0.91034811	0.22409735
B	-6.79953076	-0.98636736	-0.17463938
F	-7.04527428	-2.24074038	-0.68106969
F	-7.56903071	-0.69861624	0.94017863
O	-7.09550404	0.02726289	-1.23517262
C	-6.34222556	1.08036969	-1.40170286
C	-6.88288204	2.10431412	-2.34586915
H	-7.06267443	1.63964260	-3.32137713
H	-6.19894224	2.94530246	-2.46321856
H	-7.84927439	2.46570820	-1.97743686
C	-5.12385842	1.21845964	-0.74861436
H	-4.54248969	2.11915537	-0.87971144
C	-3.00653476	-0.47334488	1.88729335
O	-3.94770836	-1.12100128	2.65288232
C	-4.91593765	-0.29059099	3.31066954
H	-4.42431161	0.52297461	3.85653618
H	-5.44125267	-0.93659769	4.01723242
H	-5.64416601	0.12141599	2.60522834
C	-1.68226916	-0.47265911	2.35372360
H	-1.48214742	-0.96995716	3.29795315
C	-0.65651330	0.12971132	1.64920865
H	0.35681465	0.09672802	2.03659806
C	1.52753533	1.53252885	-0.05615023
C	2.04956871	2.71109397	0.53292145
H	1.37472840	3.51573897	0.80397365
C	3.39458951	2.81344186	0.81318603
H	3.77315408	3.69127357	1.31988969
C	4.28150843	1.77054950	0.44975661
O	5.57812759	1.80164809	0.72945303
C	6.16113867	2.92737316	1.42211838
H	5.70589664	3.03950845	2.40884687
H	6.03808104	3.83958379	0.83392865
H	7.21653196	2.68070693	1.51949631
C	3.78630470	0.60690184	-0.23273311
C	4.63965100	-0.51859304	-0.67622149
O	3.99442735	-1.64117427	-0.83372910
B	4.65782208	-2.93893164	-1.19913621
F	4.69528922	-3.73966279	-0.07918686
F	3.96478037	-3.50736927	-2.23969608
O	6.06570188	-2.68004692	-1.65243041
C	6.65506312	-1.53271951	-1.51332630
C	8.07606473	-1.48620349	-1.96871745
H	8.12622001	-1.77609593	-3.02386591
H	8.66122173	-2.22034783	-1.40426656
H	8.50945035	-0.49448316	-1.83993384
C	5.99547446	-0.41837973	-0.98769790
H	6.52305709	0.51513455	-0.88580452
C	2.42210832	0.52731586	-0.48636597
H	2.03349748	-0.34378524	-0.99954089

Supplementary Table 57 Optimized T_1 geometry of compound **3** at the TD-B3LYP/G/6-31G(d, p) level of theory in Cartesian coordinates (Å). (PCM: dichloromethane)

Atom	X	Y	Z
O	-0.41959319	3.23345699	-0.91143024
C	0.03750102	2.09000362	-0.94000558
C	-0.87273844	0.91098336	-0.87648158
C	-2.15556246	1.10518607	-0.35740624
H	-2.42416961	2.09940817	-0.01813022
C	-3.11390072	0.08202863	-0.32343519
C	-4.48663271	0.45332149	0.06693147
O	-5.43845552	-0.04683049	-0.66638408
B	-6.89883543	0.20605474	-0.41186030
F	-7.47081133	0.63064837	-1.58670857
F	-7.46077573	-0.96398076	0.06342904
O	-7.06567538	1.27393214	0.62473959
C	-6.07928757	1.73509343	1.33760953
C	-6.46441887	2.71098206	2.40004378
H	-6.99191065	3.55481397	1.94238015
H	-5.59455193	3.07538688	2.94679005
H	-7.16259664	2.23247353	3.09565338
C	-4.76266941	1.33962780	1.11200142
H	-3.96731456	1.72471183	1.73362558
C	-2.74794380	-1.20587195	-0.80546208
O	-3.52293471	-2.31171864	-0.78245864
C	-4.38566950	-2.60096318	0.33576291
H	-4.03251354	-2.09473171	1.23909738
H	-4.32980942	-3.68127625	0.48189443
H	-5.41267134	-2.30623531	0.12017302
C	-1.47170633	-1.38798032	-1.36271222
H	-1.22164972	-2.37356970	-1.74045746
C	-0.54949099	-0.35394755	-1.40376819
H	0.42244923	-0.53024958	-1.85086789
C	1.52100174	1.91120382	-1.03829159
C	2.26313070	2.91346004	-1.72156979
H	1.73504252	3.73285246	-2.19502864
C	3.64505396	2.82446687	-1.80260238
H	4.19294459	3.56613465	-2.36977736
C	4.33165807	1.77441823	-1.18172045
O	5.65896507	1.59854162	-1.30039322
C	6.44413462	2.54059957	-2.04856200
H	6.36968610	3.54109066	-1.61259152
H	6.12377036	2.56144187	-3.09441084
H	7.46934964	2.17915081	-1.98086756
C	3.61305435	0.78648042	-0.38661407
C	4.20638437	-0.26540587	0.36562536
O	3.38162029	-1.20695223	0.85970232
B	3.75294455	-2.04168599	2.01061457
F	3.43736986	-1.41116337	3.20464914
F	3.14375499	-3.27054741	1.88659649
O	5.25710872	-2.29229003	2.03110767
C	6.08919321	-1.44313501	1.48520325
C	7.54254215	-1.66851372	1.73875069
H	7.83359122	-2.66648423	1.38980300
H	7.74653655	-1.63238195	2.81577614
H	8.15627970	-0.92163223	1.23280431
C	5.59991416	-0.37813554	0.69206941
H	6.29733321	0.36237159	0.33054322
C	2.18759173	0.88431668	-0.39320448
H	1.64053481	0.14471662	0.17569108

Supplementary Table 58 Optimized T_2 geometry of compound **3** at the TD-B3LYP/G/6-31G(d, p) level of theory in Cartesian coordinates (Å). (PCM: dichloromethane)

Atom	X	Y	Z
O	0.72190886	2.18425263	4.18017327
C	0.55070562	1.62591412	3.09668859
C	-0.58040726	0.66202734	2.93728047
C	-1.26273304	0.51542890	1.73669446
H	-0.90212708	1.04765069	0.86600989
C	-2.40176395	-0.32383809	1.60681350
C	-3.01528787	-0.48010175	0.30338638
O	-3.74052550	-1.55640290	0.07141362
B	-4.64080891	-1.67932398	-1.10650517
F	-4.61302349	-2.97832639	-1.54984392
F	-5.91281747	-1.26349433	-0.74484799
O	-4.16744700	-0.79217445	-2.22975157
C	-3.41634248	0.25229599	-2.00529168
C	-3.18890970	1.15623389	-3.17058839
H	-2.73735570	0.58841261	-3.99197540
H	-2.53871133	1.99234197	-2.91056158
H	-4.14894776	1.54026617	-3.53388751
C	-2.85170615	0.47293173	-0.73835695
H	-2.28600378	1.38019271	-0.58088630
C	-2.83353351	-1.03111887	2.78403158
O	-3.89849227	-1.87457040	2.81969301
C	-5.21858334	-1.31068167	2.66972728
H	-5.34335807	-0.46346748	3.35179398
H	-5.90787854	-2.10858649	2.94934432
H	-5.41613699	-1.00852841	1.64051682
C	-2.12333643	-0.89017133	3.98112055
H	-2.48033882	-1.44282570	4.84364886
C	-1.02760192	-0.04537786	4.07707464
H	-0.51032603	0.08732295	5.02061360
C	1.46919609	1.92582004	1.95805109
C	2.09203813	3.19450893	1.92016801
H	1.83899923	3.92910006	2.67643434
C	2.99645858	3.50775151	0.91886784
H	3.42695093	4.50002862	0.88198342
C	3.35032845	2.55264370	-0.04748565
O	4.18036043	2.82562359	-1.06850524
C	4.75901005	4.13483902	-1.19155918
H	5.38078177	4.36896630	-0.32258670
H	3.97807249	4.89161478	-1.31015043
H	5.37487551	4.09326063	-2.08897258
C	2.79423040	1.22576380	0.01260970
C	3.16808604	0.12842446	-0.86491230
O	2.37389652	-0.92615063	-0.82438520
B	2.56089893	-2.15795438	-1.63917554
F	2.57353136	-3.25054124	-0.79678239
F	1.55056442	-2.23574268	-2.57819768
O	3.87351098	-2.12378296	-2.37231943
C	4.65269702	-1.07643928	-2.38147923
C	5.89979032	-1.20923039	-3.19255153
H	5.64113606	-1.43616349	-4.23286881
H	6.49451125	-2.05015947	-2.81810129
H	6.49968076	-0.29918625	-3.15853354
C	4.33336082	0.08200019	-1.66270798
H	5.00771687	0.92194604	-1.69170876
C	1.82334356	0.97158696	1.00954079
H	1.39401494	-0.02113344	1.04766591

Supplementary Table 59 Optimized S_0 geometry of compound **1** at the ωB97XD/6-31G(d, p) level of theory in Cartesian coordinates (Å).

Atom	X	Y	Z
C	0.06311800	3.22247400	-0.29864800
C	-1.26872400	2.91985400	-0.06690900
C	-1.68795200	1.60162700	0.13550700
C	-0.72258000	0.59487700	0.14614500
C	0.62948200	0.87060800	-0.05468800
C	1.02928000	2.20625300	-0.30177700
C	-3.13338100	1.34597400	0.41480200
C	-3.71524600	-0.00689600	0.13097700
C	-4.76717600	-0.45249200	0.93800000
C	-5.36443700	-1.68313600	0.69495500
C	-4.93474500	-2.46822300	-0.37449600
C	-3.90782300	-2.01850900	-1.20073400
C	-3.29471500	-0.79496800	-0.94631200
O	-3.83677100	2.23244500	0.87147300
C	1.60405300	-0.23848100	0.01033100
C	1.28661000	-1.51219300	-0.47733600
C	2.16925600	-2.55740400	-0.22792700
O	3.30808000	-2.37365500	0.34038200
O	2.72787400	0.00238300	0.57924900
C	1.85514400	-3.97791000	-0.57558500
B	3.88230300	-0.98456900	0.56168600
F	4.48438900	-0.95620900	1.77833300
F	4.68842000	-0.68202300	-0.49959500
O	2.32505800	2.41291500	-0.56282900
C	2.78397600	3.73113000	-0.80208700
H	0.35101200	4.25071500	-0.47716200
H	-2.01323000	3.70861200	-0.04271300
H	-1.01634400	-0.42565200	0.36722100
H	-5.10288500	0.18142800	1.75168300
H	-6.17021200	-2.02914900	1.33401200
H	-5.40656700	-3.42629200	-0.56822700
H	-3.58796800	-2.61744500	-2.04734700
H	-2.50831600	-0.43867300	-1.60444800
H	0.35035700	-1.69544100	-0.98401500
H	0.86430100	-4.08064000	-1.01858600
H	2.61012600	-4.35171100	-1.27290000
H	1.91740500	-4.58929500	0.32887600
H	3.85601200	3.63978400	-0.96822500
H	2.31112700	4.16353600	-1.69139100
H	2.60359000	4.37785300	0.06360500

Supplementary Table 60 Optimized S_1 geometry of compound **1** at the TD-ωB97XD/6-31G(d, p) level of theory in Cartesian coordinates (Å).

Atom	X	Y	Z
C	0.06430230	3.19485944	-0.31384724
C	-1.26838798	2.88023031	-0.09032302
C	-1.67587265	1.55075913	0.12546290
C	-0.68145611	0.55721963	0.15738590
C	0.65645678	0.85507199	-0.06793240
C	1.04245858	2.19582746	-0.32221548
C	-3.07301213	1.23964308	0.38298904
C	-3.74843466	-0.01110425	0.12784341
C	-4.95080131	-0.30671452	0.80856140
C	-5.61948230	-1.49876945	0.56672404
C	-5.11735725	-2.42089518	-0.34907628
C	-3.93992316	-2.12902696	-1.04205133
C	-3.26484855	-0.93949869	-0.82203605
O	-3.76781974	2.21288555	0.92160805
C	1.64838185	-0.24063447	0.00241092
C	1.35654683	-1.51353430	-0.50027252
C	2.25246815	-2.54758720	-0.24908839
O	3.37977469	-2.35104677	0.33677865
O	2.75882178	0.01403277	0.59061367
C	1.96641128	-3.96905468	-0.61606784
B	3.92812891	-0.95563544	0.58667974
F	4.50301614	-0.93351128	1.81654677
F	4.75225149	-0.62937290	-0.45305445
O	2.33742648	2.41235921	-0.59613166
C	2.77938611	3.73608005	-0.82744009
H	0.33513687	4.22667227	-0.49923832
H	-2.00971914	3.67110891	-0.10898531
H	-0.95269733	-0.45993173	0.41615697
H	-5.33650081	0.39400048	1.54070567
H	-6.53812187	-1.71237196	1.10390231
H	-5.64100139	-3.35329139	-0.53078211
H	-3.55676991	-2.82894861	-1.77821509
H	-2.38555395	-0.70287498	-1.41104157
H	0.42929805	-1.70459031	-1.02037367
H	0.98216620	-4.08350552	-1.07068185
H	2.73476473	-4.32136133	-1.31001778
H	2.03027229	-4.58992034	0.28175073
H	3.85203122	3.66047269	-0.99824719
H	2.29807219	4.17005570	-1.71197813
H	2.59392927	4.37554659	0.04310425

Supplementary Table 61 Optimized T_1 geometry of compound **1** at the TD-ωB97XD/6-31G(d, p) level of theory in Cartesian coordinates (Å).

Atom	X	Y	Z
C	-0.10724400	3.24726100	0.14877700
C	1.25010000	2.96215300	0.02918400
C	1.67667300	1.62620600	-0.14424500
C	0.74027700	0.61695800	-0.24466800
C	-0.66596000	0.85574600	-0.14534800
C	-1.06452900	2.24135200	0.07943100
C	3.13805400	1.36663800	-0.32097600
C	3.70078600	0.01246500	-0.00665700
C	4.80551900	-0.42406600	-0.74305700
C	5.38625998	-1.65586300	-0.47177100
C	4.88501800	-2.45152000	0.55664600
C	3.80330800	-2.01113400	1.31388500
C	3.20817500	-0.78579900	1.03085200
O	3.86862900	2.25337200	-0.72942900
C	-1.59797100	-0.20941800	-0.27414500
C	-1.17736800	-1.58069000	-0.18291800
C	-2.10818800	-2.64190100	-0.27617800
O	-3.36496900	-2.39649900	-0.33553500
O	-2.88442100	0.01221200	-0.48190100
C	-1.69296100	-4.07675400	-0.32912300
B	-3.90025200	-0.94408700	-0.03004700
F	-5.03515900	-0.79975300	-0.75554700
F	-4.05600900	-0.91349400	1.33137800
O	-2.37578200	2.44925000	0.23502000
C	-2.84207000	3.75994000	0.48304600
H	-0.41509600	4.27282200	0.30895800
H	1.99274800	3.74957800	0.06905200
H	1.09291700	-0.38195200	-0.46247500
H	5.19596400	0.21863200	-1.52483100
H	6.23530698	-1.99433600	-1.05653500
H	5.34428803	-3.41072100	0.77364900
H	3.42713100	-2.61814100	2.13108300
H	2.37637300	-0.43633200	1.63421600
H	-0.13737000	-1.83914400	-0.03781400
H	-0.60782500	-4.18988800	-0.31831800
H	-2.11958900	-4.61285400	0.52408700
H	-2.09221200	-4.53959500	-1.23626600
H	-3.92304500	3.67230600	0.57822200
H	-2.42213000	4.16224500	1.41230500
H	-2.60060800	4.43091900	-0.34923700

Supplementary Table 62 Optimized T_2 geometry of compound **1** at the TD-ωB97XD/6-31G(d, p) level of theory in Cartesian coordinates (Å).

Atom	X	Y	Z
C	0.00252794	3.21565508	-0.01325503
C	1.33827692	2.86626334	-0.12983765
C	1.72939427	1.52247587	-0.22428087
C	0.72897458	0.54428679	-0.23616230
C	-0.62549097	0.86565975	-0.11611808
C	-0.99444891	2.23225889	-0.00002651
C	3.15546539	1.19158657	-0.36848737
C	3.74579702	-0.08219366	0.05297090
C	4.92977531	-0.52675092	-0.57983373
C	5.52830220	-1.71545600	-0.19055517
C	4.98064150	-2.47607981	0.84539543
C	3.82735159	-2.02787016	1.50124380
C	3.22085247	-0.84021800	1.12291184
O	3.93296906	2.05910540	-0.86105676
C	-1.62623157	-0.20675422	-0.13080151
C	-1.45446118	-1.36906242	-0.91973685
C	-2.37920562	-2.42468222	-0.84655269
O	-3.43374252	-2.33001756	-0.10418155
O	-2.67602945	-0.08832893	0.62123664
C	-2.21091572	-3.71037236	-1.58829761
B	-3.87127961	-1.00890009	0.52694752
F	-4.79434181	-0.44166788	-0.30651298
F	-4.30252557	-1.27433476	1.78734732
O	-2.30489117	2.50201373	0.07130881
C	-2.73110068	3.84901012	0.14737277
H	-0.26299907	4.26236514	0.06475712
H	2.10118345	3.63667259	-0.13834666
H	1.01342135	-0.49895849	-0.32462422
H	5.34561039	0.06804049	-1.38485784
H	6.42610500	-2.05547322	-0.69663901
H	5.45200432	-3.40493747	1.14886635
H	3.41123190	-2.60445000	2.32110780
H	2.35058360	-0.48099583	1.66206077
H	-0.60378326	-1.45376038	-1.58298185
H	-1.27704524	-3.73575752	-2.15167184
H	-2.22862924	-4.54622014	-0.88235614
H	-3.05034800	-3.85022965	-2.27670058
H	-3.81815187	3.80821816	0.19346367
H	-2.34704493	4.33809528	1.04981094
H	-2.42340823	4.41719439	-0.73828838

Supplementary Table 63 Optimized S_0 geometry of compound **2** at the ωB97XD/6-31G(d, p) level of theory in Cartesian coordinates (Å).

Atom	X	Y	Z
O	4.01067700	0.50178700	-0.54353600
C	4.13756200	1.68045800	-0.25479400
C	5.48649700	2.26028200	0.00632300
C	5.70077200	3.36976300	0.83781800
H	4.85993100	3.86025400	1.31753900
C	6.98120400	3.82955800	1.08822000
H	7.15973900	4.67636800	1.74119700
C	8.08286700	3.19215000	0.50339100
O	9.29051800	3.72294700	0.79853200
C	10.44310900	3.10643800	0.26064900
H	11.29139400	3.68269800	0.62892900
H	10.43821900	3.13164300	-0.83572100
H	10.53775500	2.06765500	0.59870400
C	7.88585700	2.07769200	-0.31982300
H	8.72251100	1.56299500	-0.77619600
C	6.59553400	1.61841600	-0.54837900
H	6.42885300	0.74311000	-1.16718100
C	2.92191400	2.55494900	-0.17091400
C	1.71237900	1.96920700	0.19704700
H	1.69624400	0.91676600	0.45551800
C	0.52058400	2.69329900	0.23801100
C	-0.70033400	1.96622500	0.65554900
O	-0.47139400	0.89625400	1.33447400
B	-1.55756700	0.04894400	1.97115300
F	-1.65211200	0.39825300	3.28832100
F	-1.26940700	-1.25802400	1.73677100
O	-2.87209300	0.38179400	1.29540100
C	-3.04066200	1.45446000	0.60779500
C	-4.44299100	1.68778500	0.14139700
H	-4.78098900	0.81872100	-0.42948200
H	-4.52460400	2.58472700	-0.47270200
H	-5.09818000	1.77908200	1.01239300
C	-2.00194700	2.32446800	0.29512000
H	-2.18864700	3.21134200	-0.28588200
C	0.54976200	4.06392300	-0.11554300
O	-0.60777900	4.75294200	-0.04053100
C	-0.60115700	6.14220500	-0.31207900
H	0.06809400	6.67715800	0.37060800
H	-1.62450500	6.47916700	-0.15136900
H	-0.31034500	6.34686400	-1.34859900
C	1.75662200	4.65198200	-0.50314900
H	1.79247400	5.69500200	-0.78983000
C	2.92618900	3.90382000	-0.52783000
H	3.84755000	4.37857200	-0.84932000

Supplementary Table 64 Optimized S_1 geometry of compound **2** at the TD-ωB97XD/6-31G(d, p) level of theory in Cartesian coordinates (Å).

Atom	X	Y	Z
O	1.62955752	-1.97580480	-0.73971998
C	1.90804461	-0.74290055	-0.39331948
C	3.33034245	-0.49947992	-0.16916395
C	3.78563772	0.39684353	0.82176255
H	3.07036575	0.91721930	1.44920956
C	5.13536259	0.59721680	1.02094431
H	5.49258756	1.28850541	1.77619579
C	6.08107961	-0.11711507	0.26793874
O	7.37650605	0.15435284	0.54899174
C	8.37389995	-0.55877150	-0.15264489
H	9.32805149	-0.19884026	0.23124612
H	8.32277889	-0.36557152	-1.23119811
H	8.29648535	-1.63821400	0.02540212
C	5.64808443	-1.03735541	-0.68812206
H	6.35493606	-1.60705155	-1.27896559
C	4.28618998	-1.22355842	-0.90012935
H	3.95746977	-1.92390047	-1.65960443
C	0.83045257	0.19824295	-0.25707407
C	-0.49922206	-0.25671642	-0.17697430
H	-0.70972836	-1.31893706	-0.17652000
C	-1.58193545	0.61961583	-0.08056934
C	-2.93075322	0.01021922	-0.02085586
O	-2.93634113	-1.23838584	0.29713865
B	-4.19429724	-2.05229226	0.52937391
F	-4.41306992	-2.12590785	1.87596936
F	-4.04605825	-3.24987405	-0.09814494
O	-5.35640618	-1.31743230	-0.10312096
C	-5.29257408	-0.07307530	-0.42122280
C	-6.58125600	0.51846986	-0.89902253
H	-6.94905390	-0.06138347	-1.74996830
H	-6.46737897	1.56312997	-1.18887880
H	-7.32795690	0.43770265	-0.10400539
C	-4.11854601	0.66774590	-0.35461710
H	-4.11218901	1.70716350	-0.63377312
C	-1.33543925	2.00990337	-0.03440688
O	-2.39418853	2.84151434	0.10920061
C	-2.16161107	4.22957315	0.24145865
H	-1.52726159	4.44723646	1.10822330
H	-3.14178032	4.68132483	0.39101822
H	-1.70445115	4.64988412	-0.66195641
C	-0.01547569	2.47229211	-0.12021048
H	0.19241733	3.53476520	-0.10960781
C	1.04285357	1.59303811	-0.24579682
H	2.04451826	1.99138197	-0.36187294

Supplementary Table 65 Optimized T_1 geometry of compound **2** at the TD-ωB97XD/6-31G(d, p) level of theory in Cartesian coordinates (Å).

Atom	X	Y	Z
O	1.74756129	-1.88624623	-1.40028819
C	1.98082783	-0.81899446	-0.85725362
C	3.35507700	-0.49014286	-0.38106883
C	3.61404384	0.42172051	0.65310220
H	2.79388173	0.94342420	1.13553900
C	4.90655639	0.64522275	1.09214940
H	5.11725443	1.33744856	1.89947439
C	5.97715614	-0.03558015	0.49854557
O	7.20210025	0.25975925	0.98836999
C	8.32062339	-0.41239419	0.44552738
H	9.18905622	-0.03309009	0.98341077
H	8.43742894	-0.20011523	-0.62403872
H	8.24884648	-1.49647706	0.59384493
C	5.73392422	-0.95519398	-0.52793561
H	6.54468885	-1.49892274	-0.99720335
C	4.42929269	-1.18008536	-0.94663559
H	4.22300999	-1.90457541	-1.72727416
C	0.86983795	0.17636053	-0.66965289
C	-0.40642125	-0.31293102	-0.45469536
H	-0.56500491	-1.38338057	-0.40457399
C	-1.53230194	0.55042352	-0.32480298
C	-2.81638590	-0.03298172	-0.14108560
O	-2.89919516	-1.34123719	0.06643051
B	-4.09322582	-1.92324470	0.70007580
F	-4.15091249	-1.62164156	2.03517026
F	-4.20260715	-3.23259131	0.37935786
O	-5.36715144	-1.21533192	0.08190148
C	-5.31091742	0.00434065	-0.31377635
C	-6.59919537	0.66359041	-0.68635440
H	-7.08397303	0.09682814	-1.48699166
H	-6.44842670	1.69162930	-1.01894208
H	-7.27741916	0.65759281	0.17236334
C	-4.06207072	0.66817868	-0.38557257
H	-4.02497333	1.71003713	-0.67499696
C	-1.27456941	1.96125579	-0.40294228
O	-2.28647696	2.87388444	-0.20733208
C	-2.47706354	3.21597559	1.16305160
H	-2.73953286	2.33321400	1.75927239
H	-3.29464227	3.93733175	1.19523377
H	-1.57094246	3.67037573	1.57897378
C	-0.00051397	2.43934637	-0.65399174
H	0.13632663	3.51312909	-0.72703663
C	1.07872583	1.56644689	-0.79245562
H	2.06776937	1.95589525	-1.00575679

Supplementary Table 66 Optimized T_2 geometry of compound **2** at the TD-ωB97XD/6-31G(d, p) level of theory in Cartesian coordinates (Å).

Atom	X	Y	Z
O	1.70603770	-1.83542314	-1.10716180
C	1.97666396	-0.61740031	-0.68269180
C	3.34019731	-0.39616144	-0.32980125
C	3.74783445	0.57269320	0.64618669
H	2.99550077	1.16695218	1.15281408
C	5.06878883	0.74055176	0.96082260
H	5.38319382	1.47762756	1.69171960
C	6.06680521	-0.07275274	0.36269525
O	7.33173576	0.18677876	0.75447829
C	8.37448940	-0.61230068	0.22970494
H	9.29362322	-0.24273544	0.68295384
H	8.44281279	-0.51405736	-0.86005116
H	8.23885430	-1.66793154	0.49219234
C	5.69112765	-1.06983535	-0.55267269
H	6.43410526	-1.70991058	-1.01356300
C	4.36189910	-1.23750450	-0.88502481
H	4.06949545	-1.99713237	-1.59878451
C	0.86501543	0.30418696	-0.52061693
C	-0.43880034	-0.19816903	-0.36221546
H	-0.59269648	-1.26957156	-0.32618141
C	-1.54367180	0.64742260	-0.24533138
C	-2.87604721	0.01707378	-0.10720630
O	-2.86193104	-1.19071666	0.33840454
B	-4.12390200	-1.94867101	0.70721107
F	-4.34834519	-1.77076160	2.04436431
F	-3.98051336	-3.23833030	0.30805388
O	-5.28060853	-1.33225204	-0.05304862
C	-5.23367710	-0.12934026	-0.50469614
C	-6.52018197	0.38427904	-1.06855419
H	-6.85919152	-0.28982076	-1.85998244
H	-6.41589924	1.39355901	-1.46686001
H	-7.28338791	0.37453701	-0.28524734
C	-4.06868956	0.63055136	-0.50080817
H	-4.06016325	1.63493217	-0.89295484
C	-1.33978300	2.04264658	-0.27292882
O	-2.36993198	2.93730128	-0.14675676
C	-2.74953184	3.19954780	1.20050072
H	-3.12937405	2.29557386	1.69082627
H	-3.54145932	3.94798423	1.15926836
H	-1.90131851	3.59327518	1.77250538
C	-0.05168173	2.55077501	-0.44864733
H	0.06990862	3.62776402	-0.49153901
C	1.03204264	1.70351265	-0.59498902
H	2.01602145	2.11997228	-0.78461590

Supplementary Table 67 Optimized S_0 geometry of compound **3** at the ωB97XD/6-31G(d, p) level of theory in Cartesian coordinates (Å).

Atom	X	Y	Z
O	-0.45579648	3.42131630	-0.08721002
C	0.06759786	2.37113072	0.25679609
C	-0.77683476	1.23187576	0.72353999
C	-2.09656824	1.19279638	0.28369757
H	-2.41979685	1.94974238	-0.42301000
C	-2.98650161	0.18620517	0.67327177
C	-4.29172821	0.14136951	-0.01596085
O	-4.67240683	-1.01044052	-0.44493306
B	-6.04252376	-1.24143521	-1.06542419
F	-5.89767933	-2.06409563	-2.13302616
F	-6.87001811	-1.71216763	-0.07409554
O	-6.58306293	0.08920311	-1.54056277
C	-6.15621563	1.21263902	-1.07930799
C	-6.93938282	2.41224255	-1.50678608
H	-6.95852866	2.45506446	-2.59932263
H	-6.51781490	3.33669780	-1.11210735
H	-7.97375699	2.30494916	-1.16823028
C	-5.03579288	1.29999388	-0.25975996
H	-4.71205278	2.25357949	0.13120222
C	-2.54558547	-0.78537203	1.60464015
O	-3.28822062	-1.75693813	2.15427598
C	-4.68322569	-1.58455070	2.40183023
H	-4.93216897	-0.52321167	2.51213989
H	-4.88280818	-2.09773650	3.34334939
H	-5.28322550	-2.01894978	1.60400611
C	-1.21510677	-0.74409433	2.04812784
H	-0.90203785	-1.49328258	2.76554534
C	-0.34386116	0.23599290	1.61356525
H	0.66735487	0.24807783	2.00554031
C	1.55776698	2.24044972	0.20690890
C	2.32205922	3.40987826	0.27673161
H	1.81006593	4.35894549	0.39356187
C	3.70360720	3.36589860	0.19981961
H	4.26660388	4.28689447	0.27868616
C	4.36473317	2.14317016	0.02040979
O	5.70727544	2.04190338	-0.03847691
C	6.49808257	3.20731383	0.12669712
H	6.31935615	3.67282570	1.10176831
H	6.31003644	3.93440714	-0.67074740
H	7.53231223	2.87031874	0.07204740
C	3.61108455	0.95092025	-0.08487780
C	4.19216126	-0.39723008	-0.27389235
O	3.40012935	-1.35942019	0.05995336
B	3.79646448	-2.82501311	0.07283112
F	4.07817274	-3.17598917	1.36306152
F	2.80045063	-3.53748281	-0.51770530
O	5.05814539	-2.98022633	-0.75148890
C	5.79965925	-1.98292544	-1.08043149
C	7.06840241	-2.34810715	-1.78464740
H	6.82817099	-2.93182695	-2.67754787
H	7.66879555	-2.98739120	-1.13120583
H	7.64632034	-1.46801649	-2.06686375
C	5.44758426	-0.66005761	-0.82645752
H	6.09992710	0.14419982	-1.12036012
C	2.21757558	1.02961555	0.01775487
H	1.65963734	0.10646091	-0.08207459

Supplementary Table 68 Optimized S_1 geometry of compound **3** at the TD-ωB97XD/6-31G(d, p) level of theory in Cartesian coordinates (Å).

Atom	X	Y	Z
O	-0.54914251	3.33530233	0.37771901
C	0.08807541	2.19186201	0.48929552
C	-0.78255228	1.06640330	0.80030676
C	-2.11513340	1.09390617	0.36741065
H	-2.44233349	1.90559806	-0.27235096
C	-3.01353223	0.05598461	0.65147155
C	-4.33145718	0.11649678	-0.01188268
O	-4.73604908	-0.96467992	-0.57971189
B	-6.11864907	-1.09736304	-1.20151053
F	-5.99989663	-1.76740747	-2.37388315
F	-6.93506124	-1.69152937	-0.26956262
O	-6.65261857	0.29046396	-1.48164448
C	-6.20247185	1.33850369	-0.88536202
C	-6.98031409	2.59131236	-1.13178278
H	-7.02191340	2.77981519	-2.20816434
H	-6.54016126	3.45114048	-0.62668891
H	-8.00857040	2.44884099	-0.78764015
C	-5.06377160	1.30561663	-0.08716112
H	-4.72055995	2.19502235	0.42097049
C	-2.57429106	-1.03258475	1.43607279
O	-3.31582026	-2.06454792	1.86668544
C	-4.70180219	-1.91392467	2.17022065
H	-4.93014170	-0.87781012	2.44451170
H	-4.88949890	-2.56047704	3.02837093
H	-5.32662615	-2.21588804	1.33116979
C	-1.23340429	-1.06092753	1.86377386
H	-0.91927478	-1.90278073	2.46974957
C	-0.35923735	-0.03806467	1.57421940
H	0.64742881	-0.07453724	1.97444492
C	1.51480794	2.18380123	0.32401018
C	2.24192922	3.39466956	0.33796100
H	1.72351735	4.33320822	0.49844509
C	3.61762641	3.40259574	0.17038064
H	4.13902289	4.35098453	0.20030697
C	4.32729952	2.21324130	-0.01616410
O	5.67383758	2.16652705	-0.14191142
C	6.41190136	3.36941780	-0.03550979
H	6.25734473	3.84640780	0.93886397
H	6.15079844	4.07306018	-0.83439933
H	7.45862732	3.08431814	-0.13470631
C	3.61827120	0.98520609	-0.05440467
C	4.24845986	-0.34047942	-0.25449200
O	3.52777378	-1.33115640	0.14934881
B	3.96884151	-2.78297231	0.12814793
F	4.28095759	-3.14924217	1.40682375
F	2.98167839	-3.51526645	-0.45694511
O	5.21842916	-2.88976043	-0.71996154
C	5.88854456	-1.86471788	-1.11253411
C	7.14309805	-2.18510317	-1.86269424
H	6.89530527	-2.80430326	-2.72926310
H	7.80308147	-2.77515108	-1.22051814
H	7.66222164	-1.28496985	-2.19202165
C	5.47878074	-0.55548525	-0.87949409
H	6.07156077	0.27352327	-1.22630888
C	2.23572215	0.99583348	0.10416240
H	1.71953254	0.04811397	0.02313134

Supplementary Table 69 Optimized T_1 geometry of compound **3** at the TD-ωB97XD/6-31G(d, p) level of theory in Cartesian coordinates (Å).

Atom	X	Y	Z
O	0.44174505	3.35627519	-0.05876909
C	-0.08011746	2.28480608	-0.33352620
C	0.76933027	1.09417915	-0.65082051
C	2.05764261	1.09620686	-0.14403462
H	2.32617661	1.95283735	0.46008771
C	2.98975416	0.03549439	-0.38116942
C	4.24687764	0.01201040	0.27631982
O	5.05167375	-1.03985916	0.20889849
B	6.50834258	-0.86742448	0.27717861
F	7.10898983	-2.02567555	0.63615784
F	6.98742057	-0.27739979	-0.87180962
O	6.82319037	0.16923536	1.42098845
C	5.99278877	1.11068050	1.68605244
C	6.43893977	2.15963454	2.65304636
H	6.70373999	1.69055324	3.60541209
H	5.66704871	2.91043113	2.82773865
H	7.33922727	2.65020883	2.27084147
C	4.71172121	1.12176489	1.08175792
H	4.08461348	1.98367159	1.26039347
C	2.53044316	-1.02205551	-1.26830480
O	3.30704209	-2.07646717	-1.60689119
C	4.35842883	-1.81118316	-2.53489366
H	5.05600686	-1.05766748	-2.16334332
H	3.93662318	-1.49448986	-3.49648067
H	4.89179378	-2.75284684	-2.65897353
C	1.23681067	-1.00439315	-1.76504781
H	0.94216691	-1.82351020	-2.41116695
C	0.34376783	0.02779533	-1.47106787
H	-0.64514894	0.02367761	-1.91366888
C	-1.57199715	2.17702649	-0.35055344
C	-2.31301106	3.34058206	-0.58158419
H	-1.78181498	4.26737557	-0.76974764
C	-3.69716681	3.31808861	-0.57481464
H	-4.24135653	4.23075336	-0.78045430
C	-4.38533337	2.12776726	-0.30330548
O	-5.73054246	2.04777244	-0.30688973
C	-6.49255664	3.19655671	-0.63956987
H	-6.25330843	3.55280975	-1.64714398
H	-6.33700706	4.00312416	0.08512231
H	-7.53343525	2.87760998	-0.60738384
C	-3.65687009	0.94584512	-0.03124791
C	-4.26702784	-0.36748201	0.27663624
O	-3.47092591	-1.36690960	0.10194401
B	-3.84961123	-2.82417437	0.30010633
F	-3.92942215	-3.41101162	-0.92944278
F	-2.94604930	-3.38247344	1.15159460
O	-5.21870545	-2.87879396	0.94689457
C	-5.96272478	-1.84394591	1.11226056
C	-7.30271340	-2.12440309	1.71657563
H	-7.16520290	-2.62412716	2.67940824
H	-7.84987137	-2.81468880	1.06828909
H	-7.88588103	-1.21408031	1.85603794
C	-5.55936163	-0.55606507	0.77129036
H	-6.21721822	0.28113959	0.92900430
C	-2.25886976	0.99915989	-0.07043942
H	-1.71960874	0.08580946	0.15034188

Supplementary Table 70 Optimized T_2 geometry of compound **3** at the TD-ωB97XD/6-31G(d, p) level of theory in Cartesian coordinates (Å).

Atom	X	Y	Z
O	-0.43366046	3.35666860	0.03851709
C	0.08689130	2.28719059	0.32325592
C	-0.76404769	1.10106665	0.65331601
C	-2.05200570	1.09867652	0.14566105
H	-2.31943514	1.94897480	-0.46779070
C	-2.98522068	0.04142277	0.39390148
C	-4.24141368	0.01080505	-0.26489088
O	-5.04831358	-1.03850805	-0.18346593
B	-6.50448662	-0.86388273	-0.25523144
F	-7.10753576	-2.02592905	-0.59753476
F	-6.98303618	-0.25592859	0.88459506
O	-6.81615750	0.15650719	-1.41458178
C	-5.98310790	1.09149896	-1.69361300
C	-6.42538537	2.12595815	-2.67784010
H	-6.68766915	1.64259508	-3.62374939
H	-5.65223154	2.87325826	-2.86168832
H	-7.32637831	2.62307034	-2.30593042
C	-4.70273416	1.10910635	-1.08795320
H	-4.07315739	1.96641207	-1.27959027
C	-2.52784184	-1.00592347	1.29406992
O	-3.30601316	-2.05525410	1.64472358
C	-4.35779534	-1.77733027	2.56851350
H	-5.05293437	-1.02602695	2.18803105
H	-3.93631021	-1.45133309	3.52714208
H	-4.89379415	-2.71619966	2.70207718
C	-1.23444193	-0.98413630	1.79114967
H	-0.94106129	-1.79622236	2.44664993
C	-0.33999164	0.04346854	1.48560356
H	0.64892864	0.04291624	1.92819213
C	1.57859151	2.17691431	0.33918730
C	2.32235495	3.34237394	0.55074089
H	1.79289924	4.27270087	0.72604778
C	3.70669819	3.31846389	0.53951635
H	4.25231314	4.23377132	0.72909836
C	4.39244057	2.12334833	0.28394048
O	5.73752310	2.04055970	0.28258439
C	6.50250179	3.19457401	0.58912624
H	6.27068587	3.56941972	1.59173891
H	6.34233090	3.98803962	-0.14889415
H	7.54300410	2.87450716	0.55532281
C	3.66092917	0.93854290	0.03388963
C	4.26538134	-0.38037242	-0.25555007
O	3.48551465	-1.37859700	-0.00808692
B	3.78548137	-2.80484418	-0.43416670
F	3.44971010	-3.63238186	0.59011320
F	3.13608542	-3.03968463	-1.61372066
O	5.27698689	-2.91639217	-0.67922308
C	6.01021807	-1.88673167	-0.91586391
C	7.41061613	-2.19504268	-1.34276963
H	7.38317776	-2.77771159	-2.26788192
H	7.88805299	-2.81816854	-0.58142425
H	7.99874670	-1.29034644	-1.49722005
C	5.54983035	-0.58150571	-0.76502926
H	6.19685411	0.25331803	-0.97317356
C	2.26319786	0.99394117	0.07479786
H	1.72296078	0.07824603	-0.13373966

Supplementary Table 71 Optimized T_3 geometry of compound **3** at the TD-ωB97XD/6-31G(d, p) level of theory in Cartesian coordinates (Å).

Atom	X	Y	Z
O	-0.49577227	3.33768024	-0.07457490
C	0.07137950	2.26114565	0.24176578
C	-0.77095389	1.13661863	0.65940110
C	-2.10971444	1.11386989	0.22937617
H	-2.42524805	1.87598601	-0.47473721
C	-3.00586472	0.11004554	0.60954348
C	-4.32963558	0.10781533	-0.03155316
O	-4.78447577	-1.02402646	-0.47925511
B	-6.18889052	-1.18783280	-1.03719350
F	-6.11754722	-1.96527376	-2.14526364
F	-6.97997732	-1.67700229	-0.02513129
O	-6.73566885	0.16961382	-1.43729618
C	-6.25866307	1.28307420	-0.97644412
C	-7.03806791	2.51461808	-1.29954728
H	-7.14071062	2.60970365	-2.38496462
H	-6.55990550	3.41222468	-0.90519788
H	-8.04783122	2.43178465	-0.88521368
C	-5.06446514	1.29727294	-0.23461369
H	-4.69387555	2.23040011	0.16969200
C	-2.55627132	-0.89221534	1.50499809
O	-3.28976090	-1.87189324	2.05113361
C	-4.67742860	-1.70590272	2.33781796
H	-4.91435584	-0.65151785	2.51762803
H	-4.85924145	-2.27595065	3.24961398
H	-5.29830364	-2.08625107	1.52838758
C	-1.20859232	-0.87884590	1.92643361
H	-0.89366190	-1.65711727	2.61164910
C	-0.33947884	0.11080053	1.52925620
H	0.67255418	0.11106524	1.91874195
C	1.54533799	2.19704895	0.19817031
C	2.28085822	3.38776644	0.29715697
H	1.74804418	4.32250292	0.43125068
C	3.66401620	3.37854938	0.23829880
H	4.20083261	4.31295646	0.34076158
C	4.36192427	2.17740670	0.05870595
O	5.71044183	2.11152137	0.02628601
C	6.46340527	3.29620287	0.22019733
H	6.25243454	3.74540870	1.19670225
H	6.27021019	4.02786383	-0.57212285
H	7.50827737	2.99071520	0.18242884
C	3.64166328	0.96609264	-0.07599910
C	4.26263645	-0.36036089	-0.27029480
O	3.49245472	-1.35544227	0.02753294
B	3.92262814	-2.81041444	0.01175332
F	4.14065056	-3.20116745	1.30268419
F	2.97701177	-3.52265038	-0.65813244
O	5.23198248	-2.91769263	-0.74514577
C	5.95468309	-1.89415928	-1.04222171
C	7.26455205	-2.21066644	-1.69157750
H	7.08645254	-2.79788899	-2.59689781
H	7.86126381	-2.83012034	-1.01567567
H	7.82163310	-1.30863305	-1.94576254
C	5.54207621	-0.58445878	-0.79558688
H	6.17826962	0.24414392	-1.05737403
C	2.24598523	1.00624164	-0.00877121
H	1.71333212	0.07295799	-0.14563850

3. Supplementary Methods

Materials

4-Methoxybenzophenone (99%, Adamas), 4,4′-dimethoxybenzophenone (98%, Adamas), acetic anhydride (98.5%, Sinopharm Chemical Reagent), boron trifluoride diethyl etherate (98%, TCI), phenyl benzoate (99%, Energy-Chemical), rhodamine 6G (Biological stain, Aladdin), quinine sulfate dihydrate (Biological stain, Macklin) were used as received.

Physical measurements and instrumentation

Nuclear magnetic resonance (NMR) spectra were recorded on a JEOL Fourier-transform NMR spectrometer (400 MHz), including ^{1}H NMR, ^{13}C NMR, ^{19}F NMR, ^{11}B NMR. Mass spectra were performed on Agilent Technologies 5973N and Thermo Fisher Scientific LTQ FT Ultras mass spectrometer. FT-IR spectra were recorded on a Nicolet AVATAR-360 FT-IR spectrophotomerter with a resolution of 4 cm^{-1}. Single-crystal X-ray diffraction analysis was performed on a D8 VENTURE SC-XRD instrument. UV-Vis absorption spectra were recorded on a Techcomp UV1050 UV-vis spectrophotometer. Emission spectra were recorded using Hitachi FL-4700 fluorescence spectrometer, Hitachi FL-7000 fluorescence spectrometer and Horiba FluoroLog-3 fluorescence spectrometer. Photoluminescence quantum yield was measured by a Hamamatsu absolute PL quantum yield measurement system based on a standard protocol. Photographs and videos were captured by Xiaomi 11 Ultra camera. Before the capture, samples were irradiated by a 365 nm UV lamp (5 W) for approximately 5 s at a distance of approximately 15 cm. All animal procedures were reviewed and approved by the Institutional Animal Care and Use Committee at Chinese Academy of Sciences and are in accordance with the Guide for the Care and Use of Laboratory Animals of Chinese Academy of Sciences.

Synthesis of compound 1 via cascade reaction

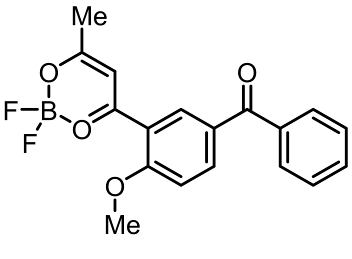

compound 1

In a round bottom flask, boron trifluoride diethyl etherate (2.0 mL, 15.8 mmol) was slowly added into a stirred solution of 4-methoxybenzophenone (425 mg, 2.00 mmol) in acetic anhydride (5.00 mL, 51.4 mmol). The reaction mixture was kept at 80 °C and stirred for 12 h. Then the reaction was quenched by dropwisely adding the reaction mixture into cold water. The precipitates were washed by deionized water for three times and dried under vacuum. The crude product was obtained by column chromatography over silica gel using petroleum ether/dichloromethane (1:2) as eluent. The product was further purified by three times recrystallization in spectroscopic grade dichloromethane/hexane, giving a pale yellow solids with an isolation yield of 22.6% (156 mg). ^{1}H NMR (400 MHz, Chloroform-*d*) δ 8.56 (d, J = 2.3 Hz, 1H), 8.13 (dd, J = 8.7, 2.3 Hz, 1H), 7.82

– 7.71 (m, 2H), 7.60 (t, *J* = 7.4 Hz, 1H), 7.49 (t, *J* = 7.6 Hz, 2H), 7.14 (d, *J* = 8.7 Hz, 1H), 7.00 (s, 1H), 4.07 (s, 3H), 2.40 (s, 3H). ^{13}C NMR (101 MHz, Chloroform-*d*) δ 194.40, 193.37, 179.79, 163.34, 138.01, 137.13, 134.37, 132.89, 130.87, 129.89, 128.66, 120.09, 112.18, 102.68, 56.60, 25.08. ^{19}F NMR (376 MHz, Chloroform-*d*) δ -138.47 (20.9%), -138.53 (79.1%). ^{11}B NMR (128 MHz, Chloroform-*d*) δ -0.10. FT-IR (KBr, cm^{-1}): 3167.0, 3079.4, 2954.0, 2845.2, 1651.9, 1604.1, 1536.8, 1467.2, 1437.1, 1367.7, 1340.9, 1307.5, 1269.1, 1254.8, 1168.7, 1100.7, 1048.4, 1010.7, 978.5, 943.5, 876.5, 833.9, 798.8, 736.6, 708.9, 653.0, 632.6, 605.5, 567.2, 513.3, 471.0, 438.8. LRMS, m/z 345.1. HRMS (ESI) m/z found (calcd for $C_{18}H_{16}O_4{}^{10}BF_2$): 344.1137 (344.1141).

Synthesis of compound 2 and compound 3 via cascade reaction

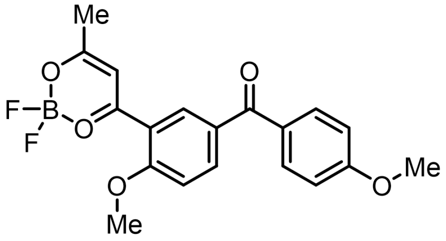

compound 2

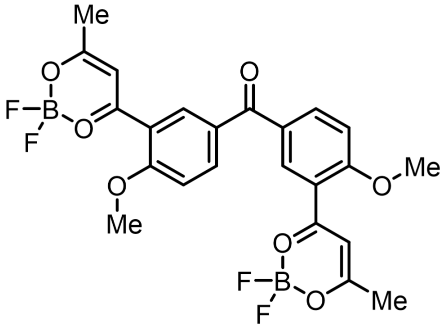

compound 3

In a round bottom flask, boron trifluoride diethyl etherate (2.0 mL, 15.8 mmol) was slowly added into a stirred solution of 4,4′-dimethoxybenzophenone (484 mg, 2.00 mmol) in acetic anhydride (5.00 mL, 51.4 mmol). The reaction mixture was kept at 85 °C and stirred for 16 h. Then the reaction was quenched by dropwisely adding the reaction mixture into cold water. The precipitates were washed by deionized water for three times and dried under vacuum. Compound 2 and 3 were obtained by column chromatography over silica gel using dichloromethane as eluent. The two products were further purified by three times recrystallization in spectroscopic grade dichloromethane/hexane respectively, giving one pale yellow solids (118.2 mg compound 2, 15.8 % yield) and another yellow solids (26.2 mg compound 3, 2.59% yield).

Compound 2: ^{1}H NMR (400 MHz, Chloroform-*d*) δ 8.51 (d, *J* = 2.3 Hz, 1H), 8.09 (dd, *J* = 8.7, 2.3 Hz, 1H), 7.77 (d, *J* = 8.8 Hz, 2H), 7.13 (d, *J* = 8.8 Hz, 1H), 7.01 (s, 1H), 6.97 (d, *J* = 8.8 Hz, 2H), 4.06 (s, 3H), 3.89 (s, 3H), 2.40 (s, 3H). ^{13}C NMR (101 MHz, Chloroform-*d*) δ 193.28, 193.18, 179.82, 163.57, 163.10, 137.87, 133.91, 132.39, 131.47, 129.67, 119.86, 113.97, 112.18, 102.68, 56.57, 55.63, 25.05. ^{19}F NMR (376 MHz, Chloroform-*d*) δ -138.42 (20.2%), -138.49 (79.8%). ^{11}B NMR (128 MHz, Chloroform-*d*) δ -0.10. FT-IR (KBr, cm-1): ν 3159.1, 2958.1, 2846.4, 1641.5, 1604.8, 1526.7, 1469.8, 1438.8, 1367.2, 1304.4, 1294.8, 1274.3, 1257.0, 1220.5, 1172.4, 1154.2, 1101.1, 1083.7, 1050.8, 1021.5, 984.9, 976.4, 932.9, 877.7, 845.4, 820.9, 788.2, 764.9, 690.6, 654.3, 630.1, 614.4, 591.3, 575.0, 525.6, 506.0, 469.9. LRMS, m/z 375.1. HRMS (ESI) m/z found (calcd for $C_{19}H_{18}O_5{}^{10}BF_2$): 374.1243 (374.1246).

Compound 3: ^{1}H NMR (400 MHz, Chloroform-*d*) δ 8.44 (s, 1H), 8.07 (dd, *J* = 8.8, 2.3 Hz, 1H), 7.16 (d, *J* = 8.8 Hz, 1H), 6.99 (s, 1H), 4.08 (s, 3H), 2.41 (s, 3H). ^{13}C NMR (101 MHz, Chloroform-*d*) δ 193.61, 192.16, 179.73, 163.44, 137.63, 134.00, 130.44, 120.36, 112.46, 102.82, 56.66, 25.12. ^{19}F NMR (376 MHz, Chloroform-*d*) δ -138.36 (19.9%), -138.42 (80.1%). ^{11}B NMR

(128 MHz, Chloroform-*d*) δ -0.09. FT-IR (KBr, cm^{-1}): 3160.2, 2956.1, 2853.1, 1650.7, 1605.7, 1540.1, 1498.6, 1463.4, 1442.2, 1419.8, 1364.5, 1341.0, 1273.1, 1247.9, 1182.2, 1101.3, 1054.5, 1007.8, 978.1, 921.1, 834.0, 815.3, 757.4, 727.0, 702.8, 649.5, 619.2, 593.1, 572.3, 511.6. LRMS, m/z 529.1. HRMS (ESI) m/z found (calcd for $C_{23}H_{20}O_7{}^{10}B_2F_4$): 527.1291 (527.1296).

Preparation of two-component afterglow materials by doping $BPBF_2$ compounds into organic matrices

For the preparation of $BPBF_2$-PhB-0.1% afterglow materials, 200 μL $BPBF_2$ in dichloromethane (1.0 mg/mL) and 200 mg phenylbenzoate (PhB) solids were added into an agate mortar (diameter = 5 cm). After solvent evaporation, the mixture of $BPBF_2$ and PhB was heated to 100 °C to form molten mixture. Next, keeping the mixture at room temperature to let it solidify to give melt-cast sample. The powder sample can obtained by grinding melt-cast sample into powder.

Afterglow materials with different $BPBF_2$ dopants, different doping concentrations, different small organic matrices can be prepared by the procedure above.

Preparation of $BPBF_2$-COP samples

100~200 mg cyclo olefin polymer (COP) was first dissolved in about 5 mL cyclohexane and then specific volume of $BPBF_2$ dichloromethane solution (1.0 mg/mL) was added into COP solution under sonication. After solvent evaporation, $BPBF_2$-COP samples were obtained.

Preparation of afterglow objects with desired shapes, large-area afterglow films and afterglow dispersions

The $BPBF_2$-PhB-0.1% powders were first heated to 100 °C to form molten mixtures, and then poured into silicone molds with desired shapes. After cooling to room temperature, the targeted solid afterglow objects were obtained. For example, the afterglow letters R, G and B in Figure 5e were made by the above melt casting procedures with the aid of silicone molds.

The large-area afterglow films were prepared with the aid of two quartz plates. Firstly, about 300 mg of $BPBF_2$-PhB-0.1% powders was added onto one quartz plate (10 cm x 10 cm) and heated to 100 °C to form molten mixture. Then the molten mixture was covered by another warmed-up quartz plate (10 cm x 10 cm). After squeezing the air between the two plates, the sandwiched sample was cooled down to room temperature to give 10 cm x 10 cm afterglow films. Films with different size were also prepared by the procedure above. On the basis of the large-area afterglow film, afterglow pattern of **1**-PhB-0.1% in Figure 5d can be obtained by UV excitation through pre-designed masks; the pre-designed masks were made by paper cut.

For the afterglow dispersions, 100 mg Pluronic F-127 (poly(ethylene oxide)-*b*-poly(propylene oxide)-*b*-poly(ethylene oxide), PEO_{100}-*b*-PPO_{68}-*b*-PEO_{100}, the subscript represents the degree of polymerization of each block, Sigma-Aldrich) was dissolved in 10 mL deionized water to form a 10 mg/mL Pluronic F-127 aqueous solution first in a conical flask. Then the conical flask was put into an ultrasonic bath (40 KHz, 80 W) at 85 °C. 50 μL of the molten mixture ($BPBF_2$-PhB-0.1%) was added into the aqueous surfactant solution. After 10-minute ultrasonication, a milky dispersion was obtained that consists of tiny molten droplets of $BPBF_2$-MeOBP-0.1%. By transferring the conical flask to liquid nitrogen bath, the aqueous dispersion of the molten $BPBF_2$-MeOBP-0.1% tiny droplets was frozen immediately. After

standing at room temperature, the frozen samples gradually melt into aqueous afterglow dispersion that exhibits room-temperature afterglow duration of approximately 3 s as observed by naked eyes.

Computational methods

At the begnining, ground state geometry optimization were performed for $BPBF_2$ compounds **1-3** at B3LYP/G/6-31G(d, p) level of theory. The geometries of excited states were also optimized at the TD-DFT/B3LYP/G/6-31G(d, p) level of theory. Excitation energy, spin-orbit coupling matrix elements (SOCME), transition dipole moments were all calculated at the TD-DFT/B3LYP/G/def2-TZVP(-f) level of theory with spin-orbit mean-field (SOMF) methods. All the theoretical calculations mentioned above were performed using the ORCA 5.0.3 software[1–3] and in order to accelerate the computation, the RIJCOSX approximation was used in all DFT calculations. The obtained electronic structures were analyzed by Multiwfn 3.8 software[4]. All isosurface maps to show the electron distribution and electronic transitions were rendered by Visual Molecular Dynamics (VMD 1.9.3) software[5] based on the exported files from Multiwfn 3.8.

In order to make the calculation closer to reality, the "solvent" effect was considered by polarizable continuum model (PCM, CH_2Cl_2), and the geometry of ground states and excited states of compounds **1-3** was re-optimized at the B3LYP/G/6-31G(d,p) and TD-DFT/ B3LYP/G/def2-TZVP(-f) level. The calculation results of excitation energy in CH_2Cl_2 were summarized in Supplementary Table 5-7 and Supplementary Table 11. It can be found that with or without PCM solvent, the B3LYP functional underestimates the excitation energy in both cases.

It is known that one of the major sources of error in TD-DFT calculation originates from self-interaction error (SIE) when most approximate exchange-correlation functionals for Kohn−Sham density functional theory are used[6]. The SIE is large with all local exchange-correlation functionals. The use of hybrid functionals such as B3LYP can reduce SIE but don't eliminate it[7]. Range-separated hybrid functionals have been reported to provide one way to mitigate the systematic error[8,9]. According to recent published work[10,11], the range-separated ωB97X-D functional[12] has been found to exhibit better overall performance on modeling electronically excited states compared to B3LYP functional.

In the present study, to mitigate the errors related to SIE, we evaluated the excited state properties with range-separated functional. Usually, in range-separated functional, the exchange term is splited into a short range domain and a long-range domain. The overall expression is defined by the interelectronic distance r_{12} and error function $erf(\omega_{r_{12}})$:

$$\frac{1}{r_{12}}=\frac{1\text{-}\left[\alpha+\beta\, erf\left(\omega_{r_{12}}\right)\right]}{r_{12}}+\frac{\alpha+\beta\, erf\left(\omega_{r_{12}}\right)}{r_{12}} \quad \#(1)$$

where the α and β are the constants and satisfy $0\leq\alpha,\ \beta\leq1$, and ω is the range-seperation parameter. The ω value is optimized based on the method proposed by Baer[13,14]. In practice, the optimal ω value is obtained by minimizing the $J(\omega)^2$ value defined as following[15]:

$$J(\omega)^2=\sum_{i=0}^{1}\left[\varepsilon_H(N+i)+IP(N+i)\right]^2 \quad \#(2)$$

where ε_H and IP are the HOMO energy and ionization energy of both neutral molecular system

(N) and anion ($N+1$) molecular system, repectively.

After obained the optimal ω value, the geometries of ground states and excited states were optimized at ωB97XD/6-31G(d, p) level of theory. Frequency analysis was preformed at the same theoretical level to validate the presence of minimum and to generate the hessian matrix, which is needed for the following rate calculation. The geometry optimization and frequency analysis were performed with Gaussian 16 (version A03)[16]. Excitation energy, spin-orbit coupling matrix elements (SOCME), transition dipole moments and all the other excited states related property were calculated with ωB97XD's successor ωB97X-D3 functional[17] and def2-TZVP(-f) basis set by ORCA 5.0.3 software. The excitation energy of triplet excited states at S_1 geometry of compound **1-3** was quite near the experimental value.

The obtained electronic structures were analyzed by Multiwfn 3.8 software[4]. All isosurface maps to show the electron distribution and electronic transitions were rendered by Visual Molecular Dynamics (VMD 1.9.3) software[5] based on the exported files generated by Multiwfn 3.8.

Theoretical prediction of rate constants

According to Fermi's Golden Rule (FGR)[18], the fluorescence ($S_n \rightarrow S_0$) and phosphorescence ($T_n \rightarrow S_0$) rates can be calculated as

$$k_{F,S_n} = \frac{4\Delta E_{AD}(S_n\text{-}S_0)^3}{3\epsilon_0 \hbar^4 c^3}\mu_F(S_n\text{-}S_0)^2 \ \#(3)$$

$$k_{P,T_n} = \frac{1}{3}\frac{4\Delta E_{AD}(T_n\text{-}S_0)^3}{3\epsilon_0 \hbar^4 c^3}\mu_P(T_n\text{-}S_0)^2 \ \#(4)$$

where ΔE_{AD} is the adiabatic energy difference between excited state (S_n or T_n) and ground state (S_0), ϵ_0 is the vacuum electric permittivity, $\hbar$ is the reduced Planck constant, c is the speed of light, $\mu_F(S_n - S_0)$ is the transition dipole moment between S_n and S_0, and $\mu_P(T_n\text{-}S_0)$ is the transition dipole moment between T_n and S_0.

For the intersystem crossing rate (k_{ISC}), refer to recent Kaji's work which based on Fermi's Golden rule[19,20], it can be written as

$$k_{ISC} = \frac{2\pi}{\hbar}|\langle S_1|\mathbf{H_{SO}}|T_1\rangle|^2 L\big(\Delta E_{AD}(S_m\text{-}T_n)\big) \ \#(5)$$

$$L\big(\Delta E_{AD}(S_m\text{-}T_n)\big) = \frac{1}{\pi}\frac{\gamma}{\Delta E_{AD}(S_m\text{-}T_n)^2+\gamma^2} \ \#(6)$$

where ΔE_{AD} is the adiabatic energy difference between singlet excited state (S_m) and triplet excited state (T_n), $L(\Delta E_{AD}(S_m\text{-}T_n))$ is the Lorentzian distribution function as function of energy, and γ is the broadening of Lorentzian distribution function. Accurate determination of γ is hard, fortunately for $BPBF_2$ compounds, the γ has little effect to the value of transition rate (Supplementary Fig. 17). γ was set to be 1000 cm^{-1}.

When the $S_m \rightarrow T_n$ transition is an energy-downhill transition, the energy-uphill $T_n \rightarrow S_m$ rate constant is calculated as

$$\frac{1}{3}k_{ISC}(S_m\text{-}T_n)\cdot exp[-\beta_B \Delta E_{FC}(S_m\text{-}T_n)] \ \#(7)$$

where β_B is the inverse temperature ($\beta^{-1} = k_BT$, where k_B is the Boltzmann constant and T is the temperature).

The intersystem crossing rates were also evaluated using semi-classical Marcus rate expression[21,22].

$$k_{ISC}(S_m\text{-}T_n)=\frac{2\pi}{\hbar}|\langle S_m|\mathbf{H}_{\mathbf{SO}}|T_n\rangle|^2(4\pi\lambda k_BT)^{-\frac{1}{2}}exp\left(-\frac{\left(\lambda+\Delta E_{AD}(S_m\text{-}T_n)\right)^2}{4\lambda k_BT}\right) \#(8)$$

where λ is the reorganization energy.

The internal conversion rates (k_{IC}) were calculated using the following simplified formula proposed by Kaji[19,20].

$$k_{IC}(T_m\text{-}T_n)=\sum_\alpha k_{IC,\alpha}(T_m\text{-}T_n) \ \#(9)$$

$$k_{IC,\alpha}(T_m\text{-}T_n)=\frac{R_\alpha}{\hbar}LSF_{IC,\alpha} \ \#(10)$$

$$R_\alpha=\frac{\hbar^2V_\alpha^{\ 2}}{\Delta E_{FC}(T_m\text{-}T_n)^2} \ \#(11)$$

$$LSF_{IC,\alpha}=2\pi\sinh\left(\frac{1}{2}\beta\hbar\omega_\alpha\right)\sum_{v_\alpha}exp\left\{-\left(\frac{1}{2}+v_\alpha\right)\beta\hbar\omega_\alpha\right\}$$
$$\times\frac{1}{\pi S}\left\{\frac{v_\alpha\gamma}{\{\Delta E_{AD}(T_m\text{-}T_n)\}^2+\gamma^2}+\frac{(v_\alpha+1)\gamma}{\{\Delta E_{AD}(T_m\text{-}T_n)\}^2+\gamma^2}\right\} \ \#(12)$$

$$S=\frac{1}{4\pi\epsilon_0}\frac{e^2}{a_Bhc} \ \#(13)$$

where $k_{IC}(T_m-T_n)$ is the internal conversion rate between triplet excited states T_m and T_n ($m > n \geqslant 1$), V_α is the vibronic coupling between T_m and T_n for the αth vibrational mode, $\Delta E_{FC}(T_m\text{-}T_n)$ is the Franck-Condon T_m-T_n energy difference, ω_α and v_α are the vibrational quantum number and angular frequency for the αth vibrational mode, respectively, $\Delta E_{AD}(T_m\text{-}T_n)$ is the adiabatic T_m-T_n energy difference, e is the elementary charge, a_B is the Bohr radius. The energy-downhill internal conversion rate ($k_{IC}(T_n\text{-}T_m)$, where $m > n \geqslant 1$) is calculated from energy-uphill rate as following.

$$k_{IC}(T_m\text{-}T_n)\cdot exp[-\beta_B\Delta E_{FC}(S_m\text{-}T_n)] \ \#(14)$$

In this study, the vibronic coupling V_α is calculated by following expression related to Rhys-Huang factor[23]:

$$S_\alpha=\frac{V_\alpha^{\ 2}}{2\hbar\omega_\alpha^{\ 3}} \ \#(15)$$

where S_α is the Rhys-Huang factor for the αth vibrational mode.

The internal conversion rate calculations were also performed with FCClasses3.0[24]. The rate calculations made use of the adiabatic hessian model and time-dependent variants. Temperature was set to 300K to be consistent with other rate calculations and a 0.001 eV spectra resolution was used. HWHM (Half width at half maximum) value used for broadening was set to 0.001 eV. Calculation with different HWHM verified that the internal conversion rate regardless to the HWHM value (Supplementary Fig.18).

4. Supplementary Discussion

Further discussion to rule out the possibility that the 421 nm delayed emission is originated from TADF.

It is known that microenvironment has significant influence on the position of fluorescence peak of luminescent compounds with intramolecular charge transfer character. Although the peak of fluorescence spectra of compound **1** in dichloromethane solution (423 nm, Supplementary Table 1) is very close to that of the higher-energy delayed emission band (1 ms delay) of **1**-PhB-0.1% powders (421 nm, Figure 1c), the 421 nm delayed emission of **1**-PhB-0.1% powders *cannot* simply be attributed to TADF because the microenvironment of compound **1** in dichloromethane solution is *different* from that in PhB matrices. Therefore, we compare the steady-state emission spectra and delayed emission spectra (1 ms delay) of **1**-PhB-0.1% powders (both of which are collected in PhB matrices) for the assignment of emission bands.

The higher-energy band in delayed emission spectra (1 ms delay) has a maximum at 421 nm (Figure 1c), which is shorter than the emission maxima in the steady-state emission spectra (λ_F = 437 nm, Figure 1c). In the steady-state emission spectra, because of the coexistence of fluorescence and phosphorescence, the presence of phosphorescence may cause slightly red shift of the fluorescence spectra. In the delayed emission spectra (1 ms delay), the phosphorescence signals can also cause red shift of higher-energy delayed emission band. Therefore, despite the slight interference of phosphorescence, the observation that the higher-energy band in the delayed emission spectra has an emission maximum of shorter wavelength than the steady-state emission spectra is still correct.

Usually, the TADF afterglow bands possess very similar and even identical emission maxima to steady-state emission bands, when the luminescent dopants are molecularly dispersed in organic matrices[25,26] (*Angew. Chem. Int. Ed.* **2021**, *60*, 17138; *Adv. Funct. Mater.* **2021**, 2110207). In the present case, by comparing the steady-state emission spectra and delayed emission spectra (1 ms delay), it is found that TADF signals is insignificant in the delayed emission spectra; the 421 nm delayed band doesn't coincide with the 437 nm fluorescence band in **1**-PhB-0.1% system.

For the reverse ISC, from the results obtained by both B3LYP and ωB97X-D3 methods, it is found that, at the optimized geometry of either T_1 *or* T_n (n = 2 for compounds **1** and **2**, n =3 for compound **3**), the T_1 and T_n levels are much lower than S_1 levels (Table 2). Given that reverse ISC starts from triplet excited states, these results suggest that reverse ISC is not likely to occur. The corresponding rate constants of reverse ISC have also been calculated to show small values (Supplementary Table 24-29), which can explain the absence of TADF afterglow in the experimental observations.

In the main text, the $k(T_1\text{-}T_n)/k(T_n\text{-}T_1)$ ratios have been calculated to be on the order of 10^{-3}~10^{-2} at 300 K (Table 4). The $k_P(T_n)$ values of 10^3~10^4 s^{-1} at T_n geometries (n = 2 for compounds **1** and **2**, n =3 for compound **3**) are found to be much larger than $k_P(T_1)$ values of 10^0~10^1 s^{-1} at T_1 geometries, exhibiting $k_P(T_n)/k_P(T_1)$ ratios of 10^2~10^3. Given that the relative emission intensity of RTP(T_n)/RTP(T_1) is proportional to $k(T_1\text{-}T_n)/k(T_n\text{-}T_1) \times k_P(T_n)/k_P(T_1)$, the theoretical calculations support the experimental observation of RTP(T_n)/RTP(T_1) dual emission in

the delayed spectra.

All the above experimental studies, theoretical calculations and analyses support that the higher-energy delayed emission bands originate from room-temperature phosphorescence of higher triplet excited states, rather than from TADF.

5. Supplementary References

1. Neese, F. The ORCA program system. *WIREs Comput. Mol. Sci.* **2**, 73–78 (2012).
2. Neese, F., Wennmohs, F., Becker, U. & Riplinger, C. The ORCA quantum chemistry program package. *J. Chem. Phys.* **152**, 224108 (2020).
3. Neese, F. Software update: The ORCA program system—Version 5.0. *WIREs Comput. Mol. Sci.* **12**, e1606 (2022).
4. Lu, T. & Chen, F. Multiwfn: A multifunctional wavefunction analyzer. *J. Comput. Chem.* **33**, 580–592 (2012).
5. Humphrey, W., Dalke, A. & Schulten, K. VMD: Visual molecular dynamics. *J. Mol. Graphics* **14**, 33–38 (1996).
6. Bao, J. L., Gagliardi, L. & Truhlar, D. G. Self-Interaction Error in Density Functional Theory: An Appraisal. *J. Phys. Chem. Lett.* **9**, 2353–2358 (2018).
7. Bursch, M., Mewes, J.-M., Hansen, A. & Grimme, S. Best-Practice DFT Protocols for Basic Molecular Computational Chemistry**. *Angew. Chem., Inter. Ed.* **61**, e202205735 (2022).
8. Autschbach, J. Charge-Transfer Excitations and Time-Dependent Density Functional Theory: Problems and Some Proposed Solutions. *ChemPhysChem* **10**, 1757–1760 (2009).
9. Autschbach, J. & Srebro, M. Delocalization Error and "Functional Tuning" in Kohn–Sham Calculations of Molecular Properties. *Acc. Chem. Res.* **47**, 2592–2602 (2014).
10. Liang, J., Feng, X., Hait, D. & Head-Gordon, M. Revisiting the Performance of Time-Dependent Density Functional Theory for Electronic Excitations: Assessment of 43 Popular and Recently Developed Functionals from Rungs One to Four. *J. Chem. Theory Comput.* **18**, 3460–3473 (2022).
11. Sarkar, R., Boggio-Pasqua, M., Loos, P.-F. & Jacquemin, D. Benchmarking TD-DFT and Wave Function Methods for Oscillator Strengths and Excited-State Dipole Moments. *J. Chem. Theory Comput.* **17**, 1117–1132 (2021).

12. Chai, J.-D. & Head-Gordon, M. Long-range corrected hybrid density functionals with damped atom–atom dispersion corrections. *Phys. Chem. Chem. Phys.* **10**, 6615–6620 (2008).

13. Baer, R., Livshits, E. & Salzner, U. Tuned Range-Separated Hybrids in Density Functional Theory. *Annu. Rev. Phys. Chem.* **61**, 85–109 (2010).

14. Kronik, L., Stein, T., Refaely-Abramson, S. & Baer, R. Excitation Gaps of Finite-Sized Systems from Optimally Tuned Range-Separated Hybrid Functionals. *J. Chem. Theory Comput.* **8**, 1515–1531 (2012).

15. Sun, H. *et al.* Impact of Dielectric Constant on the Singlet–Triplet Gap in Thermally Activated Delayed Fluorescence Materials. *J. Phys. Chem. Lett.* **8**, 2393–2398 (2017).

16. Frisch, M. J. *et al.* Gaussian 16 Revision A.03. (2016).

17. Lin, Y.-S., Li, G.-D., Mao, S.-P. & Chai, J.-D. Long-Range Corrected Hybrid Density Functionals with Improved Dispersion Corrections. *J. Chem. Theory Comput.* **9**, 263–272 (2013).

18. Schatz, G. C. & Ratner, M. A. *Quantum Mechanics in Chemistry*. (Courier Corporation, 2002).

19. Shizu, K. & Kaji, H. Theoretical Determination of Rate Constants from Excited States: Application to Benzophenone. *J. Phys. Chem. A* **125**, 9000–9010 (2021).

20. Shizu, K. & Kaji, H. Comprehensive understanding of multiple resonance thermally activated delayed fluorescence through quantum chemistry calculations. *Commun. Chem.* **5**, 1–6 (2022).

21. Olivier, Y. *et al.* Nature of the singlet and triplet excitations mediating thermally activated delayed fluorescence. *Phys. Rev. Mater.* **1**, 075602 (2017).

22. Brédas, J.-L., Beljonne, D., Coropceanu, V. & Cornil, J. Charge-Transfer and Energy-Transfer Processes in π-Conjugated Oligomers and Polymers: A Molecular Picture. *Chem. Rev.* **104**, 4971–5004 (2004).

23. Kato, T., Haruta, N. & Sato, T. *Vibronic Coupling Density: Understanding Molecular Deformation*. (Springer, 2021).

24. Cerezo, J. & Santoro, F. FCclasses3: Vibrationally-resolved spectra simulated at the edge of the harmonic approximation. *J. Comput. Chem.* **44**, 626–643 (2023).

25. Wang, X. *et al.* TADF-Type Organic Afterglow. *Angew. Chem., Inter. Ed.* **60**, 17138–17147 (2021).

26. Pan, Y. *et al.* Highly Efficient TADF-Type Organic Afterglow of Long Emission Wavelengths.

Adv. Funct. Mater. **32**, 2110207 (2022).
