# Supplementary material for: A direct observation of up-converted room-temperature phosphorescence in an anti-Kasha dopant-matrix system
Source: Nat Commun. 2023 Apr 8;14:1987. doi: 10.1038/s41467-023-37662-y (PMC10082826; doi:10.1038/s41467-023-37662-y)
Supplement: Supplementary file 3 — Description of Additional Supplementary Files [file 41467_2023_37662_MOESM3_ESM.pdf]

File name: Supplementary Data 1

Description: CIF file of compound 1.
