# Supplementary material for: A direct observation of up-converted room-temperature phosphorescence in an anti-Kasha dopant-matrix system
Source: Nat Commun. 2023 Apr 8;14:1987. doi: 10.1038/s41467-023-37662-y (PMC10082826; doi:10.1038/s41467-023-37662-y)

checkCIF/PLATON report

Structure factors have been supplied for datablock(s) mo_dm21102_0m

THIS REPORT IS FOR GUIDANCE ONLY. IF USED AS PART OF A REVIEW PROCEDURE FOR PUBLICATION, IT SHOULD NOT REPLACE THE EXPERTISE OF AN EXPERIENCED CRYSTALLOGRAPHIC REFEREE.

No syntax errors found. CIF dictionary Interpreting this report

Datablock: mo_dm21102_0m

Bond precision: C-C = 0.0034 A Wavelength=0.71073

Cell:	a=10.3880(18)	b=16.326(3)	c=19.538(3)
	alpha=90	beta=90	gamma=90
Temperature:	296 K		

	Calculated	Reported
Volume	3313.5(10)	3313.5(10)
Space group	P b c a	P b c a
Hall group	-P 2ac 2ab	-P 2ac 2ab
Moiety formula	C18 H15 B F2 O4	C18 H15 B F2 O4
Sum formula	C18 H15 B F2 O4	C18 H15 B F2 O4
Mr	344.11	344.11
Dx,g cm-3	1.380	1.380
Z	8	8
Mu (mm-1)	0.110	0.110
F000	1424.0	1424.0
F000'	1424.94	
h,k,lmax	13,21,25	13,21,25
Nref	3737	3721
Tmin,Tmax	0.980,0.998	0.671,0.746
Tmin'	0.980	

Correction method= # Reported T Limits: Tmin=0.671 Tmax=0.746
AbsCorr = MULTI-SCAN

Data completeness= 0.996 Theta(max)= 27.321

R(reflections)= 0.0517(1908) wR2(reflections)= 0.1799(3721)

S = 0.978 Npar= 228

The following ALERTS were generated. Each ALERT has the format
test-name_ALERT_alert-type_alert-level.
Click on the hyperlinks for more details of the test.

Alert level C

```
PLAT241_ALERT_2_C High   'MainMol' Ueq as Compared to Neighbors of         O1 Check
PLAT241_ALERT_2_C High   'MainMol' Ueq as Compared to Neighbors of         O2 Check
PLAT242_ALERT_2_C Low    'MainMol' Ueq as Compared to Neighbors of         B1 Check
PLAT905_ALERT_3_C Negative K value in the Analysis of Variance ...    -11.842 Report
PLAT905_ALERT_3_C Negative K value in the Analysis of Variance ...     -1.531 Report
```

Alert level G

```
PLAT380_ALERT_4_G Incorrectly? Oriented X(sp2)-Methyl Moiety .....        C10 Check
PLAT912_ALERT_4_G Missing # of FCF Reflections Above STh/L=  0.600         15 Note
PLAT933_ALERT_2_G Number of OMIT Records in Embedded .res File ...          1 Note
PLAT954_ALERT_1_G Reported (CIF) and Actual (FCF) Kmax Differ by .          1 Units
PLAT978_ALERT_2_G Number C-C Bonds with Positive Residual Density.          1 Info
```

0 **ALERT level A** = Most likely a serious problem - resolve or explain
0 **ALERT level B** = A potentially serious problem, consider carefully
5 **ALERT level C** = Check. Ensure it is not caused by an omission or oversight
5 **ALERT level G** = General information/check it is not something unexpected

1 ALERT type 1 CIF construction/syntax error, inconsistent or missing data
5 ALERT type 2 Indicator that the structure model may be wrong or deficient
2 ALERT type 3 Indicator that the structure quality may be low
2 ALERT type 4 Improvement, methodology, query or suggestion
0 ALERT type 5 Informative message, check

It is advisable to attempt to resolve as many as possible of the alerts in all categories. Often the minor alerts point to easily fixed oversights, errors and omissions in your CIF or refinement strategy, so attention to these fine details can be worthwhile. In order to resolve some of the more serious problems it may be necessary to carry out additional measurements or structure refinements. However, the purpose of your study may justify the reported deviations and the more serious of these should normally be commented upon in the discussion or experimental section of a paper or in the "special_details" fields of the CIF. checkCIF was carefully designed to identify outliers and unusual parameters, but every test has its limitations and alerts that are not important in a particular case may appear. Conversely, the absence of alerts does not guarantee there are no aspects of the results needing attention. It is up to the individual to critically assess their own results and, if necessary, seek expert advice.

Publication of your CIF in IUCr journals

A basic structural check has been run on your CIF. These basic checks will be run on all CIFs submitted for publication in IUCr journals (*Acta Crystallographica*, *Journal of Applied Crystallography*, *Journal of Synchrotron Radiation*); however, if you intend to submit to *Acta Crystallographica Section C* or *E* or *IUCrData*, you should make sure that full publication checks are run on the final version of your CIF prior to submission.

Publication of your CIF in other journals

Please refer to the *Notes for Authors* of the relevant journal for any special instructions relating to CIF submission.

PLATON version of 13/07/2021; check.def file version of 13/07/2021

Datablock mo_dm21102_0m - ellipsoid plot

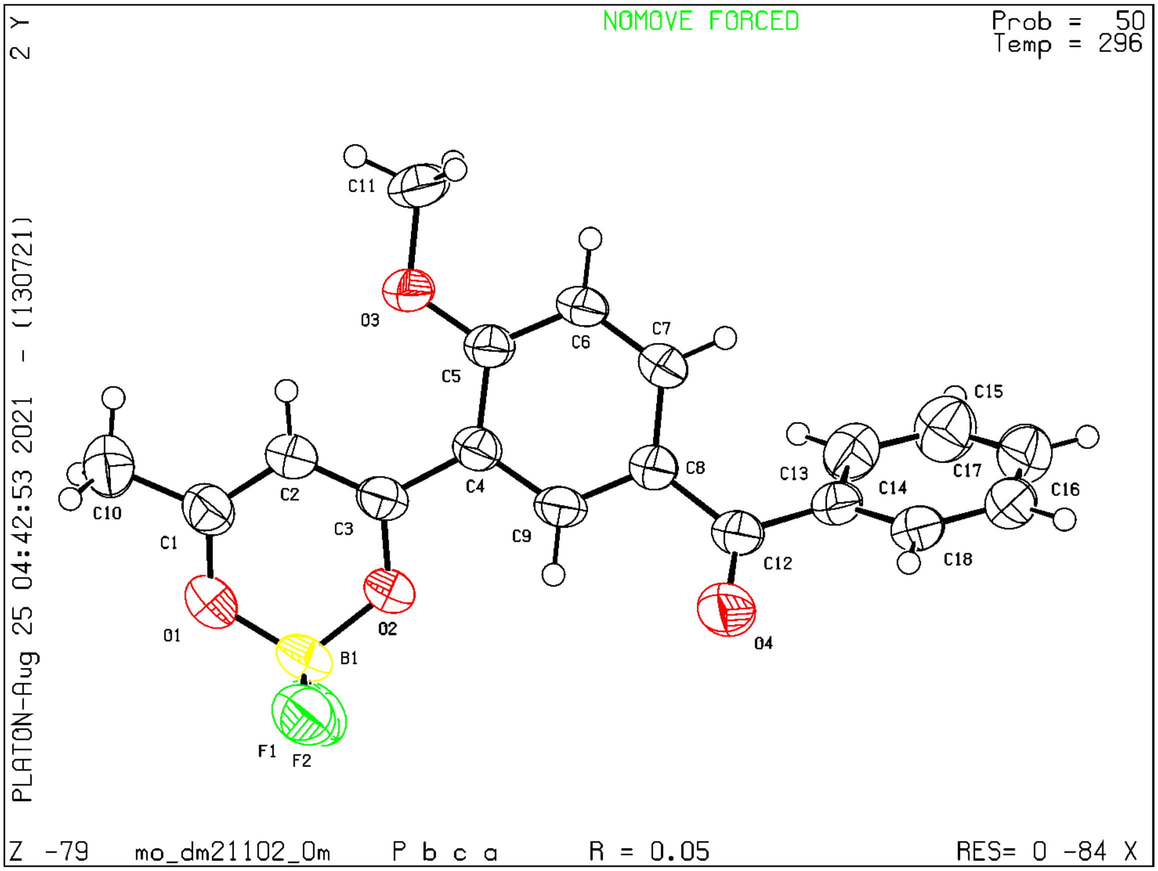

Supplement: Supplementary file 4 — Supplementary Data 1 [file 41467_2023_37662_MOESM4_ESM.zip › 362327_3_supp_7452530_rrrm5n.pdf]
